# Characterization of the Arn lipopolysaccharide modification system essential for zeamine resistance unveils its new roles in *Dickeya oryzae* physiology and virulence

**DOI:** 10.1111/mpp.13386

**Published:** 2023-09-22

**Authors:** Zhibin Liang, Luhao Huang, Huidi Liu, Ying Zheng, Jiani Feng, Zurong Shi, Yufan Chen, Mingfa Lv, Jianuan Zhou, Lian‐Hui Zhang, Shaohua Chen

**Affiliations:** ^1^ Guangdong Province Key Laboratory of Microbial Signals and Disease Control, Integrative Microbiology Research Centre South China Agricultural University Guangzhou China; ^2^ Guangdong Laboratory for Lingnan Modern Agriculture Guangzhou China; ^3^ School of Biological Engineering Huainan Normal University Huainan China; ^4^ Research Center of Chinese Herbal Resource Science and Engineering Guangzhou University of Chinese Medicine Guangzhou China; ^5^ College of Plant Protection Fujian Agriculture and Forestry University Fuzhou China

**Keywords:** antimicrobial resistance, *arn* operon, cell division, lipopolysaccharide, motility

## Abstract

The zeamines produced by *Dickeya oryzae* are potent polyamine antibiotics and phytotoxins that are essential for bacterial virulence. We recently showed that the RND efflux pump DesABC in *D. oryzae* confers partial resistance to zeamines. To fully elucidate the bacterial self‐protection mechanisms, in this study we used transposon mutagenesis to identify the genes encoding proteins involved in zeamine resistance in *D. oryzae* EC1. This led to the identification of a seven‐gene operon, *arn*_EC1_, that encodes enzyme homologues associated with lipopolysaccharide modification. Deletion of the *arn*_EC1_ genes in strain EC1 compromised its zeamine resistance 8‐ to 16‐fold. Further deletion of the *des* gene in the *arn*_EC1_ mutant background reduced zeamine resistance to a level similar to that of the zeamine‐sensitive *Escherichia coli* DH5α. Intriguingly, the *arn*_EC1_ mutants showed varied bacterial virulence on rice, potato, and Chinese cabbage. Further analyses demonstrated that ArnBCAT_EC1_ are involved in maintenance of the bacterial nonmucoid morphotype by repressing the expression of capsular polysaccharide genes and that ArnB_EC1_ is a bacterial virulence determinant, influencing transcriptional expression of over 650 genes and playing a key role in modulating bacterial motility and virulence. Taken together, these findings decipher a novel zeamine resistance mechanism in *D*. *oryzae* and document new roles of the Arn enzymes in modulation of bacterial physiology and virulence.

## Graphical abstract

The *arn*_EC1_ genes play multiple roles in bacterial physiology and virulence in *Dickeya oryzae*.

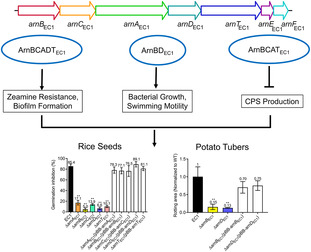

## 1 INTRODUCTION

*Dickeya oryzae* is a gram‐negative bacterial plant pathogen, recently separated from *Dickeya zeae* (Wang et al., 2020). The diseases caused by *D. oryzae* pose a great threat to rice production in Asian and European countries and potato production in Australia (Bertani et al., 2013; Liu et al., 2013; Pritchard et al., 2013). Unlike most *Dickeya* species, which infect only dicot plants, *D. oryzae* is capable of infecting both monocot and dicot plants (Hussain et al., 2008). Similar to the well‐studied *Dickeya dadantii* (Reverchon & Nasser, 2013), *D. oryzae* produces various cell wall‐degrading enzymes that contribute to its virulence and pathogenesis (Lv et al., 2018; Reverchon & Nasser, 2013; Zhou et al., 2016). In addition, our recent studies unveiled two key virulence determinants in *D. oryzae*, that is, bacterial motility and the group of zeamine phytotoxins. Mutants defective in cell motility are severely compromised in rice seed invasion and systemic infection (Chen et al., 2020, 2022; Hussain et al., 2008; Lv et al., 2018, 2022; Shi et al., 2019; Zhou et al., 2016). Zeamines are a group of structurally related polyamine compounds produced by *D. oryzae*, which are phytotoxins that inhibit rice seed germination and damage plant tissues (Cheng et al., 2013; Zhou et al., 2011). Inactivation of *zmsA*, a key gene required for zeamine biosynthesis, drastically reduces the virulence of *D. oryzae* EC1 against rice, Chinese cabbage, and potato (Zhou et al., 2011).

In addition to their role as phytotoxins, zeamines are also potent antimicrobial agents with broad‐spectrum activity against bacteria, fungi, nematodes, and oomycetes (Hellberg et al., 2015; Liao et al., 2015; Masschelein, Clauwers, Awodi, et al., 2015; Wu et al., 2010). Zeamines inhibit bacterial growth by damaging the cell envelope of gram‐negative bacteria in a way similar to cationic antimicrobial peptides (CAMPs) (Masschelein, Clauwers, Stalmans et al., 2015). As a producer of zeamines, self‐protection mechanisms against these polyamine antimicrobials are of vital importance for the survival and growth of *D. oryzae*, with which the pathogen could tolerate a several‐hundred‐fold higher dose of zeamines than zeamine‐sensitive bacterial species such as *Escherichia coli* (Liang et al., 2019; Wu et al., 2010). We found recently that zeamines could induce the expression of the *desAB* genes, which encode components of the zeamine‐specific RND (RND refers to resistance, nodulation, and cell division) efflux pump DesABC (Liang et al., 2019). However, given that the minimal inhibitory concentration (MIC) of zeamines for *D. oryzae* EC1 was more than 500‐fold higher than that for the zeamine‐sensitive strain *E. coli* DH5α, but only 8‐ to 32‐fold higher than those for the *desABC* gene mutants, we hypothesized that DesABC might not represent the sole zeamine resistance mechanism in *D. oryzae* (Liang et al., 2019).

The *arn* (*arnBCADTEF*) or *pmr* (*pmrHFIJKLM*) operon is widely conserved in gram‐negative bacterial species, including the pathogenic strains belonging to *Salmonella enterica* (Gunn et al., 2000), *Pseudomonas aeruginosa* (McPhee et al., 2003), *Yersinia pestis* (Winfield et al., 2005), *Proteus mirabilis* (Jiang et al., 2010), *Serratia marcescens* (Lin et al., 2014), *Proteus vulgaris* (Baron et al., 2018), *Klebsiella pneumoniae* (Cheung et al., 2020), and *D. dadantii* (Costechareyre et al., 2013). The products of the *arn* operon are responsible for the biosynthesis of 4‐amino‐4‐deoxy‐l‐arabinose (l‐Ara4N) and transfer of l‐Ara4N to the lipid A moiety of lipopolysaccharide (LPS) in the cell envelope of gram‐negative bacteria (Breazeale et al., 2005; Trent et al., 2001; Yan et al., 2007). As addition of l‐Ara4N creates a positively charged LPS that could minimize the binding of CAMPs, the Arn LPS modification system thus becomes an important bacterial resistance mechanism against CAMPs such as polymyxins (Gunn et al., 2000; Poirel et al., 2017). It was also noted that inactivation of the *arn* genes could weaken bacterial virulence, which was believed to be due to poor survival of the *arn* mutants in the presence of antimicrobial peptides from host organisms (Costechareyre et al., 2013; Gunn et al., 2000).

To elucidate the uncharacterized zeamine resistance mechanisms in *D. oryzae*, we generated transposon insertional mutants using the zeamine‐minus mutant ∆*zmsA* as the parental strain, which was derived from *D. oryzae* EC1 (Zhou et al., 2011), and screened for mutants with increased zeamine sensitivity. We found that mutation of the *arn* homologues (designated as the *arn*_EC1_ genes) in strain ∆*zmsA* significantly decreased its resistance to zeamines and polymyxin B. Intriguingly, the individual mutants of the *arn*_EC1_ genes showed substantially varied levels of virulence against rice, Chinese cabbage, and potato, which led us to speculate that the *arn*_EC1_ products may have other biological functions in addition to their role in zeamine and CAMP resistance. Subsequent analyses unravelled the role of ArnBCAT_EC1_ in maintaining the nonmucoid morphotype of *D*. *oryzae* EC1 and the implication of ArnB_EC1_ in modulation of bacterial cell division and motility.

## 2 RESULTS

### 2.1 Identification of zeamine resistance genes in *D. oryzae*

**FIGURE 1:**
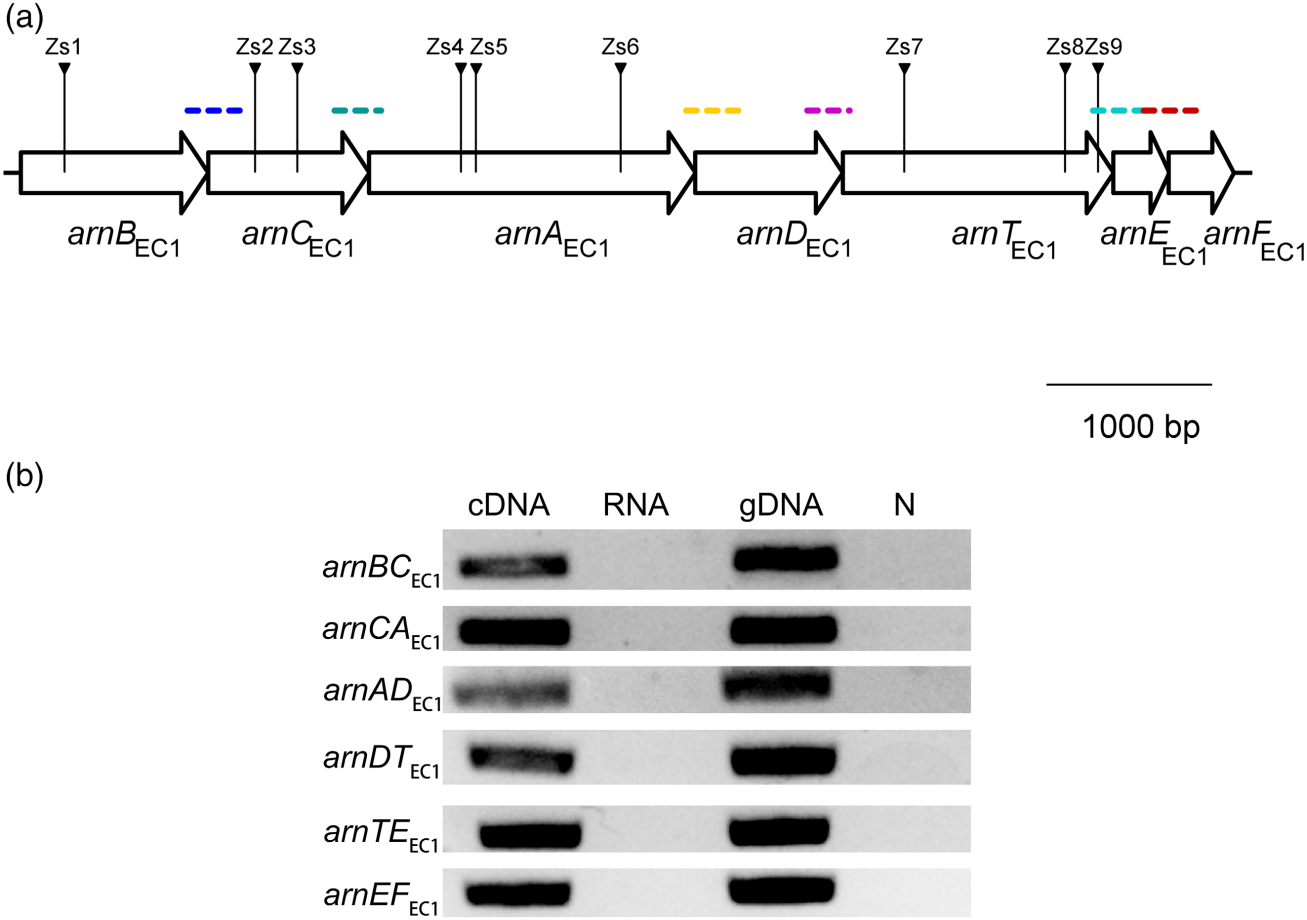
Genetic organization and characterization of the *arn*_EC1_ operon. (a) The genetic organization of the *arn*_EC1_ operon. The arrows indicate the positions of transposon insertions in Zs strains. (b) Reverse transcription PCR (RT‐PCR) assay of the intergenic region of two neighbouring *arn*_EC1_ genes. The dash lines in (a) show the amplified regions in the RT‐PCR assay. Genomic DNA (gDNA) and RNA obtained from the bacterial culture of *Dickeya oryzae* EC1 were amplified as controls. N indicates the negative control without nucleic acid.

To identify zeamine resistance genes in *D. oryzae* EC1, its zeamine‐minus mutant ∆*zmsA* was mutated with the mariner‐based transposon carried by pBT20 (Seet & Zhang, 2011). Mutant ∆*zmsA* was chosen for transposon mutagenesis to eliminate a putative toxic effect of the self‐produced zeamines on the growth of transposon insertional mutants that are sensitive to zeamines. The resultant library was screened for mutants with altered sensitivity to zeamines. After screening about 11,800 transposon insertional mutants, nine mutants designated as Zs1–Zs9 showing higher levels of zeamine sensitivity than the parental strain ∆*zmsA* were obtained. The flanking regions adjacent to the insertion sites were amplified by fusion primer and nested integrated PCR (FPNI‐PCR; Wang et al., 2011) and sequenced for BLASTn analysis to localize the insertion sites. The results showed that the mutants Zs1–Zs9 harbour transposon insertions within a putative seven‐gene operon (Figure 1a, Table S1), which shows over 88% similarity at the nucleotide level to the previously characterized *arn* operon of *D*. *dadantii* 3937 (Costechareyre et al., 2013). This putative operon was hence named the *arn*_EC1_ operon (Figure 1a) and selected for subsequent analysis. Two Zs mutants, Zs4 and Zs7, with transposon insertions in the *arnA*_EC1_ and *arnT*_EC1_ genes, respectively, were randomly selected for the MIC assay. The results showed that they were 8‐fold more sensitive to zeamines than their parental strain ∆*zmsA*. In trans expression of *arnT*_EC1_ in the mutant Zs7 fully restored its zeamine resistance to the level of strain ∆*zmsA* (Table 1), suggesting that the *arn*_EC1_ genes are involved in *D. oryzae* resistance to zeamines.

**TABLE 1:** Minimal inhibitory concentrations (MICs) of zeamines and polymyxin B for *Dickeya* strains and *Escherichia coli* DH5α.

Strain		MIC (μg/mL)	
		Zeamines	Polymyxin B
*D. oryzae*	∆*zmsA*	1800	>1000
	Zs4	225	ND
	Zs7	225	ND
	Zs7(pBB‐*arnT*_EC1_)	1800	ND
	∆*zmsA*∆*arnB*_EC1_	112.5	0.4
	∆*zmsA*∆*arnC*_EC1_	225	1.6
	∆*zmsA*∆*arnA*_EC1_	225	3.2
	∆*zmsA*∆*arnD*_EC1_	112.5	1.6
	∆*zmsA*∆*arnT*_EC1_	112.5	1.6
	∆*zmsA*∆*arnE*_EC1_	225	1.6
	∆*zmsA*∆*arnF*_EC1_	225	1.6
	∆*zmsA*∆*arnB*_EC1_∆*desB*	14.1	0.4
	∆*zmsA*∆*arnB*_EC1_(pBB‐*arnB*_EC1_)	1800	>1000
	∆*zmsA*∆*arnC*_EC1_(pBB‐*arnC*_EC1_)	1800	>1000
	∆*zmsA*∆*arnA*_EC1_(pBB‐*arnA*_EC1_)	1800	>1000
	∆*zmsA*∆*arnD*_EC1_(pBB‐*arnD*_EC1_)	1800	>1000
	∆*zmsA*∆*arnT*_EC1_(pBB‐*arnT*_EC1_)	1800	>1000
*D. dadantii*	3937	28.1	1.6
	3937(pAmob, pBB)	28.1	1.6
	3937(pAmob‐*arnBCADTEF*_EC1_, pBB‐*ugd*_EC1_)	56.3	100
*E. coli*	DH5α	14.1	0.4

*Note*: The values are the MICs of zeamines or polymyxin B for different bacterial strains as determined by the broth microdilution method. The wells of 96‐well plates containing Luria‐Bertani medium with twofold dilutions of zeamines were inoculated with fresh bacterial cultures and incubated at 28°C or 37°C for 18 h. The lowest concentration of zeamines that prevents the visible growth of each bacterial strain is considered as the MIC. This experiment was performed twice, each with three replicates. In the three replicates, the MICs for each bacterial strain were the same. ND, not determined.

### 2.2 *arnBCADTEF*_EC1_ constitute an operon encoding proteins involved in zeamine and polymyxin B resistance in *D. oryzae*

Bioinformatics analysis showed that in the *arn*_EC1_ operon (Figure 1a), *arnB*_EC1_ (NCBI accession no. W909_RS19690) and *arnC*_EC1_ (NCBI accession no. W909_RS19695) encode a putative UDP‐4‐amino‐4‐deoxy‐l‐arabinose‐oxoglutarate aminotransferase and a putative undecaprenyl phosphate‐4‐deoxy‐4‐formamido‐l‐arabinose transferase, respectively. *ArnA*_EC1_ (NCBI accession no. W909_RS19700) encodes a putative decarboxylase/UDP‐4‐amino‐4‐deoxy‐l‐arabinose formyltransferase, and *arnD*_EC1_ (NCBI accession no. W909_RS19705) and *arnT*_EC1_ (NCBI accession no. W909_RS19710), located downstream in the operon, encode a putative 4‐deoxy‐4‐formamido‐l‐arabinose‐phosphoundecaprenol deformylase and an undecaprenyl phosphate‐α‐4‐amino‐4‐deoxy‐l‐arabinose arabinosyl transferase, respectively. The last two genes, *arnE*_EC1_ (NCBI accession no. W909_RS19715) and *arnF*_EC1_ (NCBI accession no. W909_RS19720), encode two putative l‐Ara4N‐phosphoundecaprenol flippase subunits. The proteins encoded by the *arn*_EC1_ genes have high levels of similarity (above 57%) with their counterparts in *S. enterica* (McClelland et al., 2001), *P. aeruginosa* (McPhee et al., 2003), and *D. dadantii* (Costechareyre et al., 2013) (Table S3). In addition, the *arn*_EC1_ operon also shares a conserved genetic organization with the *arn* operon (the *pmr* operon) in *S. enterica* (Figure 1a) (Gunn et al., 2000). Reverse transcription‐PCR (RT‐PCR) results indicated that these seven genes were cotranscribed in a way similar to their homologues in *S. enterica* (Figure 1b) (Gunn et al., 2000). In *S. enterica*, the *arn* operon confers bacterial resistance to polymyxin B, which also acts by damaging the bacterial cell envelope, similar to zeamines (Masschelein, Clauwers, Stalmans, et al., 2015). To further elucidate the roles of the *arn*_EC1_ operon, in‐frame deletion mutants of *arnBCADT*_EC1_ were generated in the background of the mutant ∆*zmsA*. MIC assay results showed that inactivation of these *arn*_EC1_ operon genes resulted in an over 8‐fold decrease in the MIC of zeamines and an over 300‐fold decrease in the MIC of polymyxin B (Table 1). Conversely, in trans expression of the corresponding *arn*_EC1_ genes, except for *arnE*_EC1_ and *arnF*_EC1_, in the mutants could fully restore the MICs of zeamines and polymyxin B (Table 1). These findings established the key roles of the *arn*_EC1_ operon in conferring zeamine and polymyxin B resistance in *D*. *oryzae*.

To further validate the role of the *arn*_EC1_ operon in LPS modification and thus resistance to CAMPs, the *arn*_EC1_ operon from *D*. *oryzae* EC1 was cloned and introduced into *D. dadantii* 3937, a zeamine‐ and polymyxin B‐sensitive strain (Table 1), together with the *ugd*_EC1_ gene (NCBI accession no. W909_RS06165), whose homologue in *E*. *coli* was found to be epistatic to the *arn* operon for lipid A modification (Yan et al., 2007). MIC assay results indicated that heterologous expression of the *arn*_EC1_ operon and the *ugd*_EC1_ gene in *D. dadantii* 3937 could increase bacterial resistance to zeamines and particularly polymyxin B (Table 1). These findings suggest that the *arn*_EC1_ operon from *D*. *oryzae* EC1 could effectively modify and reinforce the cell envelope of *D. dadantii* 3937, conferring strong resistance to the CAMP polymyxin B as well as zeamines.

Our previous study unveiled that the RND efflux pump DesABC plays a key role in zeamine resistance in *D. oryzae* EC1. Inactivation of *desB* resulted in an about eightfold decrease in the MIC of zeamines (Liang et al., 2019). Similarly, inactivation of *arnB*_EC1_ in the *zmsA* mutant background resulted in a 16‐fold decrease in the MIC of zeamines (Table 1). To test the synergistic effect of the DesABC efflux pump and the Arn LPS modification system, we generated a triple deletion mutant, ∆*zmsA*∆*arnB*_EC1_∆*desB*, which showed a 128‐fold decrease in the MIC of zeamines compared to the parental strain ∆*zmsA* (Table 1). Significantly, double deletion of *arnB*_EC1_/*desB* brought down the MIC to the level of the zeamine‐sensitive *E. coli* DH5α (Table 1), suggesting that the DesABC efflux pump and the Arn LPS modification system are dominant self‐protection mechanisms of *D. oryzae* against its self‐produced zeamines.

### 2.3 Expression of the *arn*_EC1_ operon is growth‐dependent but zeamine‐independent

**FIGURE 2:**
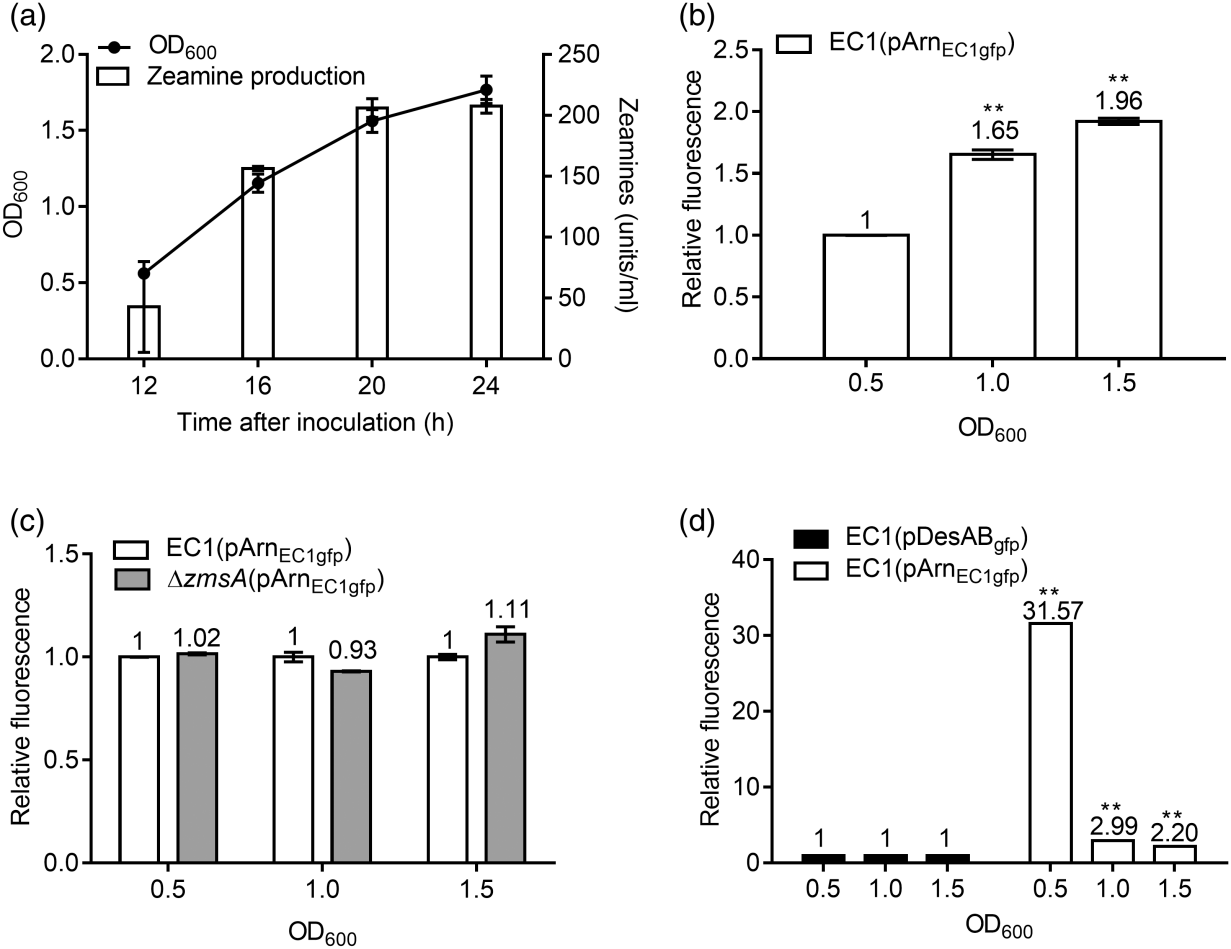
Expression pattern of the *arn*_EC1_ operon. (a) Bacterial growth and zeamine production of *Dickeya oryzae* EC1 in LS5 medium. The cell‐free supernatants of strain EC1 were collected and sterilized by filtration for the zeamine production assay. (b) The relative fluorescence of strain EC1(pArn_EC1gfp_) at different cell densities as indicated. The value at OD_600_ = 0.5 was used for normalization. (c) The relative fluorescence of strains EC1(pArn_EC1gfp_) and ∆*zmsA*(pArn_EC1gfp_) at different cell densities as indicated. The value of strain EC1(pArn_EC1gfp_) at each cell density was used for normalizing the value of ∆*zmsA*(pArn_EC1gfp_) at the same cell density. (d) The relative fluorescence of strains EC1(pDesAB_gfp_) and EC1(pArn_EC1gfp_) at different bacterial cell densities. The value of strain EC1(pDesAB_gfp_) at each cell density was used for normalizing the value of EC1(pArn_EC1gfp_) at the same cell density. Data are presented as mean ± standard error (*n* = 3). Statistical analysis was performed using a two‐tailed unpaired Student's *t* test versus strain EC1(pArn_EC1gfp_) at OD_600_ = 0.5 (b) or strain EC1(pDesAB_gfp_) at each growth stage (d). **p < 0.01.

Our previous work indicated that expression of *desAB* is stimulated by zeamines (Liang et al., 2019). To test whether expression of the *arn*_EC1_ operon is also zeamine‐dependent, the promoter region of the *arn*_EC1_ operon was fused with the *gfp*‐coding region in plasmid pPROBE‐NT (Miller et al., 2000) to generate the reporter construct pArn_EC1gfp_. The relative fluorescence of the wild‐type strain EC1 and the zeamine‐minus mutant ∆*zmsA* harbouring the pArn_EC1gfp_ construct, that is, EC1(pArn_EC1gfp_) and ∆*zmsA*(pArn_EC1gfp_), was determined in LS5 medium, which was optimized for zeamine production (Liao et al., 2014). The results showed that the relative fluorescence of EC1(pArn_EC1gfp_) increased along with the growth of *D*. *oryzae* EC1 (Figure 2a,b), suggesting that expression of the *arn*_EC1_ operon is growth‐dependent. In addition, the relative fluorescence of EC1(pArn_EC1gfp_) and ∆*zmsA*(pArn_EC1gfp_) was comparable at different bacterial growth stages (Figure 2c), suggesting that unlike *desAB*, whose expression could be triggered by zeamines (Liang et al., 2019), expression of the *arn*_EC1_ operon is independent of the exposure of zeamines. Moreover, the relative fluorescence of EC1(pArn_EC1gfp_) was higher than the relative fluorescence of EC1(pDesAB_gfp_), a *desAB* reporter strain for determining *desAB* expression in the wild‐type strain EC1 (Liang et al., 2019), at different bacterial growth stages, especially at the early one when zeamines were not highly accumulated (Figure 2a,d), suggesting that the *arn*_EC1_ genes had higher expression levels than *desAB* even when *D*. *oryzae* produced a small amount of zeamines. These findings suggest that the Arn_EC1_ LPS modification system is a zeamine‐independent resistance mechanism crucial to the self‐resistance of *D*. *oryzae*.

### 2.4 The *arn*_EC1_ mutants show varied levels of bacterial virulence

**FIGURE 3:**
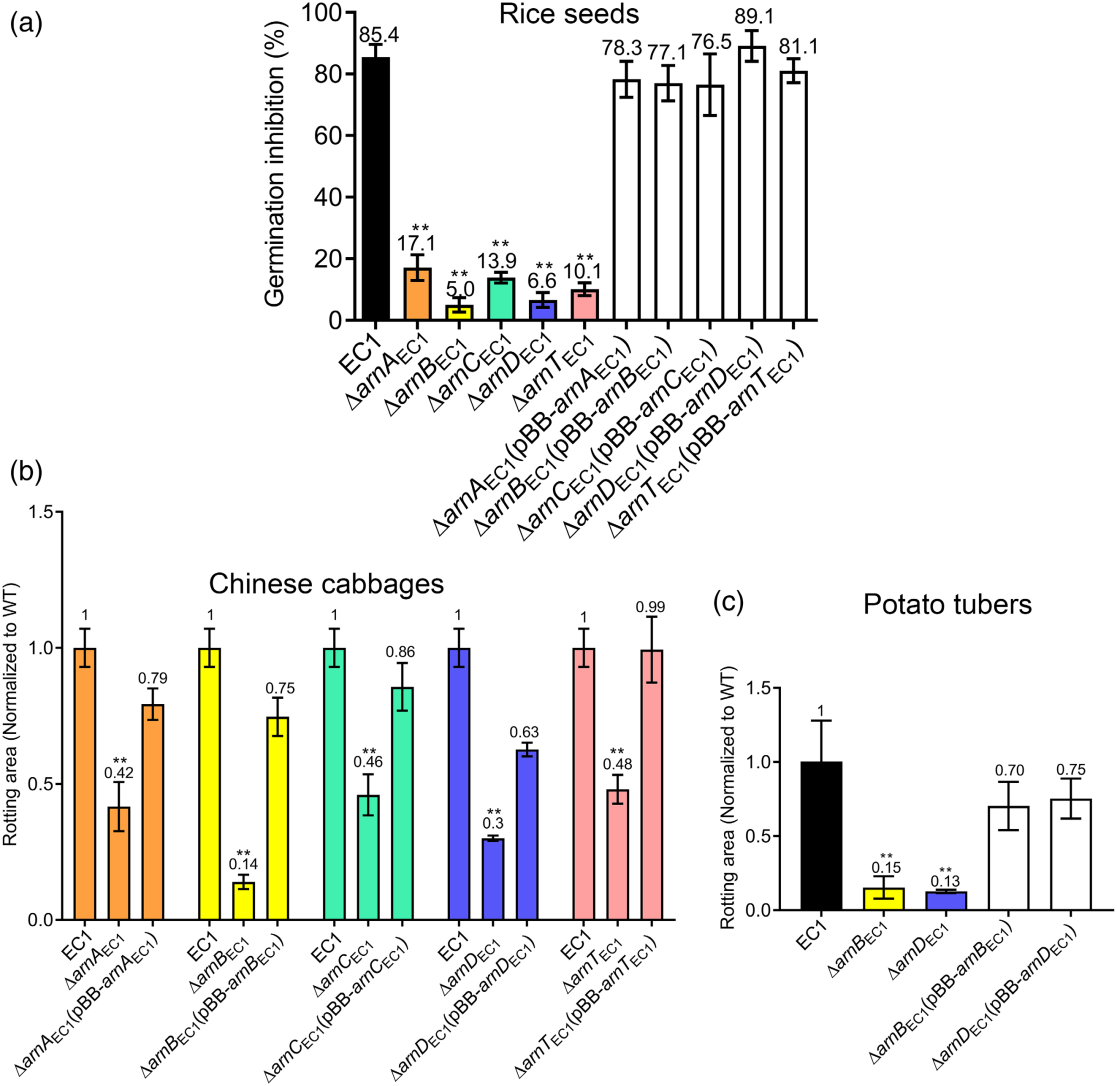
Virulence assay of *Dickeya oryzae* EC1 and derivatives on rice seeds, Chinese cabbage, and potato tubers. (a) Virulent effects of bacteria on rice seed germination. Bacterial cells cultured in Luria‐Bertani medium at the exponential phase were collected and resuspended to OD_600_ = 1.0 with double‐distilled water, and 10^8^‐fold dilutions were prepared using double‐distilled water. The rice seed germination rate was determined at 5 days postinoculation. (b) Bacterial virulence against Chinese cabbage. (c) Bacterial virulence against potato tubers. In (b) and (c), 2 μL of bacterial suspension with OD_600_ = 1.0 was inoculated onto Chinese cabbages or potato tubers. The rotting areas were measured at 36–48 h postinoculation. In (a–c), data are presented as mean ± standard deviation (*n* = 3). Statistical analysis was performed using a two‐tailed unpaired Student's *t* test versus strain EC1 (wild type, WT). **p < 0.01.

Given that the Arn_EC1_ genes are involved in bacterial LPS modification, we inferred that the virulence of their mutants might be somehow compromised in the process of pathogen–host interaction. Following this thought, the virulence of *arn*_EC1_ in in‐frame deletion mutants was assayed against rice seeds, Chinese cabbage, and potato tubers. The results showed that compared to the wild‐type strain EC1, the virulence of the five single mutants with one of the *arnBCADT*_EC1_ genes deleted was attenuated in inhibition of rice seed germination when challenged with 10^8^‐fold dilutions of cell resuspensions (Figure 3a). Similarly, these mutants were also less virulent on Chinese cabbage (Figure 3b). Intriguingly, however, we found that compared to the single mutants in the *arnCAT*_EC1_ genes, mutants ∆*arnB*_EC1_ and ∆*arnD*_EC1_ were much less virulent against rice seeds, Chinese cabbage, and potato tubers (Figures 3 and S1). In particular, deletion of *arnB*_EC1_ eliminated the most virulence of strain EC1 compared to the *arnCADT*_EC1_ single mutants in Chinese cabbage (Figure 3b). In trans expression of the corresponding *arn*_EC1_ genes in the mutants could restore bacterial virulence (Figure 3). Given these findings, we reasoned that in addition to the role of the *arn*_EC1_ operon in the bacterial LPS modification and hence the contribution to the in planta survival and virulence of *D. oryzae* EC1, *arnB*_EC1_ and *arnD*_EC1_ might play additional roles in modulation of *D. oryzae* physiology and virulence.

### 2.5 The Arn_EC1_ system is negatively associated with the bacterial mucoid morphotype and positively involved in biofilm formation

**FIGURE 4:**
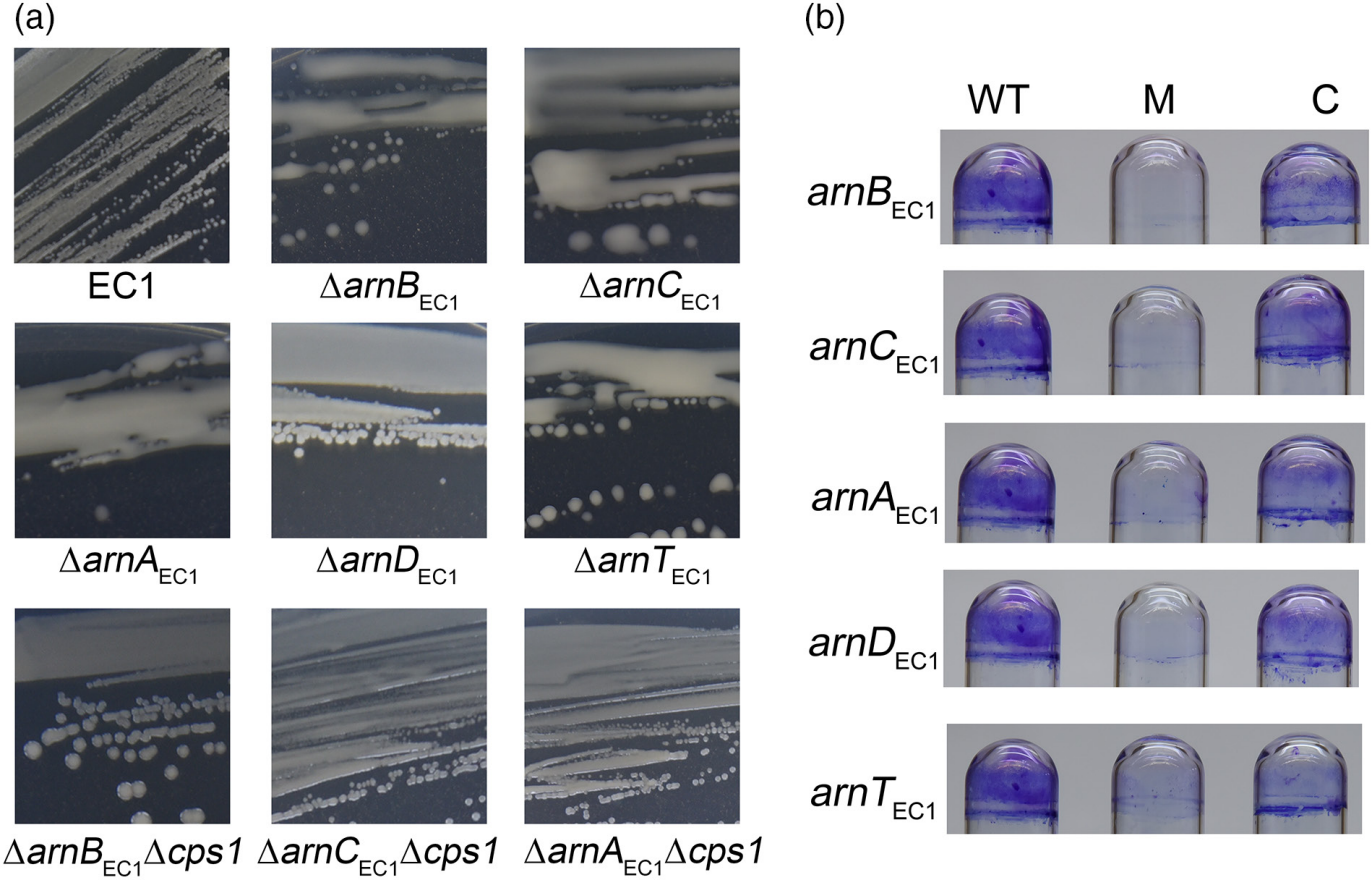
The *arn*_EC1_ genes are required for the bacterial nonmucoid morphotype and biofilm formation. (a) The morphotype of bacterial strains cultured on minimal medium (MM) agar plates supplemented with 5% (wt/vol) sucrose. (b) Mutation of the *arn*_EC1_ genes abolished biofilm formation. WT, *Dickeya oryzae* wild‐type strain EC1; M, mutant; C, complementation strain.

During the construction of *arn*_EC1_ in‐frame deletion mutants, we noticed that inactivation of any of the *arnBCAT*_EC1_ genes resulted in a phenotypic conversion from nonmucoid to mucoid when bacterial strains were cultured on minimal medium (MM) agar plates supplemented with 5% (wt/vol) sucrose (Figure 4a), which could be rescued by in trans expression of corresponding *arn*_EC1_ genes in the mutants (Figure S2). In contrast, deletion of *arnD*_EC1_ did not seem to affect bacterial morphology (Figures 4a and S2). A bacterial mucoid phenotype is often associated with the overproduction of capsular polysaccharides (CPSs) and biofilm formation in bacteria (Mann & Wozniak, 2012). Consistent with their potential roles in LPS modification and CPS production, we showed that the *arn*_EC1_ genes are involved in biofilm formation, a virulence‐related trait of *D. oryzae* EC1 (Chen et al., 2020; Lv et al., 2018, 2022; Shi et al., 2019; Zhou et al., 2016). Inactivation of any of the *arnBCAT*_EC1_ genes abolished the biofilm formation of *D. oryzae* EC1 (Figure 4b). Conversely, expression of the corresponding gene in trans could restore biofilm formation (Figure 4b). These findings support the notion that the Arn_EC1_ enzymes might collectively and separately contribute to the physiology and virulence of *D. oryzae*.

### 2.6 The *arn*_EC1_ genes play different roles in bacterial growth and swimming motility

**FIGURE 5:**
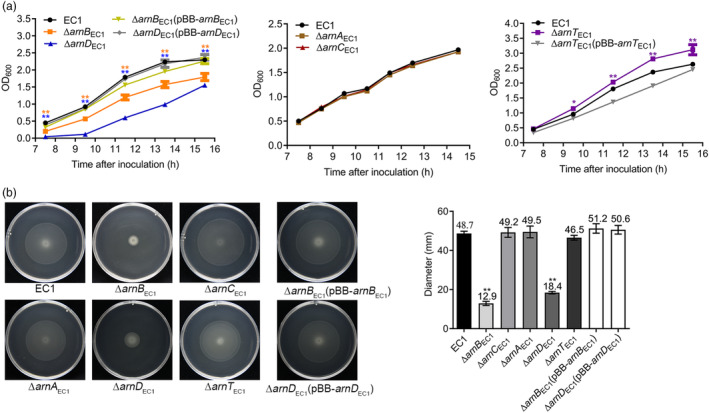
The roles of *arn*_EC1_ genes in bacterial growth and swimming motility. (a) Growth patterns of *Dickeya oryzae* EC1, the *arn*_EC1_ mutants, and the complementation strains. Statistical analysis was performed using a two‐tailed unpaired Student's *t* test versus strain EC1. The statistical analysis results are shown at different time points above the growth curves, with the colours corresponding to the growth curves of the *arn*_EC1_ mutants. *p < 0.05, **p < 0.01. (b) Swimming motility of strain EC1 and the *arn*_EC1_ mutants. Data are presented as mean ± standard deviation (*n* = 3). Statistical analysis was performed using a two‐tailed unpaired Student's *t* test versus strain EC1. **p < 0.01.

To further elucidate the roles of Arn_EC1_, we monitored the growth patterns of *arnBCADT*_EC1_ mutants in Luria–Bertani (LB) medium, in which *D*. *oryzae* EC1 is unable to produce a detectable level of zeamines (Liang et al., 2019). The results showed that compared to the mutants ∆*arnA*_EC1_ and ∆*arnC*_EC1_, which displayed a comparable growth pattern to the wild‐type strain EC1, the growth of mutants ∆*arnB*_EC1_ and ∆*arnD*_EC1_ was moderately or much retarded, respectively, whereas the mutant ∆*arnT*_EC1_ exhibited an increment in growth (Figure 5a). The swimming motility assay showed that inactivation of either *arnB*_EC1_ or *arnD*_EC1_ significantly reduced the diameters of chemotactic zones (Figure 5b). In trans expression of *arnBDT*_EC1_ genes in the mutants restored the growth of *arnBDT*_EC1_ mutants and the swimming motility of *arnBD*_EC1_ mutants to wild‐type levels (Figure 5).

**FIGURE 6:**
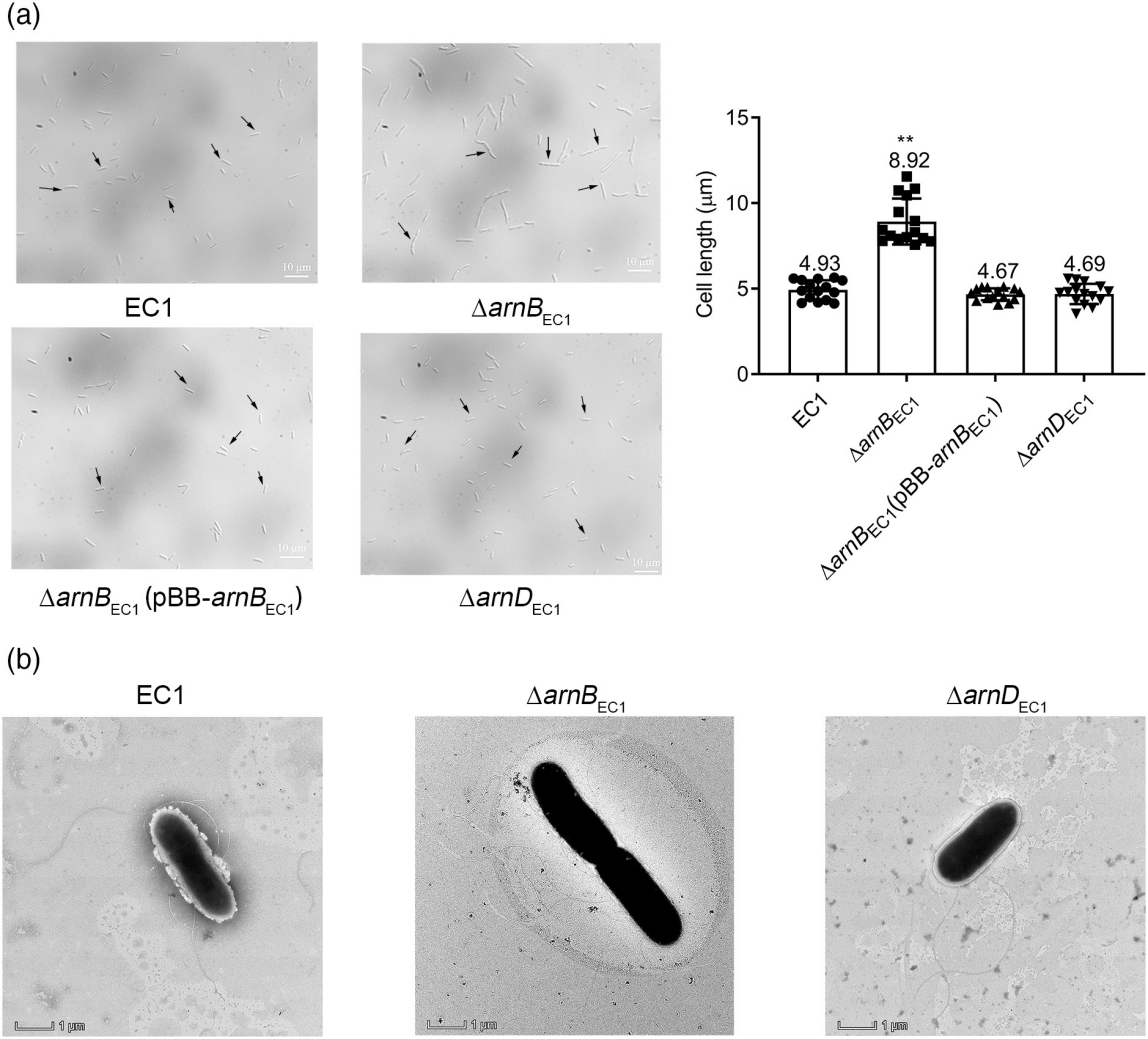
The role of *arnB*_EC1_ in bacterial cell division. (a) Morphology and cell length of *Dickeya oryzae* EC1, mutants ∆*arnB*_EC1_ and ∆*arnD*_EC1_, and the complementation strain ∆*arnB*_EC1_(pBB‐*arnB*_EC1_) as determined by optical microscopy. Data are presented as mean ± standard error (*n* = 15). Statistical analysis was performed using a two‐tailed unpaired Student's *t* test versus strain EC1. **p < 0.01. (b) Morphology of strain EC1 and mutants ∆*arnB*_EC1_ and ∆*arnD*_EC1_ as determined by transmission electron microscopy. The experiment was performed twice; representative images are shown in the figure.

To determine how ArnB_EC1_ and ArnD_EC1_ could affect bacterial growth and swimming motility, the cell morphology of mutants ∆*arnB*_EC1_ and ∆*arnD*_EC1_ was analysed by optical microscopy and transmission electron microscopy. The results unveiled that the cells of mutant ∆*arnB*_EC1_ appeared much longer than those of the wild‐type strain EC1 and mutant ∆*arnD*_EC1_, which was restored to the wild‐type level by in trans expression of *arnB*_EC1_ (Figure 6a). Further transmission electron microscopy analysis showed that cell division of ∆*arnB*_EC1_ was retarded (Figure 6b). These findings suggest that the compromised growth of ArnB_EC1_ null mutants might be related to its role in bacterial cell division. Intriguingly, however, deletion of *arnD*_EC1_ did not seem to affect bacterial cell division (Figure 6), although the growth of its mutant was more severely retarded than that of mutant ∆*arnB*_EC1_ (Figure 5a).

### 2.7 Transcriptomics analysis and functional study reveal the target genes regulated by ArnB_EC1_

To decipher the molecular mechanism by which ArnB_EC1_ modulates bacterial phenotypes beyond antibiotic resistance, transcriptomics analysis was performed to identify the differentially expressed genes in the wild‐type strain EC1 and mutant ∆*arnB*_EC1_ at the exponential growth phase (optical density at 600 nm [OD_600_] about 1.0 in LB medium). The results showed that inactivation of *arnB*_EC1_ led to significant changes in gene expression, with 291 genes being up‐regulated and 367 genes being down‐regulated (absolute log_2_[fold change] ≥1, Tables S4 and S5). In particular, a range of genes associated with phenotypic conversion between nonmucoid and mucoid, cell division, and swimming motility were found in the ArnB_EC1_ regulon.

**FIGURE 7:**
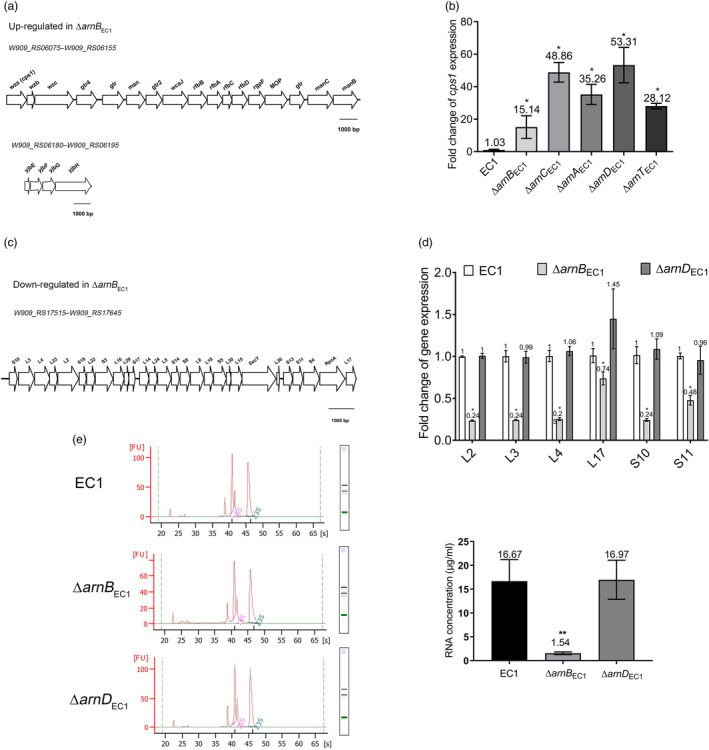
Transcriptomics analysis revealed the genes regulated by Arn_EC1_. (a) The genetic organization of the exopolysaccharide polysaccharide clusters up‐regulated in mutant ∆*arnB*_EC1_ compared to *Dickeya oryzae* EC1. (b) The relative transcript levels of *cps1* in strain EC1 and the *arn*_EC1_ mutants with the value of strain EC1 being used as the control. (c) The genetic organization of the S10 ribosomal protein gene cluster down‐regulated in mutant ∆*arnB*_EC1_ compared to strain EC1. (d) The relative transcript levels of S10 ribosomal genes in strain EC1 and mutants ∆*arnB*_EC1_ and ∆*arnD*_EC1_. (e) The profile of RNA extracted from strain EC1 and mutants ∆*arnB*_EC1_ and ∆*arnD*_EC1_. RNA integrity (left) and RNA concentration (right) were analysed. The RNA concentration was calculated by dividing the amount of RNA by the volumes of bacterial cell cultures used for RNA extraction. Data are presented as mean ± standard error (*n* = 4) (b, d) or mean ± standard deviation (*n* = 3) (e). Statistical analysis was performed using either the permutation test (b, d) or a two‐tailed unpaired Student's *t* test (e) versus strain EC1. *p < 0.05, **p < 0.01.

We firstly conducted detailed bioinformatics analyses on the 291 genes that were significantly up‐regulated in mutant ∆*arnB*_EC1_ (Table S4). The Kyoto Encyclopedia of Genes and Genomes (KEGG) pathway analysis showed that the O‐antigen nucleotide sugar biosynthesis pathway was significantly up‐regulated in mutant ∆*arnB*_EC1_ (*p*_adj_ ≤ 0.05), including the genes located in two *eps* (exopolysaccharide polysaccharide) clusters (Figure 7a, Table S4). One *eps* cluster (NCBI accession nos. W909_RS06075 to W909_RS06155) contains the homologues of the *wzabc* genes. In *E*. *coli*, the *wzabc* genes encode proteins involved in group I CPS transport (Dong et al., 2006). The other *eps* cluster contains the homologues of *yjbEFGH*. In *E*. *coli*, the *yjbEFGH* genes are responsible for the production of an unknown exopolysaccharide (Ferrieres et al., 2007). Our transcriptomics analysis results showed that the genes in these two *eps* clusters were up‐regulated at least 4‐fold in mutant ∆*arnB*_EC1_ (log_2_[fold change] ≥ 2, Table S4).

In *E*. *coli*, the mucoid phenotype is associated with the overproduction of CPSs (Wall et al., 2018). To address whether the mucoid phenotype of *arnBCAT*_EC1_ mutants is associated with the up‐regulation of the CPS genes found in the transcriptomics analysis, *cps1* expression in mutants ∆*arnB*_EC1_, ∆*arnC*_EC1_, ∆*arnA*_EC1_, and ∆*arnT*_EC1_ was compared to that in the wild‐type strain EC1 by reverse transcription‐quantitative PCR (RT‐qPCR) analysis. The results showed that compared to the wild‐type strain EC1, *cps1* expression was increased 15‐ to 48‐fold in these four mutants (Figure 7b). In‐frame deletion of *cps1* in the backgrounds of single mutants in the *arnBCA*_EC1_ genes led to conversion from a mucoid to a nonmucoid phenotype (Figures 4a and S2). These findings indicate that Arn_EC1_ proteins maintain the nonmucoid morphotype of *D. oryzae* EC1 through repression of CPS production.

We then analysed the 367 genes down‐regulated in the mutant ∆*arnB*_EC1_ (Table S5). KEGG pathway analysis indicated that the ribosome pathway was significantly affected in mutant ∆*arnB*_EC1_ (*p*_adj_ ≤ 0.05, Table S5). In particular, the genes in the S10 ribosomal protein gene cluster (NCBI accession nos. W909_RS17515 to W909_RS17645) were significantly down‐regulated (Figure 7c, Table S5). Previous studies with *E. coli* and *Bacillus subtilis* unveiled that the genes in the S10 ribosomal protein gene cluster, that is, L2, L3, L17, and S11, are essential for bacterial growth, cell division, and swimming motility (Klitgaard et al., 2015; Suzuki et al., 2014; Takada et al., 2014; Zouine et al., 2000), which are the phenotypes modulated by ArnB_EC1_ in *D. oryzae* EC1. Consistent with the transcriptomics analysis results, RT‐qPCR analysis results confirmed that the transcript levels of L2, L3, L4, L17, S10, and S11 in the S10 ribosomal protein gene cluster were significantly decreased in mutant ∆*arnB*_EC1_ compared to the wild‐type strain EC1 and mutant ∆*arnD*_EC1_ (Figure 7d), an *arn*_EC1_ mutant with defects in bacterial growth and swimming motility as mutant ∆*arnB*_EC1_ (Figure 5). In *E*. *coli*, RNA biosynthesis is closely linked to the production of ribosomal proteins (Zengel & Lindahl, 1994). We demonstrated that although RNA integrity in mutant ∆*arnB*_EC1_ was comparable to that in the wild‐type strain EC1 and mutant ∆*arnD*_EC1_ (Figure 7e), mutant ∆*arnB*_EC1_ had a lower amount of RNA than the wild‐type strain EC1 and mutant ∆*arnD*_EC1_ (Figure 7e). These findings seem to suggest that ArnB_EC1_ affects bacterial growth, cell division, and swimming motility through regulating a specific pathway to maintain homeostasis of RNA biosynthesis and expression of S10 ribosomal genes in *D*. *oryzae*.

## 3 DISCUSSION

Zeamines are phytotoxins produced by the phytopathogen *D. oryzae* that play vital roles as key virulence determinants in bacterial pathogenesis (Cheng et al., 2013; Zhou et al., 2011). In addition, these polyamine compounds are also potent antibiotics against fungi, nematodes, and a range of bacterial pathogens (Hellberg et al., 2015; Liao et al., 2015; Masschelein, Clauwers, Awodi et al., 2015; Wu et al., 2010). The zeamine producer *D*. *oryzae* presents a remarkable higher level of zeamine resistance than a range of bacterial pathogens (Liang et al., 2019; Wu et al., 2010). Obviously, such a superior self‐protection mechanism would be of critical importance for the bacterium to become a formidable pathogen and habitat dominator, as it allows the bacteria to produce sufficient phytotoxins/antibiotics to compete against the host and other microorganisms. In this study, we documented a new zeamine resistance mechanism encoded by the *arn*_EC1_ operon that confers on *D. oryzae* an ability to protect itself against the toxicity of zeamines. Inactivation of the *arn*_EC1_ operon genes drastically decreased *D. oryzae* EC1 resistance to zeamines (Table 1). In addition, significantly, we found that the *arn*_EC1_ genes also play roles in modulation of CPS production (Figures 4a and S2), cell division (Figure 6), and bacterial growth (Figure 5a), thus influencing certain virulence‐related traits, including motility (Figure 5b), biofilm formation (Figure 4b), and pathogenesis (Figure 3). This study thus deciphers a new zeamine resistance mechanism and expands our understanding on the biological roles and significance of the Arn_EC1_ system in microbial pathogens.

The *arn* operon is known for its role in bacterial resistance against CAMPs such as polymyxin B (Gunn et al., 2000; Jiang et al., 2010; Lin et al., 2014; McPhee et al., 2003; Winfield et al., 2005). The operon contains seven genes encoding the enzymes for modification of bacterial outer membrane LPS with the cationic l‐Ara4N moiety, which reduces the net negative charge of LPS and limits the binding, accumulation, and permeation of CAMPs (Yan et al., 2007). Among these seven genes, *arnA* encodes a decarboxylase/UDP‐4‐amino‐4‐deoxy‐l‐arabinose formyltransferase catalysing the first reaction step for the biosynthesis of UDP‐4‐ketopentose from UDP‐glucuronic acid. A UDP‐4‐amino‐4‐deoxy‐l‐arabinose‐oxoglutarate aminotransferase encoded by *arnB* converts UDP‐4‐ketopentose to UDP‐β‐l‐Ara4N at the second reaction step. At the third reaction step, an undecaprenyl phosphate‐4‐deoxy‐4‐formamido‐l‐arabinose transferase encoded by *arnC* transfers 4‐deoxy‐4‐formamido‐l‐arabinose from UDP to undecaprenyl phosphate for ArnD deformylation to form undecaprenyl phosphate‐α‐l‐Ara4N. The undecaprenyl phosphate‐α‐l‐Ara4N may be transported from the inner membrane to the outer membrane by two l‐Ara4N‐phosphoundecaprenol flippase subunits, ArnE and ArnF. At the last step, an undecaprenyl phosphate‐α‐4‐amino‐4‐deoxy‐l‐arabinose arabinosyl transferase encoded by *arnT* transfers l‐Ara4N from undecaprenyl phosphate‐α‐l‐Ara4N to lipid A (Breazeale et al., 2005; Trent et al., 2001; Yan et al., 2007). Bioinformatics analysis showed that the Arn_EC1_ enzymes are highly similar to the well‐characterized Arn homologues in *S. enterica*, *P. aeruginosa*, and *D. dadantii* with an amino acid similarity of over 57% (Table 1) and proposed conserved functions. MIC assay results unveiled the involvement of *arn*_EC1_ genes in *Dickeya* tolerance against zeamines and polymyxin B (Table 1). Similar to polymyxin B, zeamines also contain positive charged amino groups and act by damaging the cell envelope of gram‐negative bacteria (Masschelein, Clauwers, Stalmans, et al., 2015), implying that addition of the l‐Ara4N moiety in LPS by the Arn_EC1_ system could reduce the chance of zeamines damaging the bacterial cell membrane. In addition, we found that the Arn_EC1_ system plays an essential role in *D*. *oryzae* biofilm formation, which is a well‐established antimicrobial and stress tolerance mechanism (Flemming et al., 2016). A previous study showed that inactivation of an *arnA*_EC1_ homologue, *pmrI*, led to partially decreased biofilm formation in *P*. *mirabilis* (Jiang et al., 2010). In the present study, we found that biofilm formation in the *arn*_EC1_ deletion mutants of *D. oryzae* was almost completely abolished (Figure 4b). Previous studies underlined the positive contributions of LPS to two key steps of biofilm formation, namely, bacterial initial attachment and adhesion to the abiotic surface (Ruhal & Kataria, 2021), and a general negative impact of CPS overproduction on biofilm formation (Limoli et al., 2015). As inactivation of the *cps1* gene did not restore biofilm formation in *arn*_EC1_ mutants, we proposed the complete abolishment of biofilm formation resulted from the negative impact of LPS changes after inactivation of *arn*_EC1_ genes, but not a general negative influence of CPS overproduction caused by inactivation of *arn*_EC1_ genes. Taken together, we conclude that the Arn_EC1_ system confers zeamine and CAMP resistance through modification of LPS and facilitation of biofilm formation in *D. oryzae* EC1.

We recently reported that the RND efflux pump DesABC is involved in zeamine resistance (Liang et al., 2019). Interestingly, null mutation of the DesABC pump in *D. oryzae* EC1 causes an about eightfold decrease in zeamine resistance (Liang et al., 2019). In addition, transcriptional expression of *desAB* is inducible and triggered by exogenous addition of zeamines but not polymyxin B (Liang et al., 2019). In contrast, however, transcriptional expression of the *arn*_EC1_ genes appeared to be cell density‐dependent and was not affected by zeamines (Figure 2a–c), and deletion of *arn*_EC1_ genes caused a more severe loss of resistance to polymyxin B than to zeamines (Table 1). These findings suggest that DesABC is a zeamine‐specific resistance mechanism, whereas Arn_EC1_ is more likely a broad‐spectrum resistance mechanism against various CAMPs produced by the hosts and the competitors of *D*. *oryzae* in the environment. Such high expression of the Arn_EC1_ system at the early bacterial growth stage may facilitate *D. oryzae* survival upon exposure to CAMPs from the hosts and in the presence of competitors of *D. oryzae* when zeamines are not highly produced and accumulated for competition in the environment. Importantly, we found that simultaneous knockdown of *arnB*_EC1_ and *desB* decreased the zeamine resistance of *D. oryzae* EC1 to a level similar to that of the zeamine‐sensitive *E*. *coli* DH5α (Table 1), indicating that the DesABC efflux pump and the Arn_EC1_ LPS modification system are the predominant self‐protection mechanisms of *D. oryzae* against its self‐produced toxic zeamines.

In addition to validation of the role of the Arn_EC1_ LPS system in CAMP resistance and biofilm formation (Figure 4b, Table 1), which have been documented in other bacterial species as well (Gunn et al., 2000; Jiang et al., 2010), interestingly, the results from this study unveiled several new biological functions associated with the Arn_EC1_ system or its components. Transcriptomics analysis performed between the wild‐type strain EC1 and the *arnB*_EC1_ mutant not only suggested the bacterial growth and virulence conferred by ArnB_EC1_ are associated with the contribution of ArnB_EC1_ to the regulation of the expression of hundreds of genes in *D*. *oryzae*, especially those belonging to ribosome, carbon metabolism, and oxidative phosphorylation pathways, but also unveiled the similarities and dissimilarities of Arn_EC1_ components in regulating biological functions. Firstly, ArnBCAT_EC1_ have similar roles in conferring a nonmucoid phenotype through repressing CPS genes. Transcriptomics analysis results unveiled that inactivation of *arnB*_EC1_ resulted in overexpression of CPS genes (Table S4), which subsequently led to the finding that ArnCAT_EC1_ also influenced the conversion between mucoid and nonmucoid phenotypes through modulation of CPS gene expression (Figures 4a, 7b, and S2). Notably, ArnD_EC1_ plays a dissimilar role in conferring the bacterial nonmucoid morphotype. Inactivation of *arnD*_EC1_ up‐regulated CPS gene expression (Figure 7b), but this did not result in a mucoid phenotype as inactivation of the *arnBCAT*_EC1_ genes (Figure 4). We proposed that in addition to a similar role in the regulation of CPS gene expression, ArnD_EC1_ may play additional roles in modulating metabolic pathways required for the biosynthesis of CPS components. This was evident as ArnD_EC1_ had a higher contribution to bacterial growth than other Arn_EC1_ components (Figure 5a). Such multifaceted impact of ArnD_EC1_ on bacterial metabolism may lead to the difference between ArnD_EC1_ and other Arn_EC1_ components in regulating the conversion between mucoid and nonmucoid phenotypes. Secondly, it is intriguing to find that although ArnB_EC1_ and ArnD_EC1_ have regulatory roles in bacterial growth and swimming motility, ArnB_EC1_ and ArnD_EC1_ have distinct regulatory mechanisms. Transcriptomics analysis results unveiled that ArnB_EC1_ induces the expression of S10 ribosomal genes, whose counterparts in *E. coli* and *B. subtilis* are involved in bacterial growth, cell division, and swimming motility (Klitgaard et al., 2015; Suzuki et al., 2014; Takada et al., 2014; Zouine et al., 2000). However, unlike ArnB_EC1_, ArnD_EC1_ did not play a significant role in inducing the expression of S10 ribosomal genes and a process associated with ribosomal protein production, that is, RNA biosynthesis (Figure 7d,e). We proposed these observations may result from the fact that ArnB_EC1_ and ArnD_EC1_ have different substrate specificity, as ArnB_EC1_ is a proposed putative UDP‐4‐amino‐4‐deoxy‐l‐arabinose‐oxoglutarate aminotransferase, whereas ArnD_EC1_ is a proposed 4‐deoxy‐4‐formamido‐l‐arabinose‐phosphoundecaprenol deformylase. Although the mechanisms of action of ArnB_EC1_ and ArnD_EC1_ are still unclear, studies of the biological functions of *arnB*_EC1_ and *arnD*_EC1_ mutants provided useful clues on how ArnB_EC1_ and ArnD_EC1_ affect the virulence of *D. oryzae*. The present study demonstrated the roles of ArnB_EC1_ and ArnD_EC1_ in the regulation of swimming motility and bacterial growth (Figure 5). Bacterial motility is one of the key virulence determinants of *D. oryzae* that plays important roles in bacterial invasion and systemic infection (Chen et al., 2020, 2022; Hussain et al., 2008; Lv et al., 2018, 2022; Shi et al., 2019; Zhou et al., 2016). It seems rational that the larger contributions of ArnB_EC1_ and ArnD_EC1_ than other Arn_EC1_ components to *D. oryzae* virulence resulted from the positive regulation of swimming motility by ArnB_EC1_ and ArnD_EC1_. Given that ArnB_EC1_ and ArnD_EC1_ have different regulatory roles in cell division, expression of S10 ribosomal genes, and RNA synthesis (Figures 6 and 7d,e), we proposed the contributions of ArnB_EC1_ and ArnD_EC1_ to *D. oryzae* virulence are redundant.

In summary, this study unveils a new zeamine resistance mechanism mediated by the Arn_EC1_ LPS modification system in *D*. *oryzae* EC1; this mechanism and the previously identified DesABC efflux pump constitute the foremost resistance mechanisms against zeamines. These findings improve our understanding of how *D. oryzae* could safeguard itself while producing formidable toxic molecules to infect host organisms and attack its potential competitors and may facilitate engineering of *D. oryzae* strains able to overproduce this family of potent antimicrobial compounds. In addition, importantly, characterization of the Arn_EC1_ system led to the discovery of new roles of the Arn_EC1_ system and its components in bacterial physiology and virulence, which provide not only valuable insight into the biological functions of the widely conserved Arn system, but also useful clues for further elucidating the complicated and sophisticated regulatory mechanisms that govern bacterial physiology and virulence.

## 4 EXPERIMENTAL PROCEDURES

### 4.1 Bacterial strains, plasmids, primers, and growth conditions

The bacterial strains and plasmids used in this study are listed in Table S1. Primers used in this study are listed in Table S2. *D. oryzae* and *D. dadantii* derivatives were routinely grown at 28°C in LB medium, MM medium, or LS5 medium described previously (Liang et al., 2019). *E. coli* derivatives were grown at 37°C in LB medium. Antibiotics were added when necessary at the following concentrations: ampicillin, 50 μg/mL; chloramphenicol, 50 μg/mL; gentamycin, 50 μg/mL; kanamycin, 50 μg/mL; streptomycin, 50 μg/mL; polymyxin B, 50 μg/mL.

### 4.2 Construction of in‐frame deletion, complementation, and heterologous expression strains

Construction of in‐frame deletion and complementation strains was performed following a previously described method (Liang et al., 2019). Briefly, for in‐frame gene deletion, a suicide plasmid constructed from pKNG101 with the flanking regions of each *arn*_EC1_ gene was transformed into the wild‐type strain EC1 or the mutant ∆*zmsA* by triparental mating. The desired in‐frame deletion mutants were screened on MM agar plates supplemented with 5% (wt/vol) sucrose and confirmed by PCR and DNA sequencing. For constructing complementation strains, each *arn*_EC1_ gene was cloned into the low‐copy plasmid pBBR1‐MCS4 and constitutively expressed under the control of the *lac* promoter in pBBR1‐MCS4. The resultant construct was transformed into the corresponding mutant and the desired strains were screened on MM agar plates with ampicillin and verified by PCR. Construction of the plasmid pAmob‐*arnBCADTEF*_EC1_ for heterologous expression of the *arn*_EC1_ operon in *D. dandantii* 3937 was performed following a previously described method (Fu et al., 2012). Briefly, an 8‐kb DNA fragment containing the open reading frame of *arnBCADTEF*_EC1_ was directly cloned from the digested genomic DNA of the wild‐type strain EC1 and recombined with the linearized pACYC184 by the RecET‐Redγ recombination system in *E. coli* GB05‐dir. The resultant construct pA‐*arnBCADTEF*_EC1_ was introduced with the *mob* region cloned from pBBR1‐MCS4 for DNA transfer. The plasmids pAmob‐*arnBCADTEF*_EC1_ and pBB‐*ugd*_EC1_ were cotransformed into *D. dadantii* 3937 for heterologous expression of *arnBCADTEF*_EC1_ and *ugd*_EC1_.

### 4.3 Transposon mutagenesis analysis

To identify the genes that are involved in zeamine resistance in *D. oryzae* EC1, the mutant ∆*zmsA* was randomly mutated with the mariner‐based transposon carried by pBT20 (Seet & Zhang, 2011) through biparental mating on YEB agar plates (10 g/L tryptone, 5 g/L yeast extract, 10 g/L KCl, 10 g/L sucrose, 0.5 g/L MgSO_4_⸱7H_2_O, 18 g/L agar, pH 7.0). Zeamine sensitivity of each mutant was monitored by determining its growth in LB medium containing zeamines at half the MIC of zeamines for the mutant ∆*zmsA* (900 μg/mL) in 96‐well plates. The mutants showing no visible growth after overnight culturing in LB medium with zeamines were considered as the desired mutants. To localize the transposon insertion sites in the desired mutants, the flanking regions of transposon insertion sites were amplified by FPNI‐PCR (Wang et al., 2011).

### 4.4 Preparation of zeamines

Zeamines used in this study were purified with the absorbent resin XAD7 (Sigma‐Aldrich) and confirmed by liquid chromatography–mass spectrometry (LC–MS) following a previously described method (Liang et al., 2019). Briefly, cell supernatants of strain EC1 cultured in LS5 medium were passed through a column with the XAD7 resin. Double‐distilled water and methanol were used to wash the column, and crude zeamines were eluted from the column by acetone. Zeamine confirmation was performed by identification of zeamine, zeamine I, and zeamine II using LC–MS.

### 4.5 MIC assay

The MIC assay was performed using the previously described broth microdilution method (Liang et al., 2019) following the recommendations of the Clinical and Laboratory Standards Institute. The MIC of zeamines or polymyxin B for bacterial strains was defined as the lowest antibiotic concentration with no visible cell growth.

### 4.6 Virulence against rice, Chinese cabbage, and potato

Determination of bacterial pathogenicity against rice seeds, Chinese cabbage, and potato tubers was performed following previously described methods (Zhou et al., 2011) with minor modifications. Briefly, in the rice seed germination assay, bacterial cells cultured in LB medium at the exponential phase were collected, resuspended in double‐distilled water, and adjusted to an OD_600_ of 1.0 before being diluted 10^8^‐fold in double‐distilled water. About 20 rice seeds of cultivar Nipponbare were treated with 10 mL of bacterial suspension at room temperature for 5 h. Rice seeds treated with double‐distilled water were used as the control. After a 5‐h treatment, rice seeds were washed with double‐distilled water twice and then transferred onto moistened filter papers in 10 × 10‐cm Petri dishes. Rice seeds were incubated at 28°C under 16/8 h light/dark conditions for 5 days. After incubation for 5 days, the rice seed germination rate was determined. In the Chinese cabbage and potato tuber assays, Chinese cabbages and potatoes bought from local shops were washed with double‐distilled water, dried, and then surface‐sterilized with 70% ethanol. Chinese cabbages were then sliced to 8 × 8‐cm pieces for inoculation. Potatoes were sliced evenly about 5 mm in thickness for inoculation. The sliced Chinese cabbages and potato tubers inoculated with bacterial suspensions in double‐distilled water (OD_600_ about 1.0) were incubated at 28°C for 36–48 h. After incubation, Chinese cabbages and potato tubers were photographed. The rotting areas caused by the wild‐type strain EC1 and *arn*_EC1_ mutants were determined by ImageJ software (Schneider et al., 2012) and then normalized to the rotting area caused by the wild‐type strain EC1.

### 4.7 Mucoid and nonmucoid morphotype analyses

Bacterial cultures at the exponential phase were streaked on MM agar plates supplemented with 5% (wt/vol) sucrose. After inoculation at 28°C for about 48 h, the plates were photographed.

### 4.8 Biofilm formation assay

The biofilm formation assay was performed following a previously described method (Chen et al., 2020) with minor modifications. Briefly, bacterial cultures at the exponential phase were adjusted to an OD_600_ of 1.0. The adjusted bacterial cultures were inoculated (0.1% vol/vol) into YEB medium in 10‐cm glass tubes. The glass tubes were then shaken at 28°C for 48 h. After incubation, bacterial cell cultures were removed and the biofilm mass stained on the glass tubes was washed twice with double‐distilled water. The biofilm mass was then stained with 0.1% (wt/vol) crystal violet for 15 min. After the staining process, unbound crystal violet was removed, and biofilm mass stained with crystal violet was rinsed three times with double‐distilled water. The biofilm mass stained with crystal violet was photographed after drying.

### 4.9 Green fluorescent protein transcriptional fusion assay

The reporter plasmid pArn_gfp_ was constructed from the pPROBE‐NT reporter plasmid (Miller et al., 2000). The *gfp* transcriptional fusion assay was performed following a previously described method (Liang et al., 2019). Briefly, the average fluorescence of 50,000 bacterial cells of each sample was measuring by a CytoFLEX flow cytometer (Beckman Coulter). The relative fluorescence of each bacterial strain was expressed as the fluorescence of the bacterial strain normalized to the fluorescence of the corresponding control.

### 4.10 Swimming motility assay

The swimming motility of *D*. *oryzae* strains was assayed in semisolid agar plates following a previously described method (Shi et al., 2019). Briefly, fresh bacterial cell cultures at an OD_600_ of 1.0 were spotted on semisolid agar plates (10 g/L bacteriological peptone, 5 g/L NaCl, 3 g/L agar, pH 7.0) and incubated at 28°C for 16–24 h. After incubation, the diameter of the chemotactic zone was measured.

### 4.11 Microscopy analysis

Fresh bacterial cell cultures at an OD_600_ of 1.0 were collected for optical microscopy and transmission electron microscopy analyses. For optical microscopy analysis, bacterial cells were fixed onto the slides by passing the slides through a flame and imaged using an Axio Observer Z1 microscope (Zeiss) equipped with a scientific complementary metal oxide semiconductor (sCMOS) camera (PCO Edge) in DIC mode. Fifteen individual cells from three different microscopic views were randomly selected for measurement of bacterial cell length using ZEN 2 (blue edition) v. 2.0 software.

For transmission electron microscopy analysis, a copper grid was floated on a drop of bacterial cell culture for 1 min. After the adsorption process, the copper grid was rinsed three times with double‐distilled water and then stained with TI blue (Nisshin EM Co., Ltd) for 1 min. Samples were imaged by Talos L120C (Thermo Fisher) and at least three representative cells of each sample were photographed.

### 4.12 RNA extraction, transcriptomics, RT‐PCR, and RT‐qPCR

Total RNA of bacterial cells was extracted with the SV Total RNA Isolated System kit (Promega). RNA density was measured by a NanoDrop 2000c spectrophotometer (Thermo Fisher Scientific), and RNA integrity was analysed using the RNA Nano 6000 Assay Kit and an Agilent 2100 Bioanalyzer system (Agilent Technologies). For transcriptomics analysis, the libraries were prepared using the TruSeq PE Cluster Kit v3‐cBot‐HS (Illumina) following the manufacturer's instructions and sequenced using an Illumina Novaseq platform. Two biological replicates were applied in this assay. Data were aligned by Bowtie v. 2‐2.2.3. Differential expression analysis was performed using the DESeq R package (v. 1.18.0), and genes with log_2_[fold change] ≥ |1| and adjusted *p* value ≤ 0.05 (*p*_adj_ ≤ 0.05) were considered as differentially expressed. KEGG pathway enrichment analysis was performed by testing the statistical enrichment of differentially expressed genes using KOBAS software. For RT‐PCR analysis, the intergenic regions of *arnBC*_EC1_, *arnCA*_EC1_, *arnAD*_EC1_, *arnDT*_EC1_, *arnTE*_EC1_, and *arnEF*_EC1_ were amplified for different samples using 2× EasyTaq PCR SuperMix (+dye) (TransGen Biotech) following the manufacturer's instructions. RT‐qPCR analysis was performed following an established method (Liang et al., 2019) using a QuantStudio 6 Flex system and PowerUp SYBR green master mix (Thermo Fisher Scientific), with the 16S rRNA gene serving as the endogenous reference. The RT‐qPCR conditions were as follows: 50°C for 2 min, 95°C for 2 min, and 40 cycles at 95°C for 15 s, 57°C for 15 s, and 72°C for 30 s. The fold change value of gene expression as determined by RT‐qPCR was calculated by the 2^−∆∆*Ct*^ method (Livak & Schmittgen, 2001).

### 4.13 Statistical analysis

All experiments were individually performed at least twice with three replicates each time. Statistical comparison was performed using the two‐tailed unpaired Student's *t* test in GraphPad Prism v. 7.0 software (GraphPad) or the permutation test for RT‐qPCR data as recommended (Pfaffl et al., 2002) in R software (v. 4.2.2). A *p* value of <0.05 was considered significant.

## Supporting information


**FIGURE S1** Virulence of *arnACT*_EC1_ mutants against potato tubers. Two microlitres of bacterial suspension in double‐distilled water (OD_600_ = 1.0) was inoculated in the sliced potato tubers. After incubation, the sliced potato tubers were photographed. The rotting areas caused by the wild‐type strain EC1 and *arnACT*_EC1_ mutants were determined by ImageJ software (Schneider et al., 2012). The rotting area caused by the wild‐type strain EC1 or *arnACT*_EC1_ mutants was normalized to that caused by the wild‐type strain EC1.

**FIGURE S2** The *arn*_EC1_ genes are required for the bacterial nonmucoid morphotype. The morphotype of bacterial strains cultured on minimal medium (MM) agar plates supplemented with 5% (wt/vol) sucrose. WT, the wild‐type strain EC1; M, mutant; C, complementation strain; M‐CPS, *arn*_EC1_/*cps1* double‐deletion mutant.

**TABLE S1** Bacterial strains and plasmids used in this study.

**TABLE S2** Primers used in this study.

**TABLE S3** Characteristics of the *arn*_EC1_ operon genes.

**TABLE S4** The up‐regulated genes in the *arnB*_EC1_ mutant compared to strain EC1.

**TABLE S5** The down‐regulated genes in the *arnB*_EC1_ mutant compared to strain EC1.

## Data Availability

The raw transcriptomics data have been deposited in the NCBI Sequence Read Archive at https://www.ncbi.nlm.nih.gov/sra under accession number PRJNA923730. Other data that support the findings of this study are provided in the supplementary information files.
