# Supplementary material for: Characterization of the Arn lipopolysaccharide modification system essential for zeamine resistance unveils its new roles in Dickeya oryzae physiology and virulence
Source: Mol Plant Pathol. 2023 Sep 22;24(12):1480–94. doi: 10.1111/mpp.13386 (PMC10632790; doi:10.1111/mpp.13386)
Supplement: Supplementary file 1 — FIGURE S1 Virulence of arnACT EC1 mutants against potato tubers. Two microlitres of bacterial suspension in double‐distilled water (OD600 = 1.0) was inoculated in the sliced potato tubers. After incubation, the sliced potato tubers were photographed. The rotting areas caused by the wild‐type strain EC1 and arnACT EC1 mutants were determined by ImageJ software (Schneider et al., 2012). The rotting area caused by the wild‐type strain EC1 or arnACT EC1 mutants was normalized to that caused by the wild‐type strain EC1. [file MPP-24-1480-s005.doc]

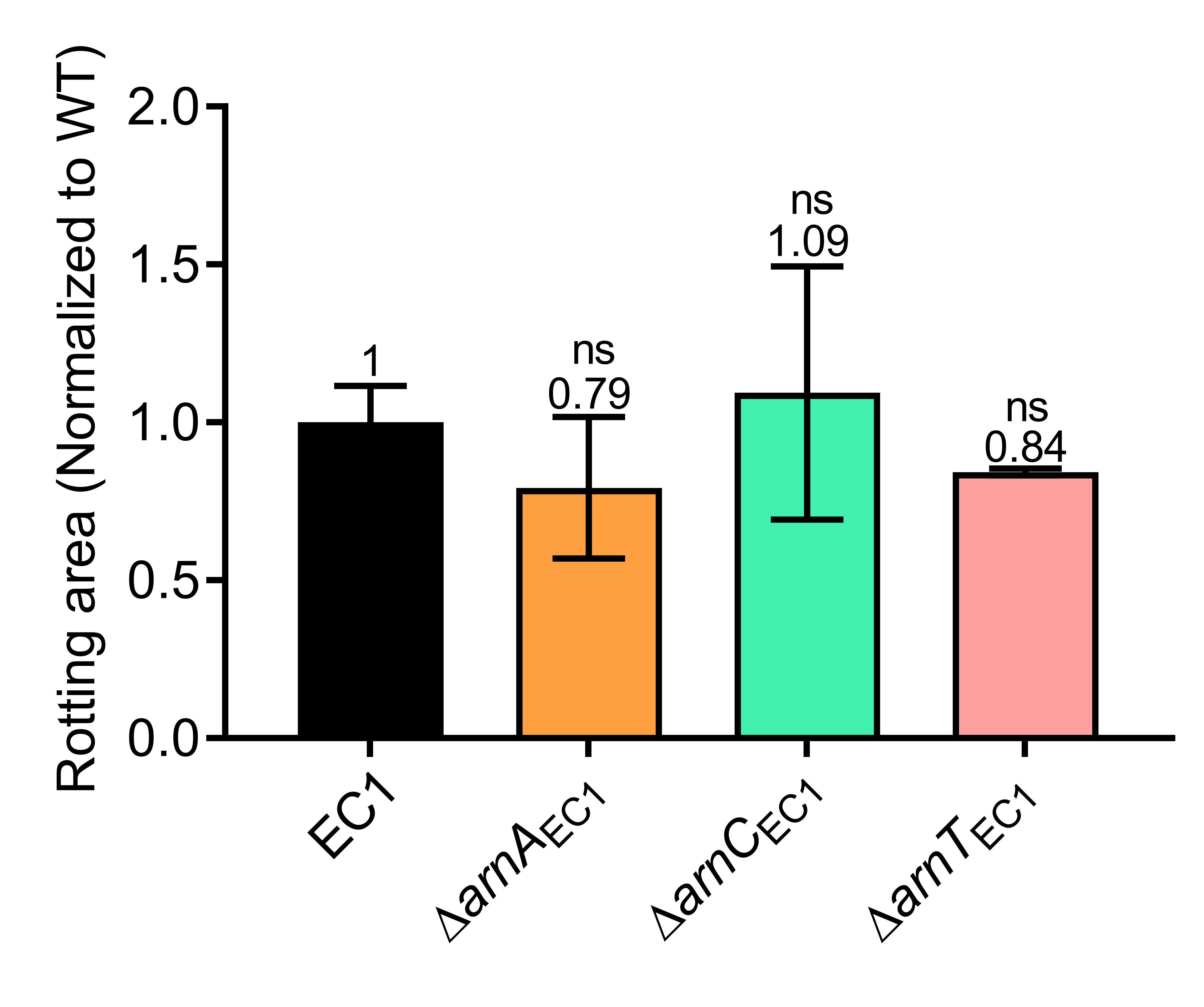


**Figure S1** Virulence of *arnACT*_EC1_ mutants against potato tubers. Two microlitres of bacterial suspension in double-distilled water (OD_600_ = 1.0) were inoculated in the sliced potato tubers. After incubation, the sliced potato tubers were photographed. The rotting areas caused by the wild-type (WT) strain EC1 and *arnACT*_EC1_ mutants were determined by ImageJ software (Schneider et al., 2012). The rotting area caused by the wild-type strain EC1 or *arnACT*_EC1_ mutants was normalized to that caused by the wild-type strain EC1, which was set as 1 for the convenience of comparison.

**Reference**

Schneider, C.A., Rasband, W.S. & Eliceiri, K.W. (2012) NIH Image to ImageJ: 25 years of image analysis. *Nat Methods,* 9, 671-675.
