# Supplementary material for: Characterization of the Arn lipopolysaccharide modification system essential for zeamine resistance unveils its new roles in Dickeya oryzae physiology and virulence
Source: Mol Plant Pathol. 2023 Sep 22;24(12):1480–94. doi: 10.1111/mpp.13386 (PMC10632790; doi:10.1111/mpp.13386)
Supplement: Supplementary file 2 — FIGURE S2 The arn EC1 genes are required for the bacterial nonmucoid morphotype. The morphotype of bacterial strains cultured on minimal medium (MM) agar plates supplemented with 5% (wt/vol) sucrose. WT, the wild‐type strain EC1; M, mutant; C, complementation strain; M‐CPS, arn EC1/cps1 double‐deletion mutant. [file MPP-24-1480-s004.doc]

**
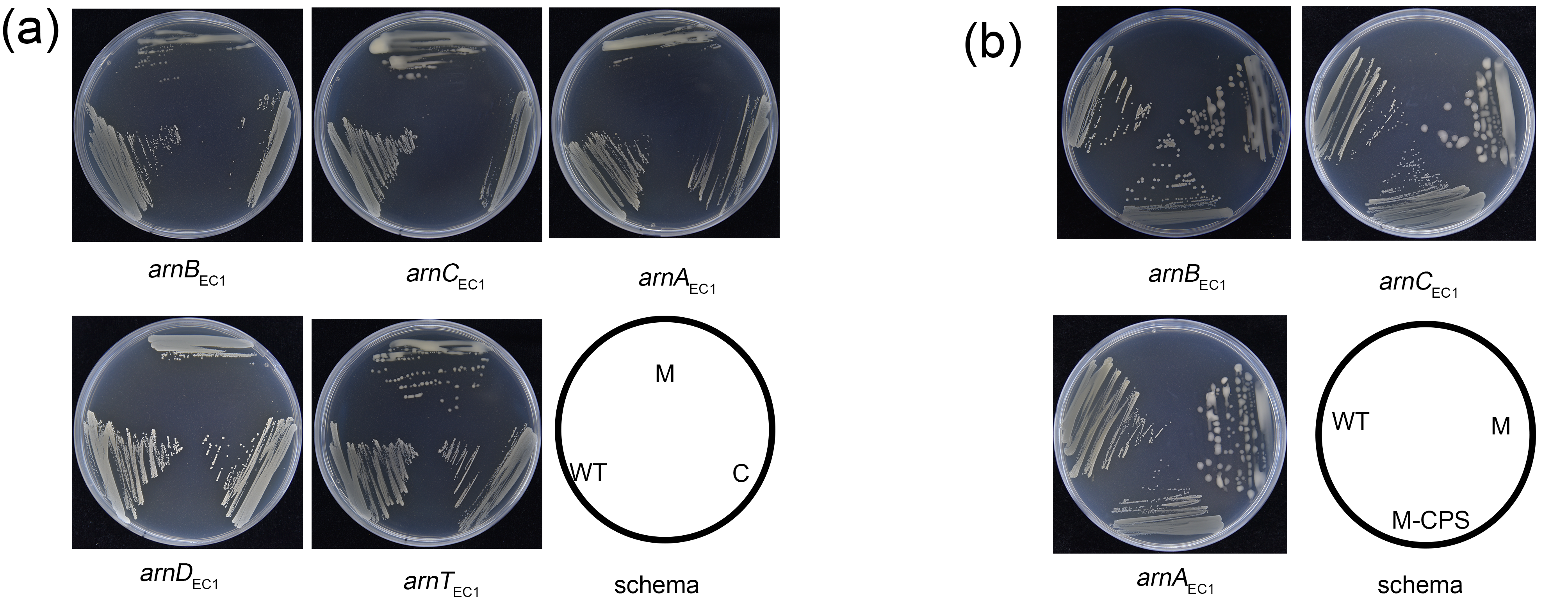
**

**Figure S2** The *arn*_EC1_ genes are required for bacterial non-mucoid morphotype. The morphotype of bacterial strains cultured on MM agar plate supplemented with 5% (wt/vol) sucrose. Symbol: WT, the wild-type strain EC1; M, mutant; C, complementation strain; M-CPS, *arn*_EC1_/*cps1* double-deletion mutant.
