# Supplementary material for: Characterization of the Arn lipopolysaccharide modification system essential for zeamine resistance unveils its new roles in Dickeya oryzae physiology and virulence
Source: Mol Plant Pathol. 2023 Sep 22;24(12):1480–94. doi: 10.1111/mpp.13386 (PMC10632790; doi:10.1111/mpp.13386)
Supplement: Supplementary file 3 — TABLE S1 Bacterial strains and plasmids used in this study. [file MPP-24-1480-s006.doc]

**TableS1 Bacterial strains and plasmids used in this study.**

| Strains or plasmids | Relevant characteristics^a^ | Source or reference |
| --- | --- | --- |
| *Dickeya oryzae* |  |  |
| EC1 | Wild-type strain of *D. oryzae* | (Hussain et al., 2008) |
| ∆*zmsA* | In-frame deletion of *zmsA* in EC1 | (Liang et al., 2019) |
| Zs1 | ∆*zmsA* derivative with transposon insertion at the 265^th^ base pair of the 1143-bp coding sequence of *arnB*_EC1_ | This study |
| Zs2 | ∆*zmsA* derivative with transposon insertion at the 285^th^ base pair of the 984-bp coding sequence of *arnC*_EC1_ | This study |
| Zs3 | ∆*zmsA* derivative with transposon insertion at the 544^th^ base pair of the 984-bp coding sequence of *arnC*_EC1_ | This study |
| Zs4 | ∆*zmsA* derivative with transposon insertion at the 561^th^ base pair of the 1992-bp coding sequence of *arnA*_EC1_ | This study |
| Zs5 | ∆*zmsA* derivative with transposon insertion at the 653^th^ base pair of the 1992-bp coding sequence of *arnA*_EC1_ | This study |
| Zs6 | ∆*zmsA* derivative with transposon insertion at the 1533^th^ base pair of the 1992-bp coding sequence of *arnA*_EC1_ | This study |
| Zs7 | ∆*zmsA* derivative with transposon insertion at the 376^th^ base pair of the 1653-bp coding sequence of *arnT*_EC1_ | This study |
| Zs8 | ∆*zmsA* derivative with transposon insertion at the 1356^th^ base pair of the 1653-bp coding sequence of *arnT*_EC1_ | This study |
| Zs9 | ∆*zmsA* derivative with transposon insertion at the 1557^th^ base pair of the 1653-bp coding sequence of *arnT*_EC1_ | This study |
| Zs7(pBB-*arnT*_EC1_) | Zs7 with pBB-*arnT*_EC1_ vector, Amp^r^ | This study |
| EC1(pDesAB_gfp_) | EC1 with the pDesAB_gfp_ vector, Kan^r^ | (Liang et al., 2019) |
| EC1(pArn_EC1gfp_) | EC1 with the pArn_EC1gfp_ vector, Kan^r^ | This study |
| ∆*arnB*_EC1_ | EC1 with the in-frame deletion of *arnB*_EC1_ | This study |
| ∆*arnC*_EC1_ | EC1 with the in-frame deletion of *arnC*_EC1_ | This study |
| ∆*arnA*_EC1_ | EC1 with the in-frame deletion of *arnA*_EC1_ | This study |
| ∆*arnD*_EC1_ | EC1 with the in-frame deletion of *arnD*_EC1_ | This study |
| ∆*arnT*_EC1_ | EC1 with the in-frame deletion of *arnT*_EC1_ | This study |
| ∆*arnB*_EC1_  (pBB-*arnB*_EC1_) | ∆*arnB*_EC1_ with the pBB-*arnB*_EC1_ vector, Amp^r^ | This study |
| ∆*arnC*_EC1_  (pBB-*arnC*_EC1_) | ∆*arnC*_EC1_ with the pBB-*arnC*_EC1_ vector, Amp^r^ | This study |
| ∆*arnA*_EC1_  (pBB-*arnA*_EC1_) | ∆*arnA*_EC1_ with the pBB-*arnA*_EC1_ vector, Amp^r^ | This study |
| ∆*arnD*_EC1_  (pBB-*arnD*_EC1_) | ∆*arnD*_EC1_ with the pBB-*arnD*_EC1_ vector, Amp^r^ | This study |
| ∆*arnT*_EC1_  (pBB-*arnT*_EC1_) | ∆*arnT*_EC1_ with the pBB-*arnT*_EC1_ vector, Amp^r^ | This study |
| ∆*zmsA*∆*arnB*_EC1_ | ∆*zmsA* with the in-frame deletion of *arnB*_EC1_ | This study |
| ∆*zmsA*∆*arnC*_EC1_ | ∆*zmsA* with the in-frame deletion of *arnC*_EC1_ | This study |
| ∆*zmsA*∆*arnA*_EC1_ | ∆*zmsA* with the in-frame deletion of *arnA*_EC1_ | This study |
| ∆*zmsA*∆*arnD*_EC1_ | ∆*zmsA* with the in-frame deletion of *arnD*_EC1_ | This study |
| ∆*zmsA*∆*arnT*_EC1_ | ∆*zmsA* with the in-frame deletion of *arnT*_EC1_ | This study |
| ∆*zmsA*∆*arnE*_EC1_ | ∆*zmsA* with the in-frame deletion of *arnE*_EC1_ | This study |
| ∆*zmsA*∆*arnF*_EC1_ | ∆*zmsA* with the in-frame deletion of *arnF*_EC1_ | This study |
| ∆*zmsA*∆*arnB*_EC1_∆*desB* | ∆*zmsA* with the *arnB*_EC1_/*desB* double deletion | This study |
| ∆*zmsA*∆*arnB*_EC1_  (pBB-*arnB*_EC1_) | ∆*zmsA*∆*arnB*_EC1_ with the pBB-*arnB*_EC1_ vector, Amp^r^ | This study |
| ∆*zmsA*∆*arnC*_EC1_  (pBB-*arnC*_EC1_) | ∆*zmsA*∆*arnC*_EC1_ with the pBB-*arnC*_EC1_ vector, Amp^r^ | This study |
| ∆*zmsA*∆*arnA*_EC1_  (pBB-*arnA*_EC1_) | ∆*zmsA*∆*arnA*_EC1_ with the pBB-*arnA*_EC1_ vector, Amp^r^ | This study |
| ∆*zmsA*∆*arnD*_EC1_  (pBB-*arnD*_EC1_) | ∆*zmsA*∆*arnD*_EC1_ with the pBB-*arnD*_EC1_ vector, Amp^r^ | This study |
| ∆*zmsA*∆*arnT*_EC1_  (pBB-*arnT*_EC1_) | ∆*zmsA*∆*arnT*_EC1_ with the pBB-*arnT*_EC1_ vector, Amp^r^ | This study |
| *Dickeya dadantii* |  |  |
| 3937 | The representative strain of *D. dadantii* | Laboratory collection |
| 3937(pAmob, pBB) | 3937 with pACYC184mob and pBBR1-MCS4 plasmids, Chl^r^, Amp^r^ | This study |
| 3937(pAmob-*arnBCADTEF*_EC1_, pBB-*ugd*_EC1_) | 3937 with pAmob-*arnBCADTEF*_EC1_ and pBB-*ugd*_EC1_ plasmids, Chl^r^, Amp^r^ | This study |
| *Escherichia coli* |  |  |
| DH5α | F-, φ80*lacZ*∆M15, ∆(*lacZYA*-*argF*)U169, *endA1*, *recA1*, *hsdR17* (r_k_^-^ ,m_k_^+^), *supE44*, λ^-^, *thi*-*1*, *gyrA96*, *relA1*, *phoA* | TransGen Biotech, China |
| CC118 | Host strain for the replication of pKNG101 and derivative plasmids | Laboratory collection |
| HB101 (pRK2013) | *thr leu thi recA hsdR hsdM pro*, Kan^r^ | Laboratory collection |
| Plasmids | | |
| pBT20 | Mariner based transposon plasmid, Gen^r^ | (Seet & Zhang, 2011) |
| pKNG101 | Suicide vector for gene in-frame deletion, Str^r^ | Laboratory collection |
| pKNG-*arnB*_EC1_ | pKNG101 harboring the flanking region of *arnB*_EC1_, Str^r^ | This study |
| pKNG-*arnC*_EC1_ | pKNG101 harboring the flanking region of *arnC*_EC1_, Str^r^ | This study |
| pKNG-*arnA*_EC1_ | pKNG101 harboring the flanking region of *arnA*_EC1_, Str^r^ | This study |
| pKNG-*arnD*_EC1_ | pKNG101 harboring the flanking region of *arnD*_EC1_, Str^r^ | This study |
| pKNG-*arnT*_EC1_ | pKNG101 harboring the flanking region of *arnT*_EC1_, Str^r^ | This study |
| pKNG-*arnE*_EC1_ | pKNG101 harboring the flanking region of *arnE*_EC1_, Str^r^ | This study |
| pKNG-*arnF*_EC1_ | pKNG101 harboring the flanking region of *arnF*_EC1_, Str^r^ | This study |
| pBB | Low copy vector pBBR1-MCS4 with *lac* promoter, Amp^r^ | Laboratory collection |
| pBB-*arnB*_EC1_ | pBBR1-MCS4 harboring the ORF of *arnB*_EC1_, Amp^r^ | This study |
| pBB-*arnC*_EC1_ | pBBR1-MCS4 harboring the ORF of *arnC*_EC1_, Amp^r^ | This study |
| pBB-*arnA*_EC1_ | pBBR1-MCS4 harboring the ORF of *arnA*_EC1_, Amp^r^ | This study |
| pBB-*arnD*_EC1_ | pBBR1-MCS4 harboring the ORF of *arnD*_EC1_, Amp^r^ | This study |
| pBB-*arnT*_EC1_ | pBBR1-MCS4 harboring the ORF of *arnT*_EC1_, Amp^r^ | This study |
| pBB-*ugd*_EC1_ | pBBR1-MCS4 harboring the ORF of *ugd*_EC1_, Amp^r^ | This study |
| pA | Low copy vector pACYC184, Tet^r^, Chl^r^ | Laboratory collection |
| pAmob-*arnBCADTEF*_EC1_ | pACYC184 with *mob* region harboring *arnBCADTEF*_EC1_ genes under the control of tetracycline resistance gene promoter *tetO*, Chl^r^ | This study |
| pPROBE-NT | Promoterless *gfp* transcriptional reporter plasmid, Kan^r^ | Laboratory collection |
| pDesAB_gfp_ | *Gfp* transcriptional fusion with the promoter region of *desAB* | (Liang et al., 2019) |
| pArn_EC1gfp_ | *Gfp* transcriptional fusion with the promoter region of *arn*_EC1_ operon | This study |

Abbreviations: Amp^r^, ampicillin resistance; Chl^r^, chloramphenicol resistance; Gen^r^: gentamycin resistance; Kan^r^, kanamycin resistance; Str^r^, streptomycin resistance; Tet^r^, tetracycline resistance.

**References**

Hussain, M.B., Zhang, H.B., Xu, J.L., Liu, Q., Jiang, Z. & Zhang, L.H. (2008) The acyl-homoserine lactone-type quorum-sensing system modulates cell motility and virulence of *Erwinia chrysanthemi* pv. zeae. *J Bacteriol,* 190, 1045-1053.

Liang, Z., Huang, L., He, F., Zhou, X., Shi, Z., Zhou, J. et al*.* (2019) A substrate-activated efflux pump, DesABC, confers zeamine resistance to *Dickeya zeae*. *mBio,* 10, e00713-19.

Seet, Q. & Zhang, L.H. (2011) Anti-activator QslA defines the quorum sensing threshold and response in *Pseudomonas aeruginosa*. *Mol Microbiol,* 80, 951-965.
