# Supplementary material for: Characterization of the Arn lipopolysaccharide modification system essential for zeamine resistance unveils its new roles in Dickeya oryzae physiology and virulence
Source: Mol Plant Pathol. 2023 Sep 22;24(12):1480–94. doi: 10.1111/mpp.13386 (PMC10632790; doi:10.1111/mpp.13386)
Supplement: Supplementary file 4 — TABLE S2 Primers used in this study. [file MPP-24-1480-s001.doc]

**Table S2 Primers used in this study.**

| Name | Sequence (5'–3') | Description | |
| --- | --- | --- | --- |
| Primers used in FPNI-PCR | | | |
| FP1 | GTAATACGACTCACTATAGGGCACGCGTGGTNTCGASTWTSGWGTT |  | |
| FP2 | GTAATACGACTCACTATAGGGCACGCGTGGTNGTCGASWGANAWGAA |
| FP3 | GTAATACGACTCACTATAGGGCACGCGTGGTWGTGNAGWANCANAGA |
| FP4 | GTAATACGACTCACTATAGGGCACGCGTGGTAGWGNAGWANCAWAGG |
| FP5 | GTAATACGACTCACTATAGGGCACGCGTGGTNGTAWAASGTNTSCAA |
| FP6 | GTAATACGACTCACTATAGGGCACGCGTGGTNGACGASWGANAWGAC |
| FP7 | GTAATACGACTCACTATAGGGCACGCGTGGTNGACGASWGANAWGAA |
| FP8 | GTAATACGACTCACTATAGGGCACGCGTGGTGTNCGASWCANAWGTT |
| FP9 | GTAATACGACTCACTATAGGGCACGCGTGGTNCAGCTWSCTNTSCTT |
| FSP1 | GTAATACGACTCACTATAGGGC |
| FSP2 | ACTATAGGGCACGCGTGGT |
| SP-1 | TGTTACGCAGCAGGGCAGTCGC |
| SP-2 | CCTACTCCCAACATCAGCCGGACTC |
| SP-3 | TACGGTGACGATCCCGCAGT |
| Primers for RT-PCR analysis | | | |
| RT-BC-F | CCTGCATCTGCCGAATACCG | Target the intergenic region of *arnBC*_EC1_ | |
| RT-BC-R | GAGGATCACCGCCACCACAT |
| RT-CA-F | ATGCGTCTGTTTTTCGGCGC | Target the intergenic region of *arnCA*_EC1_ | |
| RT-CA-R | GTATAGCCCGCAGCCACCAA |
| RT-AD-F | CTGGACTTCTTCCTTCAGACTGTTG | Target the intergenic region of *arnAD*_EC1_ | |
| RT-AD-R | GGCAGACCACGACCGATCAC |
| RT-DT-F | GTCTATACCATCCATGCCGAAGTG | Target the intergenic region of *arnDT*_EC1_ | |
| RT-DT-R | CAGTAACAACGCCAGCCATAGC |
| RT-TE-F | CCGTGAAGGTAATGTCTCATTGGTG | Target the intergenic region of *arnTE*_EC1_ | |
| RT-TE-R | GCCAGCCACAGCAGCATTG |
| RT-EF-F | CAATGCTGCTGTGGCTGGC | Target the intergenic region of *arnEF*_EC1_ | |
| RT-EF-R | GCACCGCCGTAGTCGCTATC |
| Primers for gene in-frame deletion | | | |
| b-1 | cgggatccCGCAAGGACCTAAAGGAGTG | *arnB*_EC1_ in-frame deletion | |
| b-2 | ATAAACAGATGCCAGGCGTGAGCACCGAGCAGAACGATCA |
| b-3 | TGATCGTTCTGCTCGGTGCTCACGCCTGGCATCTGTTTAT |
| b-4 | ggactagtCCGAATGTTGGCCGTAGTTA |
| c-1 | cgggatccCACGCCTGGCATCTGTTTAT | *arnC*_EC1_ in-frame deletion | |
| c-2 | CCGAAAAACAGACGCATCAGATTGTCGCTACTGCCGTCAT |
| c-3 | ATGACGGCAGTAGCGACAATCTGATGCGTCTGTTTTTCGG |
| c-4 | ggactagtCAGCAGGTGGCGATAGTAGA |
| a-1 | cgggatccACTGTTTTCCTTTATCGGCG | *arnA*_EC1_ in-frame deletion | |
| a-2 | ATGCTCGCTTCGTTTTCCGGCAGGTGGCGATAGTAGAAGG |
| a-3 | CCTTCTACTATCGCCACCTGCCGGAAAACGAAGCGAGCAT |
| a-4 | ggactagtGGTTTGCCAGGCGAAGTGAT |
| d-1 | cgggatccGGCTCGCCTATCAAACTGGT | *arnD*_EC1_ in-frame deletion | |
| d-2 | CTGAGCGGACAAAAGCGAATGCGCAGCATCTTCCACAAAA |
| d-3 | TTTTGTGGAAGATGCTGCGCATTCGCTTTTGTCCGCTCAG |
| d-4 | ggactagtACCAGCGTTATTGTCTCCCA |
| t-1 | cgggatccCGCTCAGTGAATTGCTACCG | *arnT*_EC1_ in-frame deletion | |
| t-2 | ATACCAACCAGCAACGCAATACCAGCGTTATTGTCTCCCA |
| t-3 | TGGGAGACAATAACGCTGGTATTGCGTTGCTGGTTGGTAT |
| t-4 | ggactagtCCCCAGCAACAACAGGCTTA |
| e-1 | cgggatccCGCTGGGTATTGCGTTGCTG | *arnE*_EC1_ in-frame deletion | |
| e-2 | CCATCCATAGCCTTTCACGCGGCGGCCTGTTTTTGGCTAA |
| e-3 | TTAGCCAAAAACAGGCCGCCGCGTGAAAGGCTATGGATG |
| e-4 | ggactagtCCGGGAAGTGAGAAACGGGA |
| f-1 | cgggatccCGGATCGTATGGTCTCTGCC | *arnF*_EC1_ in-frame deletion | |
| f-2 | CACACCAGCCATACACCGACCCATCCATAGCCTTTCACGC |
| f-3 | GCGTGAAAGGCTATGGATGGGTCGGTGTATGGCTGGTGTG |
| f-4 | ggactagtCCAGCACACTCCATTCCAGC |
| Primers for complementation and heterologous expression | | | |
| C-b-F | cccaagcttGAGAGAAAAGCGGGAGAGTG | | *arnB*_EC1_ complementation |
| C-b-R | cgggatccCGGGATCACCACCGAGACTT | |
| C-c-F | cccaagcttGGAACTCAGCGCGTATCTGC | | *arnC*_EC1_ complementation |
| C-c-R | cgggatccCGCGAATACCGGGATACCCA | |
| C-a-F | cccaagcttGGCTACTTGGGGAATATATCGGACG | | *arnA*_EC1_ complementation |
| C-a-R | cgggatccCGAGCGCAGCATCTTCCACAAAAAT | |
| C-d-F | cggaattcCGGAAAACGAAGCGAGCATT | | *arnD*_EC1_ complementation |
| C-d-R | ggactagtCCGGCTGATCTCCGCATAAC | |
| C-t-F | cccaagcttGGGAAACCAACACCGCCGAT | | *arnT*_EC1_ complementation |
| C-t-R | cgggatccCGCTCAACAACGCCCATCCA | |
| C-ugd-F | ggactagtGGATGAATGTGGACAGATGATG | | *ugd*_EC1_ heterologous expression |
| C-ugd-R | cgagctcGGCAACAGGGATAATCGCTT | |
| Primers for constructing the pAmob-*arnBCADTEF*_EC1_ | | | |
| arn_EC1_-F | ACCTGCTCGACTGCGGCAAGCTCTTCCTCACCTAACGCGGGGCGTGAGAAAGGTAGAAAATCAGCCATGTAAAAATTCACacacggtgcctgactgcgtt | |  |
| arn_EC1_-R | ATGGAGTGTGCTGGACTGGAATCAGCCCGCCATCGATTTCTACGACAGTCTGGGGGCTGCCCCACAAAACGAGTGGATCCggggagagcctgagcaaact | |
| Primers for amplifying the promoter region of *arn*_EC1_ operon for construction of pArn_EC1gfp_ | | | |
| P-Arn_EC1_-F | cccaagcttTTGATGTCTGTGTGATGGTTATCAC | |  |
| P-Arn_EC1_-R | cgggatccAATCAGCCATGTAAAAATTCACTC | |
| Primers for RT-qPCR analysis | | | |
| 16S-F | CCAGGTGTAGCGGTGAAATGC | | Target the 16S rRNA gene |
| 16S-R | CGGAAGCCACGGTTCAAGAC | |
| cps1-F | GACCGAAGTGCGTGATGAAATCATG | | Target the *cps1* gene |
| cps1-R | TCCTTGCCGTTATGCGTCAATACC | |
| L2-F | CAAAGTTACGACCTTCACCACCAC | | Target the L2 gene |
| L2-R | TTACCCTGCGTCTGCGTTCTG | |
| L3-F | CGTGGGTAGCGTCCTGAGTG | | Target the L3 gene |
| L3-R | CTAACCGTGTAACCAAGCCTGAAG | |
| L4-F | AGTCACGCCACCAGAACGC | | Target the L4 gene |
| L4-R | TCACCAGGTTGTTGTTGCTTATGC | |
| L17-F | ACGGAAGCCACACTTCAGAATACG | | Target the L17 gene |
| L17-R | CAAGACGACCCTGCCGAAAGC | |
| S10-F | CAACCAGACGCTTGTGAGTGC | | Target the S10 gene |
| S10-R | AAAGAATCCGTATCCGCCTGAAAG | |
| S11-F | CGTTATGAGGGATCGGAGTCACATC | | Target the S11 gene |
| S11-R | CAGGGTAATGCGTTGGGTTGGG | |
