# Supplementary material for: Characterization of the Arn lipopolysaccharide modification system essential for zeamine resistance unveils its new roles in Dickeya oryzae physiology and virulence
Source: Mol Plant Pathol. 2023 Sep 22;24(12):1480–94. doi: 10.1111/mpp.13386 (PMC10632790; doi:10.1111/mpp.13386)
Supplement: Supplementary file 5 — TABLE S3 Characteristics of the arn EC1 operon genes. [file MPP-24-1480-s003.doc]

**Table S3** Characteristics of the *arn*_EC1_ operon genes.

| Gene | Accession of the amino acid sequence in NCBI | Identity (similarity) at amino acid level compared to their homologues^a^ | | |
| --- | --- | --- | --- | --- |
|  |  | *Salmonella enterica* | *Pseudomonas aeruginosa* | *Dickeya dadantii* |
| *arnB*_EC1_ | WP_029456372 | 75% (86%) | 66% (81%) | 96% (98%) |
| *arnC*_EC1_ | WP_016941633 | 76% (88%) | 68% (82%) | 96% (98%) |
| *arnA*_EC1_ | WP_016941632 | 73% (84%) | 72% (82%) | 98% (98%) |
| *arnD*_EC1_ | WP_016941631 | 64% (72%) | 64% (74%) | 96% (98%) |
| *arnT*_EC1_ | WP_016941630 | 59% (72%) | 41% (61%) | 89% (94%) |
| *arnE*_EC1_ | WP_016941629 | 52% (69%) | 58% (73%) | 92% (96%) |
| *arnF*_EC1_ | WP_016941628 | 42% (57%) | 54% (65%) | 94% (96%) |

^a^The amino acid sequences of the *arn* operon genes in *S. enterica* subsp. *enterica* serovar Typhimurium LT2 (NCBI accession nos. NP_461239 to NP_461245), *P. aeruginosa* PAO1 (NCBI accession nos. NP_252242 to NP_252248), and *D. dadantii* 3937 (NCBI accession nos. WP_033112456 (*arnB*) and WP_013320119 to WP_013320114 (*arnCADTEF*)) were used for comparison.
