# Supplementary material for: Characterization of the Arn lipopolysaccharide modification system essential for zeamine resistance unveils its new roles in Dickeya oryzae physiology and virulence
Source: Mol Plant Pathol. 2023 Sep 22;24(12):1480–94. doi: 10.1111/mpp.13386 (PMC10632790; doi:10.1111/mpp.13386)
Supplement: Supplementary file 6 — TABLE S4 The up‐regulated genes in the arnB EC1 mutant compared to strain EC1. [file MPP-24-1480-s002.doc]

**Table S4 The up-regulated genes in *arnB*_EC1_ mutant compared to strain EC1.**

| Number | Gene_id | Gene description | log_2_ fold-change |
| --- | --- | --- | --- |
| B-1 | *W909_RS00060* | YitT family protein && PF02588:Uncharacterised 5xTM membrane BCR, YitT family COG1284 | 1.39 |
| B-2 | *W909_RS00200* | urea carboxylase && PF02626:Allophanate hydrolase subunit 2|PF02786:Carbamoyl-phosphate synthase L chain, ATP binding domain|PF02785:Biotin carboxylase C-terminal domain|PF00289:Carbamoyl-phosphate synthase L chain, N-terminal domain|PF02682:Allophanate hydrolase subunit 1|PF00364:Biotin-requiring enzyme | 1.59 |
| B-3 | *W909_RS00220* | ABC transporter permease subunit && PF00528:Binding-protein-dependent transport system inner membrane component | 2.59 |
| B-4 | *W909_RS00225* | ABC transporter substrate-binding protein && PF09084:NMT1/THI5 like | 3.33 |
| B-5 | *W909_RS00275* | xylose isomerase && - | 1.71 |
| B-6 | *W909_RS00280* | D-xylose ABC transporter substrate-binding protein && PF13407:Periplasmic binding protein domain | 2.15 |
| B-7 | *W909_RS00530* | LysR family transcriptional regulator && PF00126:Bacterial regulatory helix-turn-helix protein, lysR family|PF03466:LysR substrate binding domain | 1.20 |
| B-8 | *W909_RS00535* | dipeptidase && PF01244:Membrane dipeptidase (Peptidase family M19) | 1.07 |
| B-9 | *W909_RS00540* | ABC transporter substrate-binding protein && PF00496:Bacterial extracellular solute-binding proteins, family 5 Middle | 2.00 |
| B-10 | *W909_RS00545* | ABC transporter permease && PF00528:Binding-protein-dependent transport system inner membrane component | 2.28 |
| B-11 | *W909_RS00665* | DUF202 domain-containing protein && PF02656:Domain of unknown function (DUF202) | 1.16 |
| B-12 | *W909_RS00805* | murein hydrolase activator EnvC && PF01551:Peptidase family M23 | 1.00 |
| B-13 | *W909_RS00830* | lipid IV(A) palmitoyltransferase PagP && PF07017:Antimicrobial peptide resistance and lipid A acylation protein PagP | 3.50 |
| B-14 | *W909_RS00835* | DMT family transporter && PF00892:EamA-like transporter family | 1.79 |
| B-15 | *W909_RS00950* | transcription/translation regulatory transformer protein RfaH && PF02357:Transcription termination factor nusG | 1.42 |
| B-16 | *W909_RS01020* | Bifunctional biotin--[acetyl-CoA-carboxylase] ligase/biotin operon repressor BirA && PF03099:Biotin/lipoate A/B protein ligase family|PF08279:HTH domain|PF02237:Biotin protein ligase C terminal domain | 1.01 |
| B-17 | *W909_RS01115* | hypothetical protein && - | 1.62 |
| B-18 | *W909_RS01175* | YjaG family protein && PF04222:Protein of unknown function (DUF416) | 1.82 |
| B-19 | *W909_RS01240* | amino acid ABC transporter permease && PF00528:Binding-protein-dependent transport system inner membrane component | 2.29 |
| B-20 | *W909_RS01245* | amino acid ABC transporter substrate-binding protein && PF00497:Bacterial extracellular solute-binding proteins, family 3 | 2.11 |
| B-21 | *W909_RS01335* | protein-methionine-sulfoxide reductase heme-binding subunit MsrQ && PF01794:Ferric reductase like transmembrane component | 1.80 |
| B-22 | *W909_RS01340* | protein-methionine-sulfoxide reductase catalytic subunit MsrP && PF00174:Oxidoreductase molybdopterin binding domain | 2.04 |
| B-23 | *W909_RS01405* | p-hydroxybenzoic acid efflux pump subunit AaeB && PF04632:Fusaric acid resistance protein family | 1.10 |
| B-24 | *W909_RS01505* | phospholipid-binding protein MlaC && PF05494:MlaC protein | 1.66 |
| B-25 | *W909_RS01510* | lipid asymmetry maintenance protein MlaB && PF13466:STAS domain | 1.53 |
| B-26 | *W909_RS01530* | serine endoprotease DegQ && PF13365:Trypsin-like peptidase domain|PF00595:PDZ domain (Also known as DHR or GLGF)|PF13180:PDZ domain | 1.46 |
| B-27 | *W909_RS01735* | tol-pal system-associated acyl-CoA thioesterase && PF01618:MotA/TolQ/ExbB proton channel family | 1.55 |
| B-28 | *W909_RS01765* | aminoimidazole riboside kinase && PF00294:pfkB family carbohydrate kinase | 1.05 |
| B-29 | *W909_RS01915* | aspartate carbamoyltransferase && PF00185:Aspartate/ornithine carbamoyltransferase, Asp/Orn binding domain|PF02729:Aspartate/ornithine carbamoyltransferase, carbamoyl-P binding domain | 1.19 |
| B-30 | *W909_RS02045* | DUF945 domain-containing protein && PF06067:Domain of unknown function (DUF932) | 3.81 |
| B-31 | *W909_RS02195* | HNH endonuclease && PF13391:HNH endonuclease | 1.31 |
| B-32 | *W909_RS02295* | ABC transporter substrate-binding protein && PF00496:Bacterial extracellular solute-binding proteins, family 5 Middle | 1.77 |
| B-33 | *W909_RS02410* | ABC transporter ATP-binding protein && PF07673:Protein of unknown function (DUF1602)|PF08352:Oligopeptide/dipeptide transporter, C-terminal region|PF00005:ABC transporter | 1.36 |
| B-34 | *W909_RS02480* | 5-formyltetrahydrofolate cyclo-ligase && PF01812:5-formyltetrahydrofolate cyclo-ligase family | 1.14 |
| B-35 | *W909_RS02500* | YtjB family periplasmic protein && PF10144:Bacterial virulence factor haemolysin | 1.44 |
| B-36 | *W909_RS02520* | molecular chaperone OsmY && PF04972:BON domain | 4.40 |
| B-37 | *W909_RS02525* | stress response protein && - | 4.23 |
| B-38 | *W909_RS02530* | metal-dependent hydrolase && PF01026:TatD related DNase | 1.41 |
| B-39 | *W909_RS02565* | ABC transporter ATP-binding protein && PF00005:ABC transporter | 3.02 |
| B-40 | *W909_RS02760* | DUF883 domain-containing protein && PF05957:Bacterial protein of unknown function (DUF883) | 1.17 |
| B-41 | *W909_RS02765* | EnvZ/OmpR regulon moderator MzrA && PF13721:SecD export protein N-terminal TM region | 1.86 |
| B-42 | *W909_RS02770* | DedA family protein && PF09335:SNARE associated Golgi protein | 1.22 |
| B-43 | *W909_RS02845* | hexose-6-phosphate:phosphate antiporter && PF07690:Major Facilitator Superfamily | 1.67 |
| B-44 | *W909_RS02850* | ATP-independent periplasmic protein-refolding chaperone && PF07813:LTXXQ motif family protein | 3.18 |
| B-45 | *W909_RS02875* | GNAT family N-acetyltransferase && PF13302:Acetyltransferase (GNAT) domain | 1.25 |
| B-46 | *W909_RS03160* | DUF1435 domain-containing protein && PF07256:Protein of unknown function (DUF1435) | 1.34 |
| B-47 | *W909_RS03200* | hemolysin III family protein && PF03006:Haemolysin-III related | 2.13 |
| B-48 | *W909_RS03450* | polysaccharide biosynthesis protein && PF02719:Polysaccharide biosynthesis protein | 1.04 |
| B-49 | *W909_RS03495* | kdo(2)-lipid IV(A) palmitoleoyltransferase && PF03279:Bacterial lipid A biosynthesis acyltransferase | 1.12 |
| B-50 | *W909_RS03570* | L%2CD-transpeptidase family protein && PF03734:L,D-transpeptidase catalytic domain | 1.14 |
| B-51 | *W909_RS03735* | Nramp family divalent metal transporter && PF01566:Natural resistance-associated macrophage protein | 1.41 |
| B-52 | *W909_RS03900* | sulfate transporter CysZ && PF07264:Etoposide-induced protein 2.4 (EI24) | 1.37 |
| B-53 | *W909_RS03950* | DNA oxidative demethylase AlkB && PF13532:2OG-Fe(II) oxygenase superfamily | 1.11 |
| B-54 | *W909_RS04125* | conjugal transfer protein TraF && PF13729:F plasmid transfer operon, TraF, protein | 3.90 |
| B-55 | *W909_RS04130* | helix-turn-helix transcriptional regulator && PF01638:HxlR-like helix-turn-helix | 1.09 |
| B-56 | *W909_RS04160* | hypothetical protein && PF04914:DltD C-terminal region | 1.57 |
| B-57 | *W909_RS04265* | catalase KatB && PF06628:Catalase-related immune-responsive|PF00199:Catalase | 1.48 |
| B-58 | *W909_RS04410* | 1-deoxy-D-xylulose-5-phosphate reductoisomerase && PF08436:1-deoxy-D-xylulose 5-phosphate reductoisomerase C-terminal|PF13288:DXP reductoisomerase C-terminal domain|PF02670:1-deoxy-D-xylulose 5-phosphate reductoisomerase | 1.30 |
| B-59 | *W909_RS04500* | copper resistance protein NlpE N-terminal domain-containing protein && PF04170:NlpE N-terminal domain | 1.37 |
| B-60 | *W909_RS04570* | YgdI/YgdR family lipoprotein && PF06004:Bacterial protein of unknown function (DUF903) | 2.27 |
| B-61 | *W909_RS04600* | thymidylate synthase && PF00303:Thymidylate synthase | 1.24 |
| B-62 | *W909_RS04685* | YgdI/YgdR family lipoprotein && PF06004:Bacterial protein of unknown function (DUF903) | 4.75 |
| B-63 | *W909_RS04845* | hydroxyacylglutathione hydrolase && PF00753:Metallo-beta-lactamase superfamily|PF16123:Hydroxyacylglutathione hydrolase C-terminus | 1.73 |
| B-64 | *W909_RS04880* | YacC family pilotin-like protein && PF09691:Type II secretion system pilotin lipoprotein (PulS_OutS) | 1.12 |
| B-65 | *W909_RS04895* | hypothetical protein && - | 1.51 |
| B-66 | *W909_RS04975* | bifunctional glycosyl transferase/transpeptidase && PF14814:Bifunctional transglycosylase second domain|PF14812:Transmembrane domain of transglycosylase PBP1 at N-terminal|PF00905:Penicillin binding protein transpeptidase domain|PF00912:Transglycosylase | 1.01 |
| B-67 | *W909_RS05100* | P-II family nitrogen regulator && PF00543:Nitrogen regulatory protein P-II | 2.33 |
| B-68 | *W909_RS05105* | ammonium transporter AmtB && PF00909:Ammonium Transporter Family | 1.49 |
| B-69 | *W909_RS05115* | YbaY family lipoprotein && PF09619:Type III secretion system lipoprotein chaperone (YscW) | 4.06 |
| B-70 | *W909_RS05120* | MGMT family protein && PF01035:6-O-methylguanine DNA methyltransferase, DNA binding domain | 1.24 |
| B-71 | *W909_RS05125* | hemolysin expression modulator Hha && PF05321:Haemolysin expression modulating protein | 1.70 |
| B-72 | *W909_RS05130* | Hha toxicity modulator TomB && PF10757:Biofilm formation regulator YbaJ | 1.57 |
| B-73 | *W909_RS05285* | phosphotransferase RcsD && PF02518:Histidine kinase-, DNA gyrase B-, and HSP90-like ATPase|PF01627:Hpt domain|PF16359:RcsD-ABL domain | 1.32 |
| B-74 | *W909_RS05345* | ABC transporter ATP-binding protein && PF07673:Protein of unknown function (DUF1602)|PF00005:ABC transporter | 1.47 |
| B-75 | *W909_RS05355* | TonB-dependent receptor && PF07715:TonB-dependent Receptor Plug Domain|PF00593:TonB dependent receptor | 1.22 |
| B-76 | *W909_RS05360* | ABC transporter substrate-binding protein && PF01497:Periplasmic binding protein | 1.69 |
| B-77 | *W909_RS05870* | tol-pal system-associated acyl-CoA thioesterase && PF03061:Thioesterase superfamily | 1.14 |
| B-78 | *W909_RS05880* | colicin uptake protein TolR && PF02472:Biopolymer transport protein ExbD/TolR | 1.04 |
| B-79 | *W909_RS05920* | quinolinate synthase NadA && PF02445:Quinolinate synthetase A protein | 2.95 |
| B-80 | *W909_RS05925* | nicotinamide riboside transporter PnuC && PF04973:Nicotinamide mononucleotide transporter | 1.14 |
| B-81 | *W909_RS05950* | TolC family protein && PF02321:Outer membrane efflux protein | 2.90 |
| B-82 | *W909_RS05955* | copper-binding protein && PF11604:Copper binding periplasmic protein CusF | 2.05 |
| B-83 | *W909_RS05960* | hypothetical protein && - | 2.70 |
| B-84 | *W909_RS06040* | VirK family protein && PF06903:VirK protein | 1.16 |
| B-85 | *W909_RS06075* | polysaccharide export protein && PF02563:Polysaccharide biosynthesis/export protein | 5.69 |
| B-86 | *W909_RS06080* | protein-tyrosine-phosphatase && PF01451:Low molecular weight phosphotyrosine protein phosphatase | 6.30 |
| B-87 | *W909_RS06085* | tyrosine-protein kinase Wzc && PF13614:AAA domain|PF13807:G-rich domain on putative tyrosine kinase|PF02706:Chain length determinant protein | 4.92 |
| B-88 | *W909_RS06090* | glycosyltransferase family 4 protein && PF13579:Glycosyl transferase 4-like domain|PF13692:Glycosyl transferases group 1 | 4.05 |
| B-89 | *W909_RS06095* | glycosyltransferase && PF13579:Glycosyl transferase 4-like domain|PF13692:Glycosyl transferases group 1 | 4.04 |
| B-90 | *W909_RS06100* | glycosyltransferase family 4 protein && PF13439:Glycosyltransferase Family 4|PF00534:Glycosyl transferases group 1 | 6.75 |
| B-91 | *W909_RS06105* | glycosyltransferase family 2 protein && PF13641:Glycosyltransferase like family 2 | 5.98 |
| B-92 | *W909_RS06110* | undecaprenyl-phosphate glucose phosphotransferase && PF02397:Bacterial sugar transferase|PF13727:CoA-binding domain | 2.91 |
| B-93 | *W909_RS06115* | dTDP-glucose 4%2C6-dehydratase && PF16363:GDP-mannose 4,6 dehydratase | 2.41 |
| B-94 | *W909_RS06120* | glucose-1-phosphate thymidylyltransferase RfbA && PF00483:Nucleotidyl transferase | 2.64 |
| B-95 | *W909_RS06125* | dTDP-4-dehydrorhamnose 3%2C5-epimerase && PF00908:dTDP-4-dehydrorhamnose 3,5-epimerase | 2.57 |
| B-96 | *W909_RS06130* | dTDP-4-dehydrorhamnose reductase && PF04321:RmlD substrate binding domain | 2.56 |
| B-97 | *W909_RS06135* | glycosyltransferase family 2 protein && PF00535:Glycosyl transferase family 2 | 3.63 |
| B-98 | *W909_RS06140* | oligosaccharide flippase family protein && PF01943:Polysaccharide biosynthesis protein | 3.76 |
| B-99 | *W909_RS06145* | glycosyl transferase && - | 5.21 |
| B-100 | *W909_RS06150* | mannose-1-phosphate guanylyltransferase/mannose-6-phosphate isomerase && PF00483:Nucleotidyl transferase|PF01050:Mannose-6-phosphate isomerase | 6.63 |
| B-101 | *W909_RS06155* | phosphomannomutase CpsG && PF02880:Phosphoglucomutase/phosphomannomutase, alpha/beta/alpha domain III|PF02879:Phosphoglucomutase/phosphomannomutase, alpha/beta/alpha domain II|PF00408:Phosphoglucomutase/phosphomannomutase, C-terminal domain|PF02878:Phosphoglucomutase/phosphomannomutase, alpha/beta/alpha domain I | 3.57 |
| B-102 | *W909_RS06180* | hypothetical protein && - | 5.50 |
| B-103 | *W909_RS06185* | YjbF family lipoprotein && PF11102:Group 4 capsule polysaccharide lipoprotein gfcB, YjbF | 4.52 |
| B-104 | *W909_RS06190* | capsule biosynthesis GfcC family protein && PF06251:Capsule biosynthesis GfcC | 3.98 |
| B-105 | *W909_RS06195* | YjbH domain-containing protein && PF06082:Exopolysaccharide biosynthesis protein YbjH | 4.42 |
| B-106 | *W909_RS06200* | hypothetical protein && - | 3.23 |
| B-107 | *W909_RS06205* | hypothetical protein && - | 2.86 |
| B-108 | *W909_RS06225* | hypothetical protein && - | 1.32 |
| B-109 | *W909_RS06460* | FTR1 family protein && PF03239:Iron permease FTR1 family | 2.83 |
| B-110 | *W909_RS06555* | alpha/beta fold hydrolase && PF12695:Alpha/beta hydrolase family | 1.44 |
| B-111 | *W909_RS06560* | multidrug efflux RND transporter permease subunit && PF00873:AcrB/AcrD/AcrF family | 1.94 |
| B-112 | *W909_RS06565* | efflux RND transporter periplasmic adaptor subunit && PF13533:Biotin-lipoyl like|PF16576:Barrel-sandwich domain of CusB or HlyD membrane-fusion | 3.93 |
| B-113 | *W909_RS06660* | ABC transporter permease subunit && PF00528:Binding-protein-dependent transport system inner membrane component | 1.73 |
| B-114 | *W909_RS06715* | DUF2501 domain-containing protein && PF10696:Protein of unknown function (DUF2501) | 2.24 |
| B-115 | *W909_RS06785* | cyclic peptide export ABC transporter && PF00005:ABC transporter|PF00664:ABC transporter transmembrane region | 1.75 |
| B-116 | *W909_RS06795* | non-ribosomal peptide synthetase && PF00550:Phosphopantetheine attachment site|PF13193:AMP-binding enzyme C-terminal domain|PF00501:AMP-binding enzyme|PF00668:Condensation domain | 1.95 |
| B-117 | *W909_RS06880* | hypothetical protein && - | 1.62 |
| B-118 | *W909_RS06910* | LacI family DNA-binding transcriptional regulator && PF00356:Bacterial regulatory proteins, lacI family|PF13377:Periplasmic binding protein-like domain | 1.02 |
| B-119 | *W909_RS06915* | aspartate/glutamate racemase family protein && PF01177:Asp/Glu/Hydantoin racemase | 1.78 |
| B-120 | *W909_RS06975* | hydrogenase large subunit && PF00346:Respiratory-chain NADH dehydrogenase, 49 Kd subunit|PF00329:Respiratory-chain NADH dehydrogenase, 30 Kd subunit|PF00374:Nickel-dependent hydrogenase | 1.39 |
| B-121 | *W909_RS07000* | hydrogenase 4 subunit B && PF00361:Proton-conducting membrane transporter | 2.12 |
| B-122 | *W909_RS07055* | putrescine aminotransferase && PF00202:Aminotransferase class-III | 1.62 |
| B-123 | *W909_RS07170* | diaminobutyrate--2-oxoglutarate transaminase && PF00202:Aminotransferase class-III | 2.00 |
| B-124 | *W909_RS07180* | achromobactin biosynthetic protein AcsD && PF06276:Ferric iron reductase FhuF-like transporter|PF04183:IucA / IucC family | 1.10 |
| B-125 | *W909_RS07185* | type III PLP-dependent enzyme && PF02784:Pyridoxal-dependent decarboxylase, pyridoxal binding domain|PF00278:Pyridoxal-dependent decarboxylase, C-terminal sheet domain | 1.42 |
| B-126 | *W909_RS07190* | DHA2 family efflux MFS transporter permease subunit && PF07690:Major Facilitator Superfamily | 1.41 |
| B-127 | *W909_RS07195* | achromobactin biosynthetic protein AcsC && PF06276:Ferric iron reductase FhuF-like transporter|PF04183:IucA / IucC family | 1.09 |
| B-128 | *W909_RS07245* | MBL fold metallo-hydrolase && PF12706:Beta-lactamase superfamily domain | 1.10 |
| B-129 | *W909_RS07340* | peptidase && PF00112:Papain family cysteine protease | 3.20 |
| B-130 | *W909_RS07365* | DUF2076 domain-containing protein && PF09849:Uncharacterized protein conserved in bacteria (DUF2076) | 1.23 |
| B-131 | *W909_RS07390* | hypothetical protein && - | 4.35 |
| B-132 | *W909_RS07535* | LysR family transcriptional regulator && PF03466:LysR substrate binding domain|PF00126:Bacterial regulatory helix-turn-helix protein, lysR family | 1.38 |
| B-133 | *W909_RS07670* | uridine diphosphate-N-acetylglucosamine-binding protein YvcK && PF01933:Uncharacterised protein family UPF0052 | 1.37 |
| B-134 | *W909_RS07730* | acetyl-CoA carboxylase biotin carboxylase subunit && PF02785:Biotin carboxylase C-terminal domain|PF00289:Carbamoyl-phosphate synthase L chain, N-terminal domain|PF02786:Carbamoyl-phosphate synthase L chain, ATP binding domain | 1.98 |
| B-135 | *W909_RS07735* | allophanate hydrolase subunit 1 && PF02682:Allophanate hydrolase subunit 1 | 1.82 |
| B-136 | *W909_RS07740* | biotin-dependent carboxyltransferase && PF02626:Allophanate hydrolase subunit 2 | 1.89 |
| B-137 | *W909_RS07745* | allantoin permease && - | 1.31 |
| B-138 | *W909_RS07780* | hypothetical protein && PF15599:Immunity protein 63 | 1.07 |
| B-139 | *W909_RS07830* | IS21 family transposase && PF00665:Integrase core domain | 1.31 |
| B-140 | *W909_RS07840* | ATP-binding protein && PF13304:AAA domain, putative AbiEii toxin, Type IV TA system | 1.23 |
| B-141 | *W909_RS07920* | hypothetical protein && PF13989:YejG-like protein | 1.72 |
| B-142 | *W909_RS07985* | hypothetical protein && - | 1.45 |
| B-143 | *W909_RS08010* | sugar transporter && PF07690:Major Facilitator Superfamily | 1.86 |
| B-144 | *W909_RS08075* | outer membrane permeability protein SanA && PF02698:DUF218 domain | 1.43 |
| B-145 | *W909_RS08085* | PQQ-dependent sugar dehydrogenase && PF07995:Glucose / Sorbosone dehydrogenase | 1.72 |
| B-146 | *W909_RS08275* | outer membrane lipoprotein chaperone LolA && PF03548:Outer membrane lipoprotein carrier protein LolA | 1.37 |
| B-147 | *W909_RS08280* | replication-associated recombination protein A && PF00004:ATPase family associated with various cellular activities (AAA)|PF16193:AAA C-terminal domain|PF12002:MgsA AAA+ ATPase C terminal | 1.36 |
| B-148 | *W909_RS08295* | Tat proofreading chaperone DmsD && PF02613:Nitrate reductase delta subunit | 4.13 |
| B-149 | *W909_RS08645* | lipoprotein && PF07273:Protein of unknown function (DUF1439) | 2.22 |
| B-150 | *W909_RS08655* | multidrug efflux MFS transporter MdtH && PF07690:Major Facilitator Superfamily | 1.75 |
| B-151 | *W909_RS08780* | VOC family protein && PF06185:YecM protein | 1.88 |
| B-152 | *W909_RS08875* | hypothetical protein && - | 4.96 |
| B-153 | *W909_RS09140* | cardiolipin synthase && PF13396:Phospholipase_D-nuclease N-terminal|PF13091:PLD-like domain | 1.31 |
| B-154 | *W909_RS09155* | TonB system transport protein TonB && PF03544:Gram-negative bacterial TonB protein C-terminal|PF16031:TonB N-terminal region | 1.88 |
| B-155 | *W909_RS09310* | L-arabinose isomerase && PF11762:L-arabinose isomerase C-terminal domain|PF02610:L-arabinose isomerase | 2.32 |
| B-156 | *W909_RS09315* | ribulokinase && PF00370:FGGY family of carbohydrate kinases, N-terminal domain|PF02782:FGGY family of carbohydrate kinases, C-terminal domain | 2.26 |
| B-157 | *W909_RS09340* | oxidoreductase && PF01408:Oxidoreductase family, NAD-binding Rossmann fold|PF02894:Oxidoreductase family, C-terminal alpha/beta domain | 1.00 |
| B-158 | *W909_RS09435* | RNA polymerase sigma factor && PF04542:Sigma-70 region 2|PF08281:Sigma-70, region 4 | 1.15 |
| B-159 | *W909_RS09535* | glycerophosphoryl diester phosphodiesterase && PF03009:Glycerophosphoryl diester phosphodiesterase family | 1.43 |
| B-160 | *W909_RS09805* | Slp family lipoprotein && PF03843:Outer membrane lipoprotein Slp family | 1.21 |
| B-161 | *W909_RS09960* | osmotically-inducible lipoprotein OsmE && PF04355:SmpA / OmlA family | 2.07 |
| B-162 | *W909_RS10215* | hypothetical protein && - | 2.72 |
| B-163 | *W909_RS10260* | hypothetical protein && - | 1.15 |
| B-164 | *W909_RS10415* | type III secretion system stator protein SctL && PF06188:HrpE/YscL/FliH and V-type ATPase subunit E | 1.02 |
| B-165 | *W909_RS10680* | acyltransferase && PF01757:Acyltransferase family | 3.13 |
| B-166 | *W909_RS10755* | hypothetical protein && - | 2.67 |
| B-167 | *W909_RS10805* | DNA endonuclease SmrA && PF01713:Smr domain | 1.47 |
| B-168 | *W909_RS10905* | ABC transporter ATP-binding protein && PF00005:ABC transporter | 1.15 |
| B-169 | *W909_RS11000* | YdbH family protein && PF11739:Dicarboxylate transport | 1.76 |
| B-170 | *W909_RS11020* | methyl-accepting chemotaxis protein && - | 3.36 |
| B-171 | *W909_RS11075* | GNAT family N-acetyltransferase && PF13302:Acetyltransferase (GNAT) domain | 1.63 |
| B-172 | *W909_RS11130* | GTP-binding protein && PF07683:Cobalamin synthesis protein cobW C-terminal domain|PF02492:CobW/HypB/UreG, nucleotide-binding domain | 1.80 |
| B-173 | *W909_RS11170* | FdhF/YdeP family oxidoreductase && PF01568:Molydopterin dinucleotide binding domain|PF00384:Molybdopterin oxidoreductase | 1.95 |
| B-174 | *W909_RS11210* | osmotically-inducible lipoprotein OsmB && PF05433:Glycine zipper 2TM domain | 3.63 |
| B-175 | *W909_RS11270* | MliC family protein && PF09864:Membrane-bound lysozyme-inhibitor of c-type lysozyme | 3.28 |
| B-176 | *W909_RS11380* | hypothetical protein && - | 1.97 |
| B-177 | *W909_RS11535（11910）* | EAL domain-containing protein && PF00563:EAL domain | 3.64 |
| B-178 | *W909_RS11540* | lipoate--protein ligase A && PF03099:Biotin/lipoate A/B protein ligase family|PF10437:Bacterial lipoate protein ligase C-terminus | 1.39 |
| B-179 | *W909_RS11630* | glycine zipper 2TM domain-containing protein && PF05433:Glycine zipper 2TM domain | 4.51 |
| B-180 | *W909_RS11780* | cytochrome b && PF01292:Prokaryotic cytochrome b561 | 2.44 |
| B-181 | *W909_RS11785* | YceI family protein && PF04264:YceI-like domain | 2.35 |
| B-182 | *W909_RS12185* | protein DsrB && PF10781:Dextransucrase DSRB | 1.43 |
| B-183 | *W909_RS12190* | VOC family protein && PF12681:Glyoxalase-like domain | 1.07 |
| B-184 | *W909_RS12295* | replication endonuclease && PF05840:Bacteriophage replication gene A protein (GPA) | 1.96 |
| B-185 | *W909_RS12310* | phage portal protein && PF04860:Phage portal protein | 2.01 |
| B-186 | *W909_RS12315* | helix-turn-helix domain-containing protein && PF06056:Putative ATPase subunit of terminase (gpP-like)|PF03237:Terminase-like family | 2.39 |
| B-187 | *W909_RS12320* | GPO family capsid scaffolding protein && PF05929:Phage capsid scaffolding protein (GPO) serine peptidase | 2.57 |
| B-188 | *W909_RS12325* | phage major capsid protein%2C P2 family && PF05125:Phage major capsid protein, P2 family | 2.35 |
| B-189 | *W909_RS12330* | terminase && PF05944:Phage small terminase subunit | 2.47 |
| B-190 | *W909_RS12370* | phage virion morphogenesis protein && PF05069:Phage virion morphogenesis family | 2.48 |
| B-191 | *W909_RS12410* | phage tail tape measure protein && PF10145:Phage-related minor tail protein | 1.93 |
| B-192 | *W909_RS12430* | phage tail sheath family protein && PF04984:Phage tail sheath protein | 2.57 |
| B-193 | *W909_RS12550* | helix-turn-helix transcriptional regulator && PF01381:Helix-turn-helix | 1.35 |
| B-194 | *W909_RS12615* | type IV secretion system protein && PF07996:Type IV secretion system proteins | 3.44 |
| B-195 | *W909_RS12620* | type IV secretion system protein && PF04610:TrbL/VirB6 plasmid conjugal transfer protein | 2.68 |
| B-196 | *W909_RS12630* | P-type conjugative transfer protein VirB9 && PF03524:Conjugal transfer protein | 3.47 |
| B-197 | *W909_RS12635* | TrbI/VirB10 family protein && PF03743:Bacterial conjugation TrbI-like protein | 2.67 |
| B-198 | *W909_RS12715* | TatD family hydrolase && PF01026:TatD related DNase | 1.02 |
| B-199 | *W909_RS13135* | iron ABC transporter permease && PF00528:Binding-protein-dependent transport system inner membrane component | 1.51 |
| B-200 | *W909_RS13155* | transcriptional regulator TctD && PF00072:Response regulator receiver domain|PF00486:Transcriptional regulatory protein, C terminal | 1.03 |
| B-201 | *W909_RS13280* | malate dehydrogenase (quinone) && PF06039:Malate:quinone oxidoreductase (Mqo) | 1.05 |
| B-202 | *W909_RS13315* | enterobactin transporter EntS && PF05977:Transmembrane secretion effector | 1.14 |
| B-203 | *W909_RS13320* | Fe(3+)-siderophore ABC transporter permease && PF01032:FecCD transport family | 2.24 |
| B-204 | *W909_RS13335* | non-ribosomal peptide synthetase && PF00501:AMP-binding enzyme|PF00975:Thioesterase domain|PF13193:AMP-binding enzyme C-terminal domain|PF00668:Condensation domain|PF00550:Phosphopantetheine attachment site | 1.34 |
| B-205 | *W909_RS13355* | isochorismate synthase && PF00425:chorismate binding enzyme | 1.79 |
| B-206 | *W909_RS13365* | isochorismatase && PF00857:Isochorismatase family|PF00550:Phosphopantetheine attachment site | 1.96 |
| B-207 | *W909_RS13720* | ATP-dependent Clp protease proteolytic subunit && PF00574:Clp protease | 1.83 |
| B-208 | *W909_RS13880* | FtsH protease modulator YccA && PF01027:Inhibitor of apoptosis-promoting Bax1 | 1.66 |
| B-209 | *W909_RS13895* | heat shock protein HspQ && PF08755:Hemimethylated DNA-binding protein YccV like | 1.08 |
| B-210 | *W909_RS13995* | phosphate-starvation-inducible protein PsiE && PF06146:Phosphate-starvation-inducible E | 2.08 |
| B-211 | *W909_RS14235* | peptidase M4 family protein && PF16485:Protealysin propeptide|PF02868:Thermolysin metallopeptidase, alpha-helical domain|PF01447:Thermolysin metallopeptidase, catalytic domain | 1.46 |
| B-212 | *W909_RS14240* | hypothetical protein && - | 1.76 |
| B-213 | *W909_RS14555* | L-aspartate oxidase && PF00890:FAD binding domain|PF02910:Fumarate reductase flavoprotein C-term | 2.06 |
| B-214 | *W909_RS14685* | sulfate ABC transporter substrate-binding protein && PF13531:Bacterial extracellular solute-binding protein | 2.00 |
| B-215 | *W909_RS14695* | serine endoprotease DegP && PF13180:PDZ domain|PF13365:Trypsin-like peptidase domain|PF00595:PDZ domain (Also known as DHR or GLGF) | 2.21 |
| B-216 | *W909_RS14785* | DUF3251 domain-containing protein && PF11622:Protein of unknown function (DUF3251) | 2.80 |
| B-217 | *W909_RS14935* | SpoIIE family protein phosphatase && PF07228:Stage II sporulation protein E (SpoIIE) | 1.05 |
| B-218 | *W909_RS14960* | MFS transporter && PF05977:Transmembrane secretion effector | 1.32 |
| B-219 | *W909_RS15075* | hypothetical protein && - | 1.48 |
| B-220 | *W909_RS15085* | DNA polymerase IV && PF00817:impB/mucB/samB family|PF11799:impB/mucB/samB family C-terminal domain | 1.16 |
| B-221 | *W909_RS15115* | S-methyl-5-thioribose-1-phosphate isomerase && PF01008:Initiation factor 2 subunit family | 1.11 |
| B-222 | *W909_RS15220* | allophanate hydrolase && PF01425:Amidase | 2.11 |
| B-223 | *W909_RS15240* | glycine betaine/L-proline ABC transporter substrate-binding protein ProX && PF04069:Substrate binding domain of ABC-type glycine betaine transport system | 1.18 |
| B-224 | *W909_RS15410* | DUF3561 family protein && PF12084:Protein of unknown function (DUF3561) | 1.24 |
| B-225 | *W909_RS15415* | adenylyl-sulfate kinase && PF01583:Adenylylsulphate kinase | 1.08 |
| B-226 | *W909_RS15420* | sulfate adenylyltransferase subunit CysN && PF00009:Elongation factor Tu GTP binding domain|PF03144:Elongation factor Tu domain 2 | 1.37 |
| B-227 | *W909_RS15425* | sulfate adenylyltransferase subunit CysD && PF01507:Phosphoadenosine phosphosulfate reductase family | 1.50 |
| B-228 | *W909_RS15430* | uroporphyrinogen-III C-methyltransferase && PF14824:Sirohaem biosynthesis protein central|PF00590:Tetrapyrrole (Corrin/Porphyrin) Methylases|PF10414:Sirohaem synthase dimerisation region|PF13241:Putative NAD(P)-binding | 1.34 |
| B-229 | *W909_RS15595* | SH3 domain-containing protein && PF08239:Bacterial SH3 domain | 3.74 |
| B-230 | *W909_RS15605* | undecaprenyl-diphosphate phosphatase && PF02673:Bacitracin resistance protein BacA | 2.82 |
| B-231 | *W909_RS15635* | phenylalanine--tRNA ligase subunit alpha && PF01409:tRNA synthetases class II core domain (F) | 1.22 |
| B-232 | *W909_RS15675* | DUF1107 domain-containing protein && PF06526:Protein of unknown function (DUF1107) | 1.12 |
| B-233 | *W909_RS15695* | DUF2502 domain-containing protein && PF10697:Protein of unknown function (DUF2502) | 3.85 |
| B-234 | *W909_RS15925* | diaminopimelate decarboxylase && PF02784:Pyridoxal-dependent decarboxylase, pyridoxal binding domain|PF00278:Pyridoxal-dependent decarboxylase, C-terminal sheet domain | 4.27 |
| B-235 | *W909_RS16030* | amino acid permease && PF00324:Amino acid permease | 2.16 |
| B-236 | *W909_RS16040* | YitT family protein && PF02588:Uncharacterised 5xTM membrane BCR, YitT family COG1284 | 3.34 |
| B-237 | *W909_RS16125* | type VI secretion system tip protein VgrG && - | 2.62 |
| B-238 | *W909_RS16130* | PAAR domain-containing protein && PF05488:PAAR motif | 3.24 |
| B-239 | *W909_RS16210* | ABC transporter ATP-binding protein && PF07673:Protein of unknown function (DUF1602)|PF00005:ABC transporter | 2.54 |
| B-240 | *W909_RS16215* | metal ABC transporter permease && PF00950:ABC 3 transport family | 1.97 |
| B-241 | *W909_RS16315* | pyruvate dehydrogenase complex transcriptional repressor PdhR && PF07729:FCD domain|PF00392:Bacterial regulatory proteins, gntR family | 1.19 |
| B-242 | *W909_RS16520* | siderophore-interacting protein && PF04954:Siderophore-interacting protein|PF08021:Siderophore-interacting FAD-binding domain | 1.91 |
| B-243 | *W909_RS16565* | DedA family protein && PF09335:SNARE associated Golgi protein | 1.35 |
| B-244 | *W909_RS16580* | hypothetical protein && - | 1.28 |
| B-245 | *W909_RS16655* | type 3 dihydrofolate reductase && PF00186:Dihydrofolate reductase | 1.02 |
| B-246 | *W909_RS16700* | FKBP-type peptidyl-prolyl cis-trans isomerase && PF00254:FKBP-type peptidyl-prolyl cis-trans isomerase | 1.01 |
| B-247 | *W909_RS16900* | oxidative stress defense protein && PF04402:Protein of unknown function (DUF541) | 3.07 |
| B-248 | *W909_RS17055* | tRNA epoxyqueuosine(34) reductase QueG && PF13484:4Fe-4S double cluster binding domain|PF08331:Domain of unknown function (DUF1730) | 1.03 |
| B-249 | *W909_RS17230* | SDR family NAD(P)-dependent oxidoreductase && PF14765:Polyketide synthase dehydratase|PF00550:Phosphopantetheine attachment site|PF00109:Beta-ketoacyl synthase, N-terminal domain|PF16197:Ketoacyl-synthetase C-terminal extension|PF08659:KR domain|PF02801:Beta-ketoacyl synthase, C-terminal domain | 1.19 |
| B-250 | *W909_RS17235* | LLM class flavin-dependent oxidoreductase && PF00296:Luciferase-like monooxygenase | 1.87 |
| B-251 | *W909_RS17240* | KR domain-containing protein && PF00550:Phosphopantetheine attachment site|PF08659:KR domain|PF00109:Beta-ketoacyl synthase, N-terminal domain|PF02801:Beta-ketoacyl synthase, C-terminal domain|PF16197:Ketoacyl-synthetase C-terminal extension | 1.92 |
| B-252 | *W909_RS17245* | NAD(P)-binding domain-containing protein && PF00743:Flavin-binding monooxygenase-like | 2.21 |
| B-253 | *W909_RS17250* | FkbM family methyltransferase && PF00378:Enoyl-CoA hydratase/isomerase|PF00109:Beta-ketoacyl synthase, N-terminal domain|PF14765:Polyketide synthase dehydratase|PF05050:Methyltransferase FkbM domain|PF02801:Beta-ketoacyl synthase, C-terminal domain|PF00550:Phosphopantetheine attachment site|PF16197:Ketoacyl-synthetase C-terminal extension | 1.30 |
| B-254 | *W909_RS17255* | acyl carrier protein && - | 2.39 |
| B-255 | *W909_RS17260* | poly(3-hydroxyalkanoate) depolymerase && PF02801:Beta-ketoacyl synthase, C-terminal domain|PF00109:Beta-ketoacyl synthase, N-terminal domain | 2.00 |
| B-256 | *W909_RS17265* | hydroxymethylglutaryl-CoA synthase family protein && PF08540:Hydroxymethylglutaryl-coenzyme A synthase C terminal|PF01154:Hydroxymethylglutaryl-coenzyme A synthase N terminal | 1.53 |
| B-257 | *W909_RS17270* | enoyl-CoA hydratase/isomerase && PF00378:Enoyl-CoA hydratase/isomerase | 1.84 |
| B-258 | *W909_RS17275* | enoyl-CoA hydratase/isomerase family protein && PF00378:Enoyl-CoA hydratase/isomerase | 1.35 |
| B-259 | *W909_RS17280* | LysE family transporter && PF01810:LysE type translocator | 1.46 |
| B-260 | *W909_RS17420* | homoserine O-succinyltransferase && PF04204:Homoserine O-succinyltransferase | 1.06 |
| B-261 | *W909_RS17650* | bacterioferritin && PF00210:Ferritin-like domain | 1.78 |
| B-262 | *W909_RS17655* | bacterioferritin-associated ferredoxin && PF04324:BFD-like [2Fe-2S] binding domain | 2.14 |
| B-263 | *W909_RS17765* | oxalate decarboxylase family bicupin && PF00190:Cupin | 1.41 |
| B-264 | *W909_RS17810* | phosphoribulokinase && PF00485:Phosphoribulokinase / Uridine kinase family | 1.01 |
| B-265 | *W909_RS17970* | PilN domain-containing protein && PF05137:Fimbrial assembly protein (PilN) | 2.81 |
| B-266 | *W909_RS17985* | ADP compounds hydrolase NudE && PF00293:NUDIX domain | 1.95 |
| B-267 | *W909_RS18230* | homoserine/homoserine lactone efflux protein && PF01810:LysE type translocator | 1.02 |
| B-268 | *W909_RS18250* | thioesterase family protein && PF03061:Thioesterase superfamily | 1.35 |
| B-269 | *W909_RS18345* | amino acid ABC transporter substrate-binding protein && PF00497:Bacterial extracellular solute-binding proteins, family 3 | 1.71 |
| B-270 | *W909_RS18350* | GNAT family N-acetyltransferase && PF13508:Acetyltransferase (GNAT) domain | 2.83 |
| B-271 | *W909_RS18355* | adenylosuccinate lyase && PF00206:Lyase|PF10397:Adenylosuccinate lyase C-terminus | 2.42 |
| B-272 | *W909_RS18405* | ECA oligosaccharide polymerase && PF06899:WzyE protein | 1.14 |
| B-273 | *W909_RS18445* | UDP-N-acetylglucosamine--undecaprenyl-phosphate N-acetylglucosaminephosphotransferase && PF00953:Glycosyl transferase family 4 | 1.81 |
| B-274 | *W909_RS18550* | MFS transporter && PF07690:Major Facilitator Superfamily | 1.77 |
| B-275 | *W909_RS18630* | Si-specific NAD(P)(+) transhydrogenase && PF07992:Pyridine nucleotide-disulphide oxidoreductase|PF02852:Pyridine nucleotide-disulphide oxidoreductase, dimerisation domain | 1.12 |
| B-276 | *W909_RS18680* | cell division protein FtsN && PF05036:Sporulation related domain | 1.36 |
| B-277 | *W909_RS18825* | cell-envelope stress modulator CpxP && PF07813:LTXXQ motif family protein | 1.99 |
| B-278 | *W909_RS18860* | AEC family transporter && PF03547:Membrane transport protein | 1.02 |
| B-279 | *W909_RS18925* | MCP four helix bundle domain-containing protein && PF00015:Methyl-accepting chemotaxis protein (MCP) signalling domain|PF12729:Four helix bundle sensory module for signal transduction | 1.40 |
| B-280 | *W909_RS19210* | DUF1471 domain-containing protein && PF07338:Protein of unknown function (DUF1471) | 4.84 |
| B-281 | *W909_RS19420* | nitrogen regulation protein NR(II) && PF02518:Histidine kinase-, DNA gyrase B-, and HSP90-like ATPase|PF00512:His Kinase A (phospho-acceptor) domain|PF00989:PAS fold | 1.61 |
| B-282 | *W909_RS19425* | nitrogen regulation protein NR(I) && PF00158:Sigma-54 interaction domain|PF02954:Bacterial regulatory protein, Fis family|PF00072:Response regulator receiver domain | 1.61 |
| B-283 | *W909_RS19650* | ABC transporter substrate-binding protein && PF00497:Bacterial extracellular solute-binding proteins, family 3 | 1.84 |
| B-284 | *W909_RS19810* | non-ribosomal peptide synthetase && PF13193:AMP-binding enzyme C-terminal domain|PF13745:HxxPF-repeated domain|PF00501:AMP-binding enzyme|PF00975:Thioesterase domain|PF00550:Phosphopantetheine attachment site|PF00668:Condensation domain | 1.88 |
| B-285 | *W909_RS19975* | transposase && PF13358:DDE superfamily endonuclease|PF13565:Homeodomain-like domain | 1.62 |
| B-286 | *W909_RS20215* | antisense sRNA RprA && - | 3.14 |
| B-287 | *W909_RS20220* | ribosome modulation factor && PF04957:Ribosome modulation factor | 1.02 |
| B-288 | *W909_RS20275* | EexN family lipoprotein && - | 3.01 |
| B-289 | *W909_RS20580* | DUF4157 domain-containing protein && PF13699:Domain of unknown function (DUF4157) | 1.17 |
| B-290 | *W909_RS20615* | acid resistance repetitive basic protein Asr && - | 1.35 |
| B-291 | *W909_RS20750* | protein MgtS && - | 2.81 |
