# Supplementary material for: Characterization of the Arn lipopolysaccharide modification system essential for zeamine resistance unveils its new roles in Dickeya oryzae physiology and virulence
Source: Mol Plant Pathol. 2023 Sep 22;24(12):1480–94. doi: 10.1111/mpp.13386 (PMC10632790; doi:10.1111/mpp.13386)
Supplement: Supplementary file 7 — TABLE S5 The down‐regulated genes in the arnB EC1 mutant compared to strain EC1. [file MPP-24-1480-s007.doc]

**Table S5** The down-regulated genes in *arnB*_EC1_ mutant compared to strain EC1.

| Number | Gene_id | Gene description | log_2_ fold-change |
| --- | --- | --- | --- |
| B-1 | *W909_RS00075* | 4Fe-4S cluster-binding domain-containing protein && - | -1.12 |
| B-2 | *W909_RS00080* | ATP-grasp domain-containing protein && PF13535:ATP-grasp domain | -1.10 |
| B-3 | *W909_RS00085* | 2OG-Fe dioxygenase family protein && PF10014:2OG-Fe dioxygenase | -1.12 |
| B-4 | *W909_RS00105* | DUF3861 domain-containing protein && PF12977:Domain of Unknown Function with PDB structure (DUF3861) | -1.16 |
| B-5 | *W909_RS00110* | glucokinase && PF02685:Glucokinase | -1.52 |
| B-6 | *W909_RS00160* | aldose 1-epimerase family protein && PF14486:Domain of unknown function (DUF4432) | -1.01 |
| B-7 | *W909_RS00300* | DJ-1/PfpI family protein && PF01965:DJ-1/PfpI family | -1.74 |
| B-8 | *W909_RS00695* | DUF2202 domain-containing protein && - | -2.83 |
| B-9 | *W909_RS00705* | 50S ribosomal protein L28 && PF00830:Ribosomal L28 family | -1.22 |
| B-10 | *W909_RS00790* | glycine C-acetyltransferase && PF00155:Aminotransferase class I and II | -1.16 |
| B-11 | *W909_RS00845* | 5-methyltetrahydropteroyltriglutamate--homocysteine S-methyltransferase && PF08267:Cobalamin-independent synthase, N-terminal domain|PF01717:Cobalamin-independent synthase, Catalytic domain | -1.60 |
| B-12 | *W909_RS00855* | uridine phosphorylase && PF01048:Phosphorylase superfamily | -1.36 |
| B-13 | *W909_RS00860* | tyrosine-protein phosphatase && PF13350:Tyrosine phosphatase family|PF13348:Tyrosine phosphatase family C-terminal region | -1.09 |
| B-14 | *W909_RS00870* | PTS transporter subunit EIIC && PF02378:Phosphotransferase system, EIIC|PF00367:phosphotransferase system, EIIB | -2.44 |
| B-15 | *W909_RS00875* | 2-dehydro-3-deoxy-6-phosphogalactonate aldolase && PF13378:Enolase C-terminal domain-like|PF02746:Mandelate racemase / muconate lactonizing enzyme, N-terminal domain | -1.32 |
| B-16 | *W909_RS01040* | tRNA-Gly && - | -1.12 |
| B-17 | *W909_RS01045* | tRNA-Thr && - | -1.04 |
| B-18 | *W909_RS01050* | elongation factor Tu && PF00009:Elongation factor Tu GTP binding domain|PF03144:Elongation factor Tu domain 2|PF03143:Elongation factor Tu C-terminal domain | -1.01 |
| B-19 | *W909_RS01065* | 50S ribosomal protein L11 && PF03946:Ribosomal protein L11, N-terminal domain|PF00298:Ribosomal protein L11, RNA binding domain | -1.59 |
| B-20 | *W909_RS01070* | 50S ribosomal protein L1 && PF00687:Ribosomal protein L1p/L10e family | -1.40 |
| B-21 | *W909_RS01075* | 50S ribosomal protein L10 && PF00466:Ribosomal protein L10 | -1.15 |
| B-22 | *W909_RS01080* | 50S ribosomal protein L7/L12 && PF16320:Ribosomal protein L7/L12 dimerisation domain|PF00542:Ribosomal protein L7/L12 C-terminal domain | -1.01 |
| B-23 | *W909_RS01295* | DNA-binding transcriptional regulator Fis && PF02954:Bacterial regulatory protein, Fis family | -1.08 |
| B-24 | *W909_RS01345* | oxidoreductase && PF08240:Alcohol dehydrogenase GroES-like domain|PF00107:Zinc-binding dehydrogenase | -1.45 |
| B-25 | *W909_RS01430* | nucleoside diphosphate kinase regulator && PF01272:Transcription elongation factor, GreA/GreB, C-term|PF14760:Rnk N-terminus | -1.26 |
| B-26 | *W909_RS01545* | 50S ribosomal protein L13 && PF00572:Ribosomal protein L13 | -1.41 |
| B-27 | *W909_RS01550* | 30S ribosomal protein S9 && PF00380:Ribosomal protein S9/S16 | -1.67 |
| B-28 | *W909_RS01760* | carbohydrate porin && PF02264:LamB porin|PF11471:Maltoporin periplasmic N-terminal extension | -1.30 |
| B-29 | *W909_RS01840* | anaerobic ribonucleoside-triphosphate reductase && PF03477:ATP cone domain|PF13597:Anaerobic ribonucleoside-triphosphate reductase | -2.12 |
| B-30 | *W909_RS02005* | phospholipase D family protein && - | -1.40 |
| B-31 | *W909_RS02225* | transketolase && PF00456:Transketolase, thiamine diphosphate binding domain | -1.72 |
| B-32 | *W909_RS02230* | transketolase && PF02780:Transketolase, C-terminal domain|PF02779:Transketolase, pyrimidine binding domain | -2.38 |
| B-33 | *W909_RS02235* | D-threitol dehydrogenase && PF00106:short chain dehydrogenase | -1.49 |
| B-34 | *W909_RS02240* | D-ribose ABC transporter substrate-binding protein && PF13407:Periplasmic binding protein domain | -1.89 |
| B-35 | *W909_RS02360* | IclR family transcriptional regulator && PF09339:IclR helix-turn-helix domain|PF01614:Bacterial transcriptional regulator | -1.53 |
| B-36 | *W909_RS02365* | MCP four helix bundle domain-containing protein && PF00015:Methyl-accepting chemotaxis protein (MCP) signalling domain|PF12729:Four helix bundle sensory module for signal transduction | -2.17 |
| B-37 | *W909_RS02390* | HTH-type transcriptional activator RhaS && PF02311:AraC-like ligand binding domain|PF12833:Helix-turn-helix domain | -1.21 |
| B-38 | *W909_RS02395* | HTH-type transcriptional activator RhaR && PF12833:Helix-turn-helix domain|PF02311:AraC-like ligand binding domain | -1.54 |
| B-39 | *W909_RS02510* | hypothetical protein && - | -1.01 |
| B-40 | *W909_RS02580* | alkyl hydroperoxide reductase subunit C && PF00578:AhpC/TSA family|PF10417:C-terminal domain of 1-Cys peroxiredoxin | -1.43 |
| B-41 | *W909_RS02620* | L-talarate/galactarate dehydratase && PF13378:Enolase C-terminal domain-like|PF02746:Mandelate racemase / muconate lactonizing enzyme, N-terminal domain | -1.07 |
| B-42 | *W909_RS02675* | anaerobic C4-dicarboxylate transporter && PF03605:Anaerobic c4-dicarboxylate membrane transporter | -2.00 |
| B-43 | *W909_RS02680* | aspartate ammonia-lyase && PF10415:Fumarase C C-terminus|PF00206:Lyase | -1.18 |
| B-44 | *W909_RS02930* | 50S ribosomal protein L21 && PF00829:Ribosomal prokaryotic L21 protein | -1.02 |
| B-45 | *W909_RS02935* | 50S ribosomal protein L27 && PF01016:Ribosomal L27 protein | -1.14 |
| B-46 | *W909_RS02960* | ribosome assembly RNA-binding protein YhbY && PF01985:CRS1 / YhbY (CRM) domain | -1.07 |
| B-47 | *W909_RS02990* | tRNA-Leu && - | -1.09 |
| B-48 | *W909_RS03020* | tRNA pseudouridine(55) synthase TruB && PF09157:Pseudouridine synthase II TruB, C-terminal|PF01509:TruB family pseudouridylate synthase (N terminal domain)|PF16198:tRNA pseudouridylate synthase B C-terminal domain | -1.06 |
| B-49 | *W909_RS03025* | 30S ribosomal protein S15 && PF00312:Ribosomal protein S15 | -1.11 |
| B-50 | *W909_RS03055* | U32 family peptidase && PF01136:Peptidase family U32 | -2.72 |
| B-51 | *W909_RS03060* | U32 family peptidase && PF01136:Peptidase family U32 | -2.31 |
| B-52 | *W909_RS03070* | N-acetyltransferase && PF00583:Acetyltransferase (GNAT) family | -1.16 |
| B-53 | *W909_RS03400* | hypothetical protein && PF13440:Polysaccharide biosynthesis protein | -1.15 |
| B-54 | *W909_RS03405* | hypothetical protein && - | -1.10 |
| B-55 | *W909_RS03630* | CoA-acylating methylmalonate-semialdehyde dehydrogenase && PF00171:Aldehyde dehydrogenase family | -1.15 |
| B-56 | *W909_RS03640* | inositol 2-dehydrogenase && PF01408:Oxidoreductase family, NAD-binding Rossmann fold|PF02894:Oxidoreductase family, C-terminal alpha/beta domain | -1.74 |
| B-57 | *W909_RS03645* | substrate-binding domain-containing protein && PF13407:Periplasmic binding protein domain | -1.85 |
| B-58 | *W909_RS03650* | sugar ABC transporter ATP-binding protein && PF00005:ABC transporter|PF07673:Protein of unknown function (DUF1602) | -1.96 |
| B-59 | *W909_RS03660* | Gfo/Idh/MocA family oxidoreductase && PF01408:Oxidoreductase family, NAD-binding Rossmann fold|PF02894:Oxidoreductase family, C-terminal alpha/beta domain | -1.02 |
| B-60 | *W909_RS03785* | LysR family transcriptional regulator && PF03466:LysR substrate binding domain|PF00126:Bacterial regulatory helix-turn-helix protein, lysR family | -1.03 |
| B-61 | *W909_RS03820* | AraC family transcriptional regulator && PF07883:Cupin domain|PF12833:Helix-turn-helix domain | -1.03 |
| B-62 | *W909_RS03855* | cytosine permease && PF02133:Permease for cytosine/purines, uracil, thiamine, allantoin | -1.08 |
| B-63 | *W909_RS03930* | fumarate hydratase && PF05681:Fumarate hydratase (Fumerase)|PF05683:Fumarase C-terminus | -1.24 |
| B-64 | *W909_RS03985* | LysR family transcriptional regulator && PF03466:LysR substrate binding domain|PF00126:Bacterial regulatory helix-turn-helix protein, lysR family | -1.43 |
| B-65 | *W909_RS04210* | GTPase && PF02492:CobW/HypB/UreG, nucleotide-binding domain|PF07683:Cobalamin synthesis protein cobW C-terminal domain | -1.02 |
| B-66 | *W909_RS04390* | 30S ribosomal protein S2 && PF00318:Ribosomal protein S2 | -1.46 |
| B-67 | *W909_RS04395* | elongation factor Ts && PF00889:Elongation factor TS | -1.32 |
| B-68 | *W909_RS04745* | 30S ribosomal protein S16 && PF00886:Ribosomal protein S16 | -1.06 |
| B-69 | *W909_RS04870* | adenosylmethionine decarboxylase && PF02675:S-adenosylmethionine decarboxylase | -1.63 |
| B-70 | *W909_RS05000* | protoheme IX farnesyltransferase && PF01040:UbiA prenyltransferase family | -1.50 |
| B-71 | *W909_RS05005* | cytochrome o ubiquinol oxidase subunit IV && PF03626:Prokaryotic Cytochrome C oxidase subunit IV | -1.30 |
| B-72 | *W909_RS05010* | cytochrome o ubiquinol oxidase subunit III && PF00510:Cytochrome c oxidase subunit III | -1.39 |
| B-73 | *W909_RS05015* | cytochrome o ubiquinol oxidase subunit I && PF00115:Cytochrome C and Quinol oxidase polypeptide I | -1.65 |
| B-74 | *W909_RS05020* | cytochrome o ubiquinol oxidase subunit II && PF06481:COX Aromatic Rich Motif | -1.88 |
| B-75 | *W909_RS05040* | trigger factor && PF05697:Bacterial trigger factor protein (TF)|PF00254:FKBP-type peptidyl-prolyl cis-trans isomerase|PF05698:Bacterial trigger factor protein (TF) C-terminus | -1.40 |
| B-76 | *W909_RS05060* | DNA-binding protein HU-beta && PF00216:Bacterial DNA-binding protein | -1.08 |
| B-77 | *W909_RS05200* | adenylate kinase && PF00406:Adenylate kinase|PF05191:Adenylate kinase, active site lid | -1.50 |
| B-78 | *W909_RS05220* | methyl-accepting chemotaxis protein && PF00015:Methyl-accepting chemotaxis protein (MCP) signalling domain|PF08269:Cache domain | -1.44 |
| B-79 | *W909_RS05375* | galactose/methyl galactoside ABC transporter permease MglC && PF02653:Branched-chain amino acid transport system / permease component | -1.87 |
| B-80 | *W909_RS05380* | galactose/methyl galactoside ABC transporter ATP-binding protein MglA && PF00005:ABC transporter | -2.00 |
| B-81 | *W909_RS05385* | galactose/glucose ABC transporter substrate-binding protein MglB && PF13407:Periplasmic binding protein domain | -1.63 |
| B-82 | *W909_RS05395* | HTH-type transcriptional regulator GalS && PF00356:Bacterial regulatory proteins, lacI family|PF13377:Periplasmic binding protein-like domain | -1.28 |
| B-83 | *W909_RS05410* | uracil phosphoribosyltransferase && PF14681:Uracil phosphoribosyltransferase | -1.35 |
| B-84 | *W909_RS05490* | ArsC family reductase && PF03960:ArsC family | -1.19 |
| B-85 | *W909_RS05585* | amino acid ABC transporter ATP-binding protein && PF00005:ABC transporter|PF07673:Protein of unknown function (DUF1602) | -1.06 |
| B-86 | *W909_RS05590* | glutamate/aspartate ABC transporter permease GltK && PF00528:Binding-protein-dependent transport system inner membrane component | -1.00 |
| B-87 | *W909_RS05600* | glutamate/aspartate ABC transporter substrate-binding protein && PF00497:Bacterial extracellular solute-binding proteins, family 3 | -1.13 |
| B-88 | *W909_RS05670* | asparagine synthase B && PF13537:Glutamine amidotransferase domain|PF00733:Asparagine synthase | -1.33 |
| B-89 | *W909_RS05810* | citrate synthase && PF00285:Citrate synthase | -1.28 |
| B-90 | *W909_RS05830* | succinate dehydrogenase iron-sulfur subunit && PF13085:2Fe-2S iron-sulfur cluster binding domain|PF13534:4Fe-4S dicluster domain | -1.41 |
| B-91 | *W909_RS05835* | 2-oxoglutarate dehydrogenase E1 component && PF16870:2-oxoglutarate dehydrogenase C-terminal|PF02779:Transketolase, pyrimidine binding domain|PF16078:2-oxoglutarate dehydrogenase N-terminus|PF00676:Dehydrogenase E1 component | -1.05 |
| B-92 | *W909_RS05855* | cytochrome ubiquinol oxidase subunit I && PF01654:Cytochrome bd terminal oxidase subunit I | -1.44 |
| B-93 | *W909_RS05895* | peptidoglycan-associated lipoprotein Pal && PF00691:OmpA family | -1.24 |
| B-94 | *W909_RS05940* | efflux RND transporter permease subunit && PF00873:AcrB/AcrD/AcrF family | -1.58 |
| B-95 | *W909_RS05975* | galactose-1-phosphate uridylyltransferase && PF02744:Galactose-1-phosphate uridyl transferase, C-terminal domain|PF01087:Galactose-1-phosphate uridyl transferase, N-terminal domain | -1.25 |
| B-96 | *W909_RS05980* | UDP-glucose 4-epimerase GalE && PF16363:GDP-mannose 4,6 dehydratase | -1.21 |
| B-97 | *W909_RS06270* | ankyrin repeat domain-containing protein && - | -1.11 |
| B-98 | *W909_RS06325* | hypothetical protein && - | -1.32 |
| B-99 | *W909_RS06355* | type VI secretion system contractile sheath small subunit && PF05591:Type VI secretion system, VipA, VC_A0107 or Hcp2 | -1.21 |
| B-100 | *W909_RS06360* | type VI secretion system contractile sheath large subunit && PF05943:Type VI secretion protein, EvpB/VC_A0108, tail sheath | -1.61 |
| B-101 | *W909_RS06365* | type VI secretion system baseplate subunit TssE && PF04965:Gene 25-like lysozyme | -1.04 |
| B-102 | *W909_RS06440* | D-amino-acid transaminase && PF01063:Amino-transferase class IV | -1.10 |
| B-103 | *W909_RS06445* | dipeptide epimerase && PF02746:Mandelate racemase / muconate lactonizing enzyme, N-terminal domain|PF13378:Enolase C-terminal domain-like | -1.11 |
| B-104 | *W909_RS06675* | methyltransferase domain-containing protein && PF08241:Methyltransferase domain | -3.91 |
| B-105 | *W909_RS06680* | nickel ABC transporter%2C nickel/metallophore periplasmic binding protein && PF00496:Bacterial extracellular solute-binding proteins, family 5 Middle | -4.18 |
| B-106 | *W909_RS06685* | ABC transporter permease subunit && PF00528:Binding-protein-dependent transport system inner membrane component | -2.47 |
| B-107 | *W909_RS06855* | chlorinating enzyme && PF05721:Phytanoyl-CoA dioxygenase (PhyH) | -1.65 |
| B-108 | *W909_RS06925* | dicarboxylate/amino acid:cation symporter && PF00375:Sodium:dicarboxylate symporter family | -2.45 |
| B-109 | *W909_RS06935* | formate dehydrogenase subunit alpha && PF01568:Molydopterin dinucleotide binding domain|PF04879:Molybdopterin oxidoreductase Fe4S4 domain|PF00384:Molybdopterin oxidoreductase | -1.67 |
| B-110 | *W909_RS07025* | glutamate/aspartate:proton symporter GltP && PF00375:Sodium:dicarboxylate symporter family | -1.06 |
| B-111 | *W909_RS07035* | anaerobic C4-dicarboxylate transporter && PF03605:Anaerobic c4-dicarboxylate membrane transporter | -1.91 |
| B-112 | *W909_RS07070* | hypothetical protein && - | -1.47 |
| B-113 | *W909_RS07305* | formate dehydrogenase-N subunit alpha && PF04879:Molybdopterin oxidoreductase Fe4S4 domain|PF01568:Molydopterin dinucleotide binding domain|PF00384:Molybdopterin oxidoreductase | -2.48 |
| B-114 | *W909_RS07310* | formate dehydrogenase subunit beta && PF13247:4Fe-4S dicluster domain|PF09163:Formate dehydrogenase N, transmembrane | -2.28 |
| B-115 | *W909_RS07315* | formate dehydrogenase-N subunit gamma && PF00033:Cytochrome b/b6/petB | -2.10 |
| B-116 | *W909_RS07325* | alpha-galactosidase && PF02065:Melibiase|PF16875:Glycosyl hydrolase family 36 N-terminal domain|PF16874:Glycosyl hydrolase family 36 C-terminal domain | -1.42 |
| B-117 | *W909_RS07405* | ABC transporter substrate-binding protein && PF00496:Bacterial extracellular solute-binding proteins, family 5 Middle | -1.35 |
| B-118 | *W909_RS07460* | chemotaxis protein CheV && PF01584:CheW-like domain|PF00072:Response regulator receiver domain | -1.23 |
| B-119 | *W909_RS07515* | hypothetical protein && - | -1.38 |
| B-120 | *W909_RS07555* | DUF3168 domain-containing protein && PF11367:Protein of unknown function (DUF3168) | -1.51 |
| B-121 | *W909_RS07560* | discoidin domain-containing protein && PF00754:F5/8 type C domain | -1.10 |
| B-122 | *W909_RS07615* | glycoside hydrolase family 3 protein && PF00933:Glycosyl hydrolase family 3 N terminal domain | -2.12 |
| B-123 | *W909_RS07685* | GTP 3'%2C8-cyclase MoaA && PF04055:Radical SAM superfamily|PF06463:Molybdenum Cofactor Synthesis C|PF13353:4Fe-4S single cluster domain | -1.21 |
| B-124 | *W909_RS07690* | molybdenum cofactor biosynthesis protein B && PF00994:Probable molybdopterin binding domain | -1.02 |
| B-125 | *W909_RS07695* | cyclic pyranopterin monophosphate synthase MoaC && PF01967:MoaC family | -1.56 |
| B-126 | *W909_RS07700* | molybdopterin synthase sulfur carrier subunit && PF02597:ThiS family | -1.26 |
| B-127 | *W909_RS07705* | molybdopterin synthase catalytic subunit MoaE && PF02391:MoaE protein | -1.19 |
| B-128 | *W909_RS07765* | protease inhibitor I42 family protein && PF09394:Chagasin family peptidase inhibitor I42 | -1.39 |
| B-129 | *W909_RS07890* | 50S ribosomal protein L25 && PF01386:Ribosomal L25p family | -1.58 |
| B-130 | *W909_RS07995* | YeiH family putative sulfate export transporter && PF03601:Conserved hypothetical protein 698 | -2.22 |
| B-131 | *W909_RS08140* | spermidine/putrescine ABC transporter substrate-binding protein PotF && PF13416:Bacterial extracellular solute-binding protein | -1.13 |
| B-132 | *W909_RS08145* | putrescine ABC transporter ATP-binding subunit PotG && PF00005:ABC transporter|PF08402:TOBE domain | -1.47 |
| B-133 | *W909_RS08285* | serine--tRNA ligase && PF02403:Seryl-tRNA synthetase N-terminal domain|PF00587:tRNA synthetase class II core domain (G, H, P, S and T) | -1.08 |
| B-134 | *W909_RS08335* | formate C-acetyltransferase && PF02901:Pyruvate formate lyase-like|PF01228:Glycine radical | -1.28 |
| B-135 | *W909_RS08340* | formate transporter FocA && PF01226:Formate/nitrite transporter | -2.16 |
| B-136 | *W909_RS08365* | 30S ribosomal protein S1 && PF00575:S1 RNA binding domain | -1.03 |
| B-137 | *W909_RS08430* | HAMP domain-containing protein && PF00015:Methyl-accepting chemotaxis protein (MCP) signalling domain|PF00672:HAMP domain | -1.39 |
| B-138 | *W909_RS08445* | 2-hydroxycarboxylate transporter family protein && PF03390:2-hydroxycarboxylate transporter family | -2.28 |
| B-139 | *W909_RS08495* | hypothetical protein && - | -1.35 |
| B-140 | *W909_RS08500* | APC family permease && PF13520:Amino acid permease | -1.14 |
| B-141 | *W909_RS08510* | DUF496 family protein && PF04363:Protein of unknown function (DUF496) | -1.04 |
| B-142 | *W909_RS08605* | ribonuclease T2 && PF00445:Ribonuclease T2 family | -1.08 |
| B-143 | *W909_RS08620* | DUF4917 family protein && PF16263:Domain of unknown function (DUF4917) | -2.51 |
| B-144 | *W909_RS08625* | hypothetical protein && - | -2.50 |
| B-145 | *W909_RS08660* | ribosomal protein S5-alanine N-acetyltransferase && PF13302:Acetyltransferase (GNAT) domain | -1.17 |
| B-146 | *W909_RS08665* | YceH family protein && PF04337:Protein of unknown function, DUF480 | -1.02 |
| B-147 | *W909_RS08670* | Gfo/Idh/MocA family oxidoreductase && PF01408:Oxidoreductase family, NAD-binding Rossmann fold | -1.05 |
| B-148 | *W909_RS08825* | YebC/PmpR family DNA-binding transcriptional regulator && PF01709:Transcriptional regulator | -1.04 |
| B-149 | *W909_RS08830* | crossover junction endodeoxyribonuclease RuvC && PF02075:Crossover junction endodeoxyribonuclease RuvC | -1.11 |
| B-150 | *W909_RS08835* | HoxN/HupN/NixA family nickel/cobalt transporter && PF03824:High-affinity nickel-transport protein | -2.08 |
| B-151 | *W909_RS09000* | D-hexose-6-phosphate mutarotase && PF01263:Aldose 1-epimerase | -1.08 |
| B-152 | *W909_RS09180* | outer membrane protein OmpW && PF03922:OmpW family | -2.48 |
| B-153 | *W909_RS09185* | porin && PF06178:Oligogalacturonate-specific porin protein (KdgM) | -1.20 |
| B-154 | *W909_RS09245* | protease SohB && PF01343:Peptidase family S49|PF08496:Peptidase family S49 N-terminal | -1.19 |
| B-155 | *W909_RS09380* | MFS transporter && PF07690:Major Facilitator Superfamily | -2.55 |
| B-156 | *W909_RS09395* | ATP-dependent dethiobiotin synthetase BioD && PF13500:AAA domain | -1.68 |
| B-157 | *W909_RS09450* | KUP/HAK/KT family potassium transporter && PF02705:K+ potassium transporter | -1.26 |
| B-158 | *W909_RS09525* | hypothetical protein && PF12902:Ferritin-like | -1.20 |
| B-159 | *W909_RS09575* | HAMP domain-containing protein && PF00672:HAMP domain|PF00015:Methyl-accepting chemotaxis protein (MCP) signalling domain | -1.23 |
| B-160 | *W909_RS09590* | FIST C-terminal domain-containing protein && PF00015:Methyl-accepting chemotaxis protein (MCP) signalling domain|PF10442:FIST C domain|PF08495:FIST N domain | -1.72 |
| B-161 | *W909_RS09610* | Re/Si-specific NAD(P)(+) transhydrogenase subunit beta && PF02233:NAD(P) transhydrogenase beta subunit | -1.28 |
| B-162 | *W909_RS09615* | Re/Si-specific NAD(P)(+) transhydrogenase subunit alpha && PF05222:Alanine dehydrogenase/PNT, N-terminal domain|PF12769:4TM region of pyridine nucleotide transhydrogenase, mitoch|PF01262:Alanine dehydrogenase/PNT, C-terminal domain | -1.26 |
| B-163 | *W909_RS09690* | xylanase && PF02055:O-Glycosyl hydrolase family 30 | -1.55 |
| B-164 | *W909_RS09850* | PTS mannose transporter subunit IIAB && PF03610:PTS system fructose IIA component|PF03830:PTS system sorbose subfamily IIB component | -1.54 |
| B-165 | *W909_RS09855* | PTS mannose/fructose/sorbose transporter subunit IIC && PF03609:PTS system sorbose-specific iic component | -1.42 |
| B-166 | *W909_RS09875* | 23S rRNA (guanine(745)-N(1))-methyltransferase && PF13847:Methyltransferase domain | -1.60 |
| B-167 | *W909_RS09880* | transcription antiterminator/RNA stability regulator CspE && PF00313:'Cold-shock' DNA-binding domain | -1.57 |
| B-168 | *W909_RS09905* | 2-dehydro-3-deoxy-D-gluconate 5-dehydrogenase KduD && PF00106:short chain dehydrogenase | -1.01 |
| B-169 | *W909_RS09915* | pectate disaccharide-lyase && PF06917:Periplasmic pectate lyase | -2.69 |
| B-170 | *W909_RS09920* | sugar ABC transporter permease && PF00528:Binding-protein-dependent transport system inner membrane component | -2.32 |
| B-171 | *W909_RS09925* | carbohydrate ABC transporter permease && PF00528:Binding-protein-dependent transport system inner membrane component | -1.34 |
| B-172 | *W909_RS09930* | sn-glycerol-3-phosphate ABC transporter ATP-binding protein UgpC && PF00005:ABC transporter | -1.15 |
| B-173 | *W909_RS09935* | carbohydrate ABC transporter substrate-binding protein && PF01547:Bacterial extracellular solute-binding protein | -1.39 |
| B-174 | *W909_RS09940* | oligogalacturonate-specific porin KdgM && PF06178:Oligogalacturonate-specific porin protein (KdgM) | -3.94 |
| B-175 | *W909_RS09990* | phenylalanine--tRNA ligase subunit alpha && PF02912:Aminoacyl tRNA synthetase class II, N-terminal domain|PF01409:tRNA synthetases class II core domain (F) | -1.17 |
| B-176 | *W909_RS09995* | 50S ribosomal protein L20 && PF00453:Ribosomal protein L20 | -1.14 |
| B-177 | *W909_RS10085* | peptidase T && PF01546:Peptidase family M20/M25/M40|PF07687:Peptidase dimerisation domain | -1.75 |
| B-178 | *W909_RS10315* | FeoC-like transcriptional regulator && PF09012:FeoC like transcriptional regulator | -1.18 |
| B-179 | *W909_RS10320* | Fe(2+) transporter permease subunit FeoB && PF02421:Ferrous iron transport protein B|PF07670:Nucleoside recognition|PF07664:Ferrous iron transport protein B C terminus | -2.58 |
| B-180 | *W909_RS10325* | ferrous iron transport protein A && PF04023:FeoA domain | -2.18 |
| B-181 | *W909_RS10375* | ShlB/FhaC/HecB family hemolysin secretion/activation protein && PF08479:POTRA domain, ShlB-type|PF03865:Haemolysin secretion/activation protein ShlB/FhaC/HecB | -1.88 |
| B-182 | *W909_RS10630* | L-threonine dehydrogenase && PF00465:Iron-containing alcohol dehydrogenase | -1.25 |
| B-183 | *W909_RS10650* | nitrate reductase subunit alpha && PF01568:Molydopterin dinucleotide binding domain|PF00384:Molybdopterin oxidoreductase|PF14710:Respiratory nitrate reductase alpha N-terminal | -1.84 |
| B-184 | *W909_RS10655* | FAD-dependent oxidoreductase && PF07992:Pyridine nucleotide-disulphide oxidoreductase|PF00724:NADH:flavin oxidoreductase / NADH oxidase family | -1.16 |
| B-185 | *W909_RS10665* | NarK family nitrate/nitrite MFS transporter && PF07690:Major Facilitator Superfamily | -1.88 |
| B-186 | *W909_RS10745* | TraR/DksA family transcriptional regulator && PF01258:Prokaryotic dksA/traR C4-type zinc finger | -2.61 |
| B-187 | *W909_RS10825* | tail fiber assembly protein && PF02413:Caudovirales tail fibre assembly protein, lambda gpK | -1.20 |
| B-188 | *W909_RS10830* | phage tail sheath protein && PF04984:Phage tail sheath protein | -1.73 |
| B-189 | *W909_RS10835* | phage major tail tube protein && PF04985:Phage tail tube protein FII | -1.67 |
| B-190 | *W909_RS10885* | hypothetical protein && - | -1.23 |
| B-191 | *W909_RS10935* | C4-dicarboxylic acid transporter DauA && PF01740:STAS domain|PF00916:Sulfate permease family | -1.05 |
| B-192 | *W909_RS10965* | L-lactate permease && PF02652:L-lactate permease | -2.92 |
| B-193 | *W909_RS10970* | (Fe-S)-binding protein && PF02754:Cysteine-rich domain | -1.63 |
| B-194 | *W909_RS10975* | iron-sulfur cluster-binding protein && PF13183:4Fe-4S dicluster domain|PF11870:Domain of unknown function (DUF3390)|PF02589:Uncharacterised ACR, YkgG family COG1556 | -1.56 |
| B-195 | *W909_RS10980* | lactate utilization protein C && PF02589:Uncharacterised ACR, YkgG family COG1556 | -1.35 |
| B-196 | *W909_RS11005* | PAS domain-containing protein && PF00015:Methyl-accepting chemotaxis protein (MCP) signalling domain|PF08447:PAS fold | -1.31 |
| B-197 | *W909_RS11090* | M20/M25/M40 family metallo-hydrolase && PF01546:Peptidase family M20/M25/M40|PF07687:Peptidase dimerisation domain | -1.07 |
| B-198 | *W909_RS11195* | carbon starvation protein A && PF02554:Carbon starvation protein CstA|PF13722:5TM C-terminal transporter carbon starvation CstA | -1.15 |
| B-199 | *W909_RS11285* | transcriptional regulator SlyA && PF01047:MarR family | -1.01 |
| B-200 | *W909_RS11340* | glycoside hydrolase && PF00756:Putative esterase|PF02922:Carbohydrate-binding module 48 (Isoamylase N-terminal domain) | -1.22 |
| B-201 | *W909_RS11360* | pyruvate kinase PykF && PF00224:Pyruvate kinase, barrel domain|PF02887:Pyruvate kinase, alpha/beta domain | -1.01 |
| B-202 | *W909_RS11405* | kinase/pyrophosphorylase && PF03618:Kinase/pyrophosphorylase | -1.77 |
| B-203 | *W909_RS11500* | porin && PF00267:Gram-negative porin | -2.26 |
| B-204 | *W909_RS11505* | porin && PF00267:Gram-negative porin | -1.24 |
| B-205 | *W909_RS11675* | PTS glucose transporter subunit IIBC && PF00367:phosphotransferase system, EIIB|PF02378:Phosphotransferase system, EIIC | -1.21 |
| B-206 | *W909_RS11845* | bifunctional 3-hydroxydecanoyl-ACP dehydratase/trans-2-decenoyl-ACP isomerase && PF07977:FabA-like domain | -1.24 |
| B-207 | *W909_RS11850* | RpiB/LacA/LacB family sugar-phosphate isomerase && PF02502:Ribose/Galactose Isomerase|PF12408:Ribose-5-phosphate isomerase | -1.15 |
| B-208 | *W909_RS12135* | chemotaxis response regulator protein-glutamate methylesterase && PF00072:Response regulator receiver domain|PF01339:CheB methylesterase | -1.05 |
| B-209 | *W909_RS12180* | methyl-accepting chemotaxis protein && PF00015:Methyl-accepting chemotaxis protein (MCP) signalling domain|PF02203:Tar ligand binding domain homologue|PF00672:HAMP domain | -1.45 |
| B-210 | *W909_RS12735* | gamma-glutamyltransferase && PF01019:Gamma-glutamyltranspeptidase | -1.21 |
| B-211 | *W909_RS12785* | ABC transporter ATP-binding protein && PF00005:ABC transporter|PF07673:Protein of unknown function (DUF1602) | -1.64 |
| B-212 | *W909_RS12790* | nitrate ABC transporter permease && PF00528:Binding-protein-dependent transport system inner membrane component | -1.89 |
| B-213 | *W909_RS12795* | ABC transporter substrate-binding protein && PF13379:NMT1-like family | -3.07 |
| B-214 | *W909_RS12815* | Tar ligand binding domain-containing protein && PF00672:HAMP domain|PF02203:Tar ligand binding domain homologue|PF00015:Methyl-accepting chemotaxis protein (MCP) signalling domain | -3.72 |
| B-215 | *W909_RS12820* | formate dehydrogenase cytochrome b556 subunit && PF00033:Cytochrome b/b6/petB | -2.09 |
| B-216 | *W909_RS12825* | formate dehydrogenase subunit beta && PF09163:Formate dehydrogenase N, transmembrane|PF13247:4Fe-4S dicluster domain | -1.93 |
| B-217 | *W909_RS12830* | formate dehydrogenase-N subunit alpha && PF00384:Molybdopterin oxidoreductase|PF01568:Molydopterin dinucleotide binding domain|PF04879:Molybdopterin oxidoreductase Fe4S4 domain | -1.64 |
| B-218 | *W909_RS12895* | Ni/Fe-hydrogenase cytochrome b subunit && - | -2.53 |
| B-219 | *W909_RS12900* | hydrogenase 2 operon protein HybA && PF13247:4Fe-4S dicluster domain | -3.25 |
| B-220 | *W909_RS12905* | hydrogenase 2 small subunit && PF14720:NiFe/NiFeSe hydrogenase small subunit C-terminal|PF01058:NADH ubiquinone oxidoreductase, 20 Kd subunit | -3.31 |
| B-221 | *W909_RS13000* | SLC13 family permease && PF02080:TrkA-C domain|PF03600:Citrate transporter | -1.39 |
| B-222 | *W909_RS13005* | sugar phosphatase && PF13419:Haloacid dehalogenase-like hydrolase | -1.52 |
| B-223 | *W909_RS13010* | YfbU family protein && PF03887:YfbU domain | -1.65 |
| B-224 | *W909_RS13160* | tripartite tricarboxylate transporter substrate binding protein && PF03401:Tripartite tricarboxylate transporter family receptor | -1.46 |
| B-225 | *W909_RS13165* | tripartite tricarboxylate transporter TctB family protein && PF07331:Tripartite tricarboxylate transporter TctB family | -1.42 |
| B-226 | *W909_RS13170* | tripartite tricarboxylate transporter permease && PF01970:Tripartite tricarboxylate transporter TctA family | -1.62 |
| B-227 | *W909_RS13180* | beta-ketoacyl-ACP synthase I && PF02801:Beta-ketoacyl synthase, C-terminal domain|PF00109:Beta-ketoacyl synthase, N-terminal domain | -1.25 |
| B-228 | *W909_RS13275* | long-chain fatty acid transporter FadL && PF03349:Outer membrane protein transport protein (OMPP1/FadL/TodX) | -1.97 |
| B-229 | *W909_RS13455* | GspS family T2SS pilot lipoprotein variant OutS && PF09691:Type II secretion system pilotin lipoprotein (PulS_OutS) | -1.33 |
| B-230 | *W909_RS13580* | cupin domain-containing protein && PF08007:Cupin superfamily protein | -3.20 |
| B-231 | *W909_RS13595* | YjfB family protein && PF14070:Putative motility protein | -1.13 |
| B-232 | *W909_RS13635* | extracellular solute-binding protein && PF13416:Bacterial extracellular solute-binding protein | -1.33 |
| B-233 | *W909_RS13640* | sn-glycerol-3-phosphate ABC transporter ATP-binding protein UgpC && PF00005:ABC transporter|PF07673:Protein of unknown function (DUF1602) | -1.04 |
| B-234 | *W909_RS13765* | methyl-accepting chemotaxis protein && PF08376:Nitrate and nitrite sensing|PF00672:HAMP domain|PF00015:Methyl-accepting chemotaxis protein (MCP) signalling domain | -1.31 |
| B-235 | *W909_RS13780* | cysteine--tRNA ligase && PF01406:tRNA synthetases class I (C) catalytic domain|PF09190:DALR domain | -1.09 |
| B-236 | *W909_RS13785* | DoxX family protein && PF07681:DoxX | -1.51 |
| B-237 | *W909_RS13790* | peptidylprolyl isomerase B && PF00160:Cyclophilin type peptidyl-prolyl cis-trans isomerase/CLD | -1.18 |
| B-238 | *W909_RS13810* | HAAAP family serine/threonine permease && PF03222:Tryptophan/tyrosine permease family | -1.66 |
| B-239 | *W909_RS13815* | L-serine ammonia-lyase && PF03315:Serine dehydratase beta chain|PF03313:Serine dehydratase alpha chain | -1.03 |
| B-240 | *W909_RS13910* | methylglyoxal synthase && PF02142:MGS-like domain | -1.85 |
| B-241 | *W909_RS13950* | NupC/NupG family nucleoside CNT transporter && PF07670:Nucleoside recognition|PF07662:Na+ dependent nucleoside transporter C-terminus|PF01773:Na+ dependent nucleoside transporter N-terminus | -1.19 |
| B-242 | *W909_RS13960* | MdtB/MuxB family multidrug efflux RND transporter permease subunit && PF00873:AcrB/AcrD/AcrF family | -1.06 |
| B-243 | *W909_RS14080* | nuclear transport factor 2 family protein && PF12680:SnoaL-like domain | -2.71 |
| B-244 | *W909_RS14085* | SDR family NAD(P)-dependent oxidoreductase && PF00106:short chain dehydrogenase | -2.52 |
| B-245 | *W909_RS14090* | FAD-dependent oxidoreductase && PF01593:Flavin containing amine oxidoreductase | -2.38 |
| B-246 | *W909_RS14095* | DUF1365 domain-containing protein && PF07103:Protein of unknown function (DUF1365) | -2.08 |
| B-247 | *W909_RS14100* | class I SAM-dependent methyltransferase && PF02353:Mycolic acid cyclopropane synthetase | -1.87 |
| B-248 | *W909_RS14105* | DUF3833 domain-containing protein && PF12915:Protein of unknown function (DUF3833) | -1.35 |
| B-249 | *W909_RS14110* | DUF523 and DUF1722 domain-containing protein && PF04463:Protein of unknown function (DUF523)|PF08349:Protein of unknown function (DUF1722) | -1.46 |
| B-250 | *W909_RS14395* | serine hydroxymethyltransferase && PF00464:Serine hydroxymethyltransferase | -1.04 |
| B-251 | *W909_RS14410* | polysaccharide lyase && PF00544:Pectate lyase | -1.45 |
| B-252 | *W909_RS14570* | autonomous glycyl radical cofactor GrcA && PF01228:Glycine radical | -1.63 |
| B-253 | *W909_RS14850* | cobalamin-independent methionine synthase II family protein && PF01717:Cobalamin-independent synthase, Catalytic domain | -1.40 |
| B-254 | *W909_RS14890* | methyl-accepting chemotaxis protein && PF00015:Methyl-accepting chemotaxis protein (MCP) signalling domain|PF02743:Cache domain|PF00672:HAMP domain | -2.48 |
| B-255 | *W909_RS14905* | asparaginase && PF00710:Asparaginase | -1.41 |
| B-256 | *W909_RS14975* | ornithine cyclodeaminase family protein && PF02423:Ornithine cyclodeaminase/mu-crystallin family | -1.25 |
| B-257 | *W909_RS15090* | murein L%2CD-transpeptidase && PF03734:L,D-transpeptidase catalytic domain | -1.06 |
| B-258 | *W909_RS15265* | multidrug efflux MFS transporter periplasmic adaptor subunit EmrA && PF00529:HlyD membrane-fusion protein of T1SS|PF16576:Barrel-sandwich domain of CusB or HlyD membrane-fusion | -1.01 |
| B-259 | *W909_RS15355* | MetQ/NlpA family lipoprotein && PF03180:NLPA lipoprotein | -1.58 |
| B-260 | *W909_RS15540* | glucarate dehydratase && PF13378:Enolase C-terminal domain-like|PF02746:Mandelate racemase / muconate lactonizing enzyme, N-terminal domain | -1.95 |
| B-261 | *W909_RS15545* | MFS transporter && PF07690:Major Facilitator Superfamily | -1.95 |
| B-262 | *W909_RS15550* | galactarate dehydratase && PF04295:D-galactarate dehydratase / Altronate hydrolase, C terminus|PF08666:SAF domain | -1.19 |
| B-263 | *W909_RS15685* | bifunctional 2'%2C3'-cyclic-nucleotide 2'-phosphodiesterase/3'-nucleotidase && PF00149:Calcineurin-like phosphoesterase|PF02872:5'-nucleotidase, C-terminal domain | -1.06 |
| B-264 | *W909_RS15725* | HAMP domain-containing protein && PF00672:HAMP domain|PF00015:Methyl-accepting chemotaxis protein (MCP) signalling domain | -2.08 |
| B-265 | *W909_RS15735* | 50S ribosomal protein L9 && PF03948:Ribosomal protein L9, C-terminal domain|PF01281:Ribosomal protein L9, N-terminal domain | -1.05 |
| B-266 | *W909_RS15740* | 30S ribosomal protein S18 && PF01084:Ribosomal protein S18 | -1.10 |
| B-267 | *W909_RS15745* | primosomal replication protein N && PF00436:Single-strand binding protein family | -1.09 |
| B-268 | *W909_RS15750* | 30S ribosomal protein S6 && PF01250:Ribosomal protein S6 | -1.11 |
| B-269 | *W909_RS15870* | PTS glucose transporter subunit IIA && PF00358:phosphoenolpyruvate-dependent sugar phosphotransferase system, EIIA 1|PF02378:Phosphotransferase system, EIIC|PF00367:phosphotransferase system, EIIB | -1.18 |
| B-270 | *W909_RS15875* | PRD domain-containing protein && PF03123:CAT RNA binding domain|PF00874:PRD domain | -1.77 |
| B-271 | *W909_RS15900* | PTS sugar transporter subunit IIB && PF02302:PTS system, Lactose/Cellobiose specific IIB subunit | -1.09 |
| B-272 | *W909_RS15905* | glycoside hydrolase family 1 protein && PF00232:Glycosyl hydrolase family 1 | -1.22 |
| B-273 | *W909_RS15910* | PTS sugar transporter subunit IIC && PF02378:Phosphotransferase system, EIIC | -1.75 |
| B-274 | *W909_RS16190* | hypothetical protein && - | -1.14 |
| B-275 | *W909_RS16195* | carbohydrate ABC transporter substrate-binding protein && PF13416:Bacterial extracellular solute-binding protein | -1.51 |
| B-276 | *W909_RS16200* | glucosamine kinase && - | -1.16 |
| B-277 | *W909_RS16245* | iron-containing alcohol dehydrogenase && PF00465:Iron-containing alcohol dehydrogenase | -2.28 |
| B-278 | *W909_RS16250* | dihydrodipicolinate synthase family protein && PF00701:Dihydrodipicolinate synthetase family | -2.28 |
| B-279 | *W909_RS16255* | sodium:solute symporter family protein && PF00474:Sodium:solute symporter family | -1.35 |
| B-280 | *W909_RS16320* | amino acid permease && PF00324:Amino acid permease | -1.32 |
| B-281 | *W909_RS16335* | nucleoside-specific channel-forming protein Tsx && PF03502:Nucleoside-specific channel-forming protein, Tsx | -1.17 |
| B-282 | *W909_RS16670* | LysE family translocator && PF01810:LysE type translocator | -1.06 |
| B-283 | *W909_RS16730* | Na+/H+ antiporter NhaA && PF06965:Na+/H+ antiporter 1 | -1.07 |
| B-284 | *W909_RS16800* | leucine--tRNA ligase && PF13603:Leucyl-tRNA synthetase, Domain 2|PF08264:Anticodon-binding domain of tRNA|PF09334:tRNA synthetases class I (M)|PF00133:tRNA synthetases class I (I, L, M and V) | -1.04 |
| B-285 | *W909_RS16805* | radical SAM protein && PF04055:Radical SAM superfamily|PF13186:Iron-sulfur cluster-binding domain | -2.11 |
| B-286 | *W909_RS16935* | MFS transporter TsgA && PF07690:Major Facilitator Superfamily | -1.08 |
| B-287 | *W909_RS16950* | methionine adenosyltransferase && PF02772:S-adenosylmethionine synthetase, central domain|PF02773:S-adenosylmethionine synthetase, C-terminal domain|PF00438:S-adenosylmethionine synthetase, N-terminal domain | -1.23 |
| B-288 | *W909_RS17140* | Tar ligand binding domain-containing protein && PF00015:Methyl-accepting chemotaxis protein (MCP) signalling domain|PF02203:Tar ligand binding domain homologue | -2.23 |
| B-289 | *W909_RS17145* | hypothetical protein && - | -1.45 |
| B-290 | *W909_RS17160* | tail fiber protein && PF07484:Phage Tail Collar Domain | -1.16 |
| B-291 | *W909_RS17330* | fumarate reductase (quinol) flavoprotein subunit && PF02910:Fumarate reductase flavoprotein C-term|PF00890:FAD binding domain | -2.25 |
| B-292 | *W909_RS17335* | succinate dehydrogenase/fumarate reductase iron-sulfur subunit && PF13183:4Fe-4S dicluster domain|PF13085:2Fe-2S iron-sulfur cluster binding domain | -1.72 |
| B-293 | *W909_RS17390* | DASS family sodium-coupled anion symporter && PF00939:Sodium:sulfate symporter transmembrane region | -1.53 |
| B-294 | *W909_RS17515* | 50S ribosomal protein L17 && PF01196:Ribosomal protein L17 | -1.30 |
| B-295 | *W909_RS17520* | DNA-directed RNA polymerase subunit alpha && PF01000:RNA polymerase Rpb3/RpoA insert domain|PF01193:RNA polymerase Rpb3/Rpb11 dimerisation domain|PF03118:Bacterial RNA polymerase, alpha chain C terminal domain | -1.17 |
| B-296 | *W909_RS17525* | 30S ribosomal protein S4 && PF00163:Ribosomal protein S4/S9 N-terminal domain|PF01479:S4 domain | -1.25 |
| B-297 | *W909_RS17530* | 30S ribosomal protein S11 && PF00411:Ribosomal protein S11 | -1.14 |
| B-298 | *W909_RS17535* | 30S ribosomal protein S13 && PF00416:Ribosomal protein S13/S18 | -1.20 |
| B-299 | *W909_RS17540* | preprotein translocase subunit SecY && PF00344:SecY translocase | -1.07 |
| B-300 | *W909_RS17545* | 50S ribosomal protein L15 && PF00828:Ribosomal protein L18e/L15 | -1.09 |
| B-301 | *W909_RS17550* | 50S ribosomal protein L30 && PF00327:Ribosomal protein L30p/L7e | -1.29 |
| B-302 | *W909_RS17555* | 30S ribosomal protein S5 && PF00333:Ribosomal protein S5, N-terminal domain|PF03719:Ribosomal protein S5, C-terminal domain | -1.14 |
| B-303 | *W909_RS17560* | 50S ribosomal protein L18 && PF00861:Ribosomal L18p/L5e family | -1.12 |
| B-304 | *W909_RS17570* | 30S ribosomal protein S8 && PF00410:Ribosomal protein S8 | -1.13 |
| B-305 | *W909_RS17585* | 50S ribosomal protein L24 && - | -1.17 |
| B-306 | *W909_RS17590* | 50S ribosomal protein L14 && PF00238:Ribosomal protein L14p/L23e | -1.17 |
| B-307 | *W909_RS17595* | 30S ribosomal protein S17 && PF00366:Ribosomal protein S17 | -1.29 |
| B-308 | *W909_RS17600* | 50S ribosomal protein L29 && PF00831:Ribosomal L29 protein | -1.47 |
| B-309 | *W909_RS17605* | 50S ribosomal protein L16 && PF00252:Ribosomal protein L16p/L10e | -1.36 |
| B-310 | *W909_RS17610* | 30S ribosomal protein S3 && PF00189:Ribosomal protein S3, C-terminal domain|PF07650:KH domain | -1.48 |
| B-311 | *W909_RS17615* | 50S ribosomal protein L22 && PF00237:Ribosomal protein L22p/L17e | -1.68 |
| B-312 | *W909_RS17620* | 30S ribosomal protein S19 && PF00203:Ribosomal protein S19 | -1.67 |
| B-313 | *W909_RS17625* | 50S ribosomal protein L2 && PF03947:Ribosomal Proteins L2, C-terminal domain|PF00181:Ribosomal Proteins L2, RNA binding domain | -1.72 |
| B-314 | *W909_RS17630* | 50S ribosomal protein L23 && PF00276:Ribosomal protein L23 | -1.67 |
| B-315 | *W909_RS17635* | 50S ribosomal protein L4 && PF00573:Ribosomal protein L4/L1 family | -1.59 |
| B-316 | *W909_RS17640* | 50S ribosomal protein L3 && PF00297:Ribosomal protein L3 | -1.70 |
| B-317 | *W909_RS17645* | 30S ribosomal protein S10 && PF00338:Ribosomal protein S10p/S20e | -1.73 |
| B-318 | *W909_RS17660* | elongation factor Tu && PF00009:Elongation factor Tu GTP binding domain|PF03144:Elongation factor Tu domain 2|PF03143:Elongation factor Tu C-terminal domain | -1.18 |
| B-319 | *W909_RS17665* | elongation factor G && PF03144:Elongation factor Tu domain 2|PF03764:Elongation factor G, domain IV|PF14492:Elongation Factor G, domain II|PF00679:Elongation factor G C-terminus|PF00009:Elongation factor Tu GTP binding domain | -1.29 |
| B-320 | *W909_RS17670* | 30S ribosomal protein S7 && PF00177:Ribosomal protein S7p/S5e | -1.17 |
| B-321 | *W909_RS17675* | 30S ribosomal protein S12 && PF00164:Ribosomal protein S12/S23 | -1.18 |
| B-322 | *W909_RS17880* | PatA/PatG family cyanobactin maturation protease && PF00082:Subtilase family | -1.58 |
| B-323 | *W909_RS17895* | polysaccharide lyase && PF00544:Pectate lyase | -1.54 |
| B-324 | *W909_RS18010* | phosphoenolpyruvate carboxykinase (ATP) && PF01293:Phosphoenolpyruvate carboxykinase | -1.27 |
| B-325 | *W909_RS18090* | ribokinase && PF00294:pfkB family carbohydrate kinase|PF08220:DeoR-like helix-turn-helix domain | -2.33 |
| B-326 | *W909_RS18095* | dihydrodipicolinate synthase family protein && PF00701:Dihydrodipicolinate synthetase family | -2.00 |
| B-327 | *W909_RS18100* | YhcH/YjgK/YiaL family protein && PF04074:Domain of unknown function (DUF386) | -2.23 |
| B-328 | *W909_RS18115* | glycerol-3-phosphate dehydrogenase && PF16901:C-terminal domain of alpha-glycerophosphate oxidase|PF01266:FAD dependent oxidoreductase | -1.67 |
| B-329 | *W909_RS18155* | nickel-responsive transcriptional regulator NikR && PF01402:Ribbon-helix-helix protein, copG family|PF08753:NikR C terminal nickel binding domain | -1.31 |
| B-330 | *W909_RS18185* | hypothetical protein && - | -1.81 |
| B-331 | *W909_RS18195* | anaerobic glycerol-3-phosphate dehydrogenase subunit C && PF02754:Cysteine-rich domain|PF13183:4Fe-4S dicluster domain | -1.45 |
| B-332 | *W909_RS18200* | glycerol-3-phosphate dehydrogenase subunit GlpB && PF00890:FAD binding domain | -1.54 |
| B-333 | *W909_RS18210* | glycerol-3-phosphate transporter && PF07690:Major Facilitator Superfamily | -2.58 |
| B-334 | *W909_RS18215* | glycerophosphodiester phosphodiesterase && PF03009:Glycerophosphoryl diester phosphodiesterase family | -1.15 |
| B-335 | *W909_RS18610* | TonB-dependent vitamin B12 receptor BtuB && PF07715:TonB-dependent Receptor Plug Domain|PF00593:TonB dependent receptor | -1.27 |
| B-336 | *W909_RS18640* | glutathione peroxidase && PF00462:Glutaredoxin|PF08534:Redoxin | -1.23 |
| B-337 | *W909_RS18665* | 50S ribosomal protein L31 && PF01197:Ribosomal protein L31 | -1.80 |
| B-338 | *W909_RS18710* | aquaporin family protein && PF00230:Major intrinsic protein | -3.05 |
| B-339 | *W909_RS18715* | glycerol kinase GlpK && PF00370:FGGY family of carbohydrate kinases, N-terminal domain|PF02782:FGGY family of carbohydrate kinases, C-terminal domain | -2.51 |
| B-340 | *W909_RS18740* | triose-phosphate isomerase && PF00121:Triosephosphate isomerase | -1.03 |
| B-341 | *W909_RS18805* | phage tail assembly chaperone && PF16778:Phage tail assembly chaperone protein | -1.44 |
| B-342 | *W909_RS18890* | DeoR/GlpR transcriptional regulator && PF00455:DeoR C terminal sensor domain|PF08220:DeoR-like helix-turn-helix domain | -1.63 |
| B-343 | *W909_RS18895* | NAD(P)-dependent oxidoreductase && PF03446:NAD binding domain of 6-phosphogluconate dehydrogenase|PF14833:NAD-binding of NADP-dependent 3-hydroxyisobutyrate dehydrogenase | -1.70 |
| B-344 | *W909_RS18920* | MCP four helix bundle domain-containing protein && PF12729:Four helix bundle sensory module for signal transduction|PF00015:Methyl-accepting chemotaxis protein (MCP) signalling domain | -1.47 |
| B-345 | *W909_RS18935* | hypothetical protein && - | -1.18 |
| B-346 | *W909_RS19005* | DUF1145 family protein && PF06611:Protein of unknown function (DUF1145) | -1.01 |
| B-347 | *W909_RS19065* | endoglucanase && PF01270:Glycosyl hydrolases family 8 | -1.13 |
| B-348 | *W909_RS19090* | cellulose synthase operon protein YhjQ && PF06564:Cellulose biosynthesis protein BcsQ | -1.28 |
| B-349 | *W909_RS19095* | cellulose biosynthesis protein BcsO && PF17037:Cellulose biosynthesis protein BcsO | -1.12 |
| B-350 | *W909_RS19175* | LacI family DNA-binding transcriptional regulator && PF00356:Bacterial regulatory proteins, lacI family|PF00532:Periplasmic binding proteins and sugar binding domain of LacI family | -1.54 |
| B-351 | *W909_RS19180* | MHS family MFS transporter && PF00083:Sugar (and other) transporter | -1.10 |
| B-352 | *W909_RS19190* | dihydrodipicolinate synthase family protein && PF00701:Dihydrodipicolinate synthetase family | -1.94 |
| B-353 | *W909_RS19195* | signal transduction protein && - | -2.73 |
| B-354 | *W909_RS19200* | FGGY-family carbohydrate kinase && PF00370:FGGY family of carbohydrate kinases, N-terminal domain|PF02782:FGGY family of carbohydrate kinases, C-terminal domain | -2.65 |
| B-355 | *W909_RS19220* | mannonate dehydratase && PF03786:D-mannonate dehydratase (UxuA) | -1.62 |
| B-356 | *W909_RS19235* | universal stress protein UspA && PF00582:Universal stress protein family | -1.38 |
| B-357 | *W909_RS19595* | F0F1 ATP synthase subunit beta && PF02874:ATP synthase alpha/beta family, beta-barrel domain|PF00306:ATP synthase alpha/beta chain, C terminal domain|PF00006:ATP synthase alpha/beta family, nucleotide-binding domain | -1.01 |
| B-358 | *W909_RS19600* | F0F1 ATP synthase subunit epsilon && PF00401:ATP synthase, Delta/Epsilon chain, long alpha-helix domain|PF02823:ATP synthase, Delta/Epsilon chain, beta-sandwich domain | -1.35 |
| B-359 | *W909_RS19605* | right-handed parallel beta-helix repeat-containing protein && PF13229:Right handed beta helix region | -1.68 |
| B-360 | *W909_RS19685* | formate-dependent uric acid utilization protein AegA && PF14691:Dihydroprymidine dehydrogenase domain II, 4Fe-4S cluster|PF07992:Pyridine nucleotide-disulphide oxidoreductase|PF13247:4Fe-4S dicluster domain | -1.95 |
| B-361 | *W909_RS20130* | YolA family protein && PF16219:Domain of unknown function (DUF4879) | -1.87 |
| B-362 | *W909_RS20145* | DUF2627 domain-containing protein && PF10736:Protein of unknown function (DUF2627) | -1.80 |
| B-363 | *W909_RS20380* | tryptophanase leader peptide && - | -1.03 |
| B-364 | *W909_RS20460* | 50S ribosomal protein L34 && PF00468:Ribosomal protein L34 | -1.02 |
| B-365 | *W909_RS20575* | DNA primase && - | -2.03 |
| B-366 | *W909_RS20845* | YnhF family membrane protein && - | -1.27 |
| B-367 | *W909_RS20925* | hypothetical protein && - | -1.57 |
